# Antenatal Care Service Utilization Among Childbearing Women at El‐Digysab Village, El‐Jazeera State, Sudan, 2023

**DOI:** 10.1155/jp/5565023

**Published:** 2026-03-27

**Authors:** Amgad Abasalih, Ibrahim A. Atia, Eyad Kashif, Mohammed Elfatih Sediek

**Affiliations:** ^1^ Faculty of Medicine, University of Khartoum, Khartoum, Sudan, uofk.edu

**Keywords:** ANC, cross-sectional, maternal, maternal health, rural, rural village, Sudan, total coverage, utilization

## Abstract

**Background:**

Antenatal care is a cornerstone of the Safe Motherhood Initiative, yet maternal mortality remains a critical issue in developing countries, including Sudan. Limited data on antenatal care utilization barriers in Sudan underscores the need for targeted studies. This research provides specific data on antenatal care utilization in El‐Digysab village, El‐Jazeera state, central Sudan.

**Methods:**

A descriptive cross‐sectional study was conducted in March 2023 among women aged 18–49 who were pregnant or had been pregnant within the last 2 years. Data were collected from 288 participants.

**Results:**

Out of 251 women, 229 (91.2%) utilized antenatal care services at least once, but only 86 (34.3%) completed the World Health Organization′s recommended four visits, whereas 22 (8.8%) had no antenatal care visits. Significant associations were found between antenatal care utilization and the number of pregnancies (Spearman′s rho = −0.181) and husbands′ education level (*p* = 0.010, Somers′s *D* = 0.12). No significant link was observed between delivery complications and antenatal care adherence (*p* = 0.579). Trust in service providers significantly influenced antenatal care utilization (*p* = 0.011, log odds ratio [OR] −21.74), while forgetting appointments was the primary barrier (70.0%).

**Conclusion:**

The study highlights the need to improve antenatal care service delivery in El‐Digysab village, emphasizing communication, education, and adherence to the World Health Organization′s guidelines. Further research on antenatal care adherence and pregnancy complications is essential to promote maternal and fetal health in Sudan.

## 1. Background

According to the World Health Organization (WHO), around a thousand women needlessly die every day as a result of complications during pregnancy, childbirth, or the 6th week following delivery. Almost all (99%) of these deaths occur in developing countries [1–3]. Antenatal care (ANC), also known as prenatal care, is described as “care given by skilled healthcare professionals to pregnant women and adolescent girls to promote the best health circumstances for both mother and baby during pregnancy” [4]. It offers the mother guidance, comfort, information, support, and assistance regarding screening programs and identifying issues that increase the risk of pregnancy [5].

ANC is one of the four pillars of the Safe Motherhood Initiative, an initiative launched in 1987 to reduce maternal mortality, draw the attention of policymakers, and involve the obstetric communities in developing and developed nations [6]. Ten years after the initiative′s launch, maternal mortality and morbidity are still important health concerns in the majority of developing countries [7]. By 2000, it was intended to reduce the maternal death rate by at least half. Maternal mortality has increased during the past 10 years, especially in sub‐Saharan Africa [8]. The reasons behind this increase vary in the literature but are centered around socioeconomic reasons and policy adherence; for instance, Maine and Rosenfield reflect it as an absence of a clear strategic focus in maternity initiatives [6], and Harrison reflects it to illiteracy and poverty [8]. All exacerbated in recent years by political unrest and the ongoing conflict in Sudan.

Evidence indicates that pregnant women who have access to high‐quality maternal health services, such as focused prenatal care, delivery‐associated care, and immediate postpartum care, can avert the majority of maternal fatalities and problems connected to pregnancy [9–11]. ANC visits aid in prompt referral to the proper healthcare facilities, ensure that all pregnant women receive tetanus toxoid (TT) immunization, iron supplements, deworming medications, and malaria prophylaxis as needed, and ensure birth preparation and complication readiness for both routine and obstetric emergencies [12, 13], as the global health community has focused on Sustainable Development Goals (SDGs).

The SDG global target is to reduce the global maternal mortality ratio to less than 70 per 100,000 live births by 2030. However, for countries with the highest maternal mortality rate, the aim is to reduce deaths to 140 per 100,000 live births. The WHO recently suggested new ANC‐related guidelines rather than the focused antenatal care (FANC) model, which recommends four visits. The initial ANC visit should occur at 12 weeks of gestational age (GA), followed by visits to a trained healthcare practitioner at 20‐, 26‐, 30‐, 34‐, 36‐, 38‐, and 40‐week GA, in accordance with these updated recommendations [4]. These updated guidelines are designed to ensure a healthy pregnancy and a simple transition to labor and delivery [4]. These recommendations are more thorough, emphasizing not only maternal and fetal screening but also counseling, maternal nutrition, and the prevention and treatment of common illnesses. Furthermore, they address support for women experiencing intimate partner violence, preventative measures for malaria and/or HIV in endemic areas, and health system interventions to improve both the utilization and quality of ANC [4].

Global data show that only 70% of pregnant women receive at least four prenatal care visits, whereas more than 87% of pregnant women obtain ANC from a qualified birth attendant at least once [14]. Additionally, only 58% of pregnant women in eastern and southern Africa obtain at least four ANC visits [14]. Significant disparities exist in how often ANC is used in rural and urban regions. This urban–rural difference in coverage of at least four ANC visits in sub‐Saharan Africa surpasses 20 percentage points [14].

However, ANC services seem to be the only solution for high morbidity and mortality among women in developing countries [15, 16]. Many barriers to the utilization of those services have been reported [15]; in addition, perceptions and beliefs have been found to affect the utilization; all this leads to a decrease in the rate of ANC services. In Sudan, only 49% of women use the services [17], which likely leads to an increase in the rates of mortality and morbidity [15, 17]. The rates of mortality were found to be higher in developing countries; according to the WHO, 99% of maternal deaths occur in rural areas [18]. Additional factors may affect the utilization of ANC services due to a lack of services, low economic status, and reduced accessibility [19], addressing the factors that affect women‐seeking behavior among rural residents. This is quite important, as there has been a continuous increase in the number of rural residents [20].

There is a lack of adequate and consistent data on ANC utilization and its barriers in Sudan, even before the current conflict that arose in 2023 [21], which hinders the efforts of any global or public interventions toward improving the services. The situation after the conflict is worse, and the small evidence on ANC and maternal health needs to be reassessed after the collapse of the health system.

More than 1.1 million pregnant women in Sudan are facing huge threats under the strain of conflict. Health centers have been destroyed or rendered inaccessible, and shortages of medical supplies and the widespread loss of health care workers have left many women without access to essential maternal services, including ANC, safe delivery assistance, and postnatal care [22].

Therefore, the findings from this study provide specific data and serve as a valuable reference to address these barriers, especially in a rural setting in Sudan. The data were collected 1 month before the conflict, which can be a reference for comparison, especially in rural settings where most of the deaths occur, according to the WHO. Continued evidence generation and coverage for all deprived areas will add to the evidence literature on maternal and child‐enhancing health interventions, leading to improved infant and maternal morbidity and mortality rates and helping reach the Millennium Development Goals for maternal and child mortality [23].

Although most recent studies associate low ANC adherence with poor socioeconomic groups [24] being further from a healthcare facility and low maternal educational level [18], the published data from Sudan is still limited [25]. Therefore, this study addresses the factors that could be used to promote ANC utilization in rural areas, as studies showed low coverage in eastern Sudan [15] and western Sudan [25].

## 2. Methods

This study is aimed at studying ANC service utilization among childbearing women at El‐Digysab village, El‐Jazeera state, Sudan. Specifically, it examines the frequency of ANC services, the quality of the service, the barriers and drivers of ANC utilization, and the associations between adherence to WHO‐recommended visit numbers and various items of the study.

### 2.1. Study Area

El‐Digysab village is located in El‐Jazeera state in the center of Sudan. It has an area of 2396 km^2^ and an estimated population of 8000, 3120 (39%) of which are women, based on the last national census in 2019. The village has one primary health facility, and the nearest hospital is 28 km from it. The main occupation of its population is farming.

### 2.2. Study Design

This descriptive cross‐sectional study was conducted among females aged 18–49 years residing in El‐Digysab village, El‐Jazeera state, central Sudan, in March 2023. The study was undertaken as part of a medical mission organized by the Khartoum Medical Students′ Association aimed at providing a range of health services to the village. Inclusion criteria encompassed all women aged 18–49 years who had been pregnant within the last 2 years or were currently pregnant with a GA exceeding 14 weeks. Women who were severely ill, unable to complete the interview, or who declined participation were excluded. The estimated targeted number was 300, so all eligible women within El‐Digysab village were included in the study in a total coverage approach for the village population, as the mission resources could not exceed the village setting. The village was divided into squares, and each group of data collectors was assigned a part and reached each house in the village. The total response count was 288, out of which, 33 were excluded as they violated our inclusion criteria of the last pregnancy within 2 years, and the other four cases violated the age criterion (less than 18 years old).

### 2.3. Study Tools

Data were collected via a self‐administered questionnaire developed and implemented through the KoboToolbox application. This questionnaire was written in Arabic, encompassing the following key areas: women′s sociodemographic information, frequency of ANC service utilization, factors influencing the utilization of ANC services (both drivers and barriers), potential obstetric complications, and an assessment of ANC service quality.

Prior to data collection, the questionnaire was rigorously pretested on 10 women and subjected to thorough quality checks to ensure reliability and validity. Data collectors from the mission participants were trained and exposed to the research aims and tools before the mission started.

Ethical approval was obtained from the Department of Community Medicine, Faculty of Medicine, University of Khartoum, as well as the local Department of Health Promotion, El‐Managel locality, El‐Jazeera state. All participants were informed about the study objectives, and verbal consent was taken from each participant before collecting the data.

### 2.4. Study Variables and Measurement

The sociodemographic category of the study tool (Table 1) was treated as a control variable against ANC utilization as a dependent variable, as well as the drivers, barriers, complications, and service quality. Each category was tested against ANC utilization. The drivers, barriers, and quality items were tested using scalable questions for each category, and the average sum of each served as an indicator for the whole category. Every item was also tested individually as a control variable against utilization status to look for an association.

**Table 1:** Respondents′ characteristics (*n* = 251).

Age (mean ± standard deviation [SD])	(26.0 + 5.5)
Educational level, *n* (%)	
Illiterate	4 (1.6%)
Primary school	99 (39.6%)
Middle school	60 (24.0)
Secondary school	82 (32.8)
Bachelor degree	5 (2.0)
Husband educational level, *n* (%)	
Illiterate	7 (2.8%)
Primary school	94 (37.6%)
Middle school	56 (22.4%)
Secondary school	66 (26.4%)
Bachelor degree	14 (5.6%)
Diploma	6 (2.4%)
Khalwa (religious Islamic education)	6 (2.4%)
Others	1 (0.4%)
Occupation, *n* (%)	
Housewife	229 (91.2%)
Free work	14 (5.6%)
Others	8 (3.2%)
Husband occupation, *n* (%)	
I do not work	5 (2.0%)
Free work	235 (93.6%)
Other	4 (1.6%)
Student	1 (0.4%)
Governmental employee	3 (1.2%)
Private sector employee	3 (1.2%)
Number of pregnancies (mean ± SD)	(4.8 + 2.6)
Number of abortions (mean ± SD)	(0.6 + 0.9)
Number of children born alive (mean ± SD)	(4.1 + 2.4)

The complication category (Table 2) was developed using the most cited complications in ANC literature in a dichotomous manner, and each complication was tested as a control variable against ANC adherence.

**Table 2:** Complications, *n* (%).

Anemia	23 (9.2%)
Cramps	7 (2.8%)
Other pregnancy‐related illnesses	10 (4%)
Preterm delivery	23 (9.2%)
Obstructed labor	20 (8%)
Low birth weight	21 (8.4%)
Postpartum hemorrhage	11 (4.4%)
Impaired fetal growth	7 (2.8%)
Fetal death	18 (7.2%)

### 2.5. Data Management and Analysis

The questionnaire was translated into English for manuscript writing. Data mangling and analysis were conducted via Statistical Package for Social Science (SPSS) Version 26 and Microsoft Excel. The final dataset included 251 respondents. Descriptive and frequency analyses were employed to analyze categorical and numerical data, with the results summarized in tabular format presented as mean (SD) and percentages, respectively. Drivers and barriers, as long as service quality items were assessed as scale items and were tested for reliability using the Cronbach alpha value. The ANC utilization status was determined according to the WHO recommendation of four visits, which is the adopted policy by the local health authority in the state. ANC utilization status was subsequently examined against different numerical and categorical data to check for associations. Statistically significant associations (with a *p* value less than 0.05) were subjected to further regression analysis via the log odds ratio, Spearman′s rho, and Somers′s *D* value according to the variable type.

## 3. Results

### 3.1. Sociodemographic Characteristics

This cross‐sectional study included a final dataset of 251 participants with a mean age of 26.01 years (SD = 5.48), ranging from 18 to 45 years. Over a third of them (34.8%) had attained secondary or higher education, whereas 62.8% of their husbands had an education level below secondary school. The majority of participants (91.2%) were housewives. The participants reported an average of 4.82 pregnancies (SD = 2.62), with a mean of 0.51 miscarriages (SD = 0.92) and 4.25 living children (SD = 2.41) (Table 1 summarizes the respondents′ demographics).

### 3.2. ANC Utilization

Out of 229 women (91.2%) who utilized ANC services at least once, only 86 women (34.3%) adhered to the recommended four ANC visits per the WHO guidelines, whereas 8.8% had not attended at all, indicating limited engagement with ANC services. The average time of the first ANC visit was 4.67 months (SD = 0.66).

A one‐sample *t*‐test revealed a statistically significant difference between the observed mean number of ANC visits and the recommended four visits, indicating inadequate ANC utilization among the participants.

### 3.3. Factors Associated With ANC Utilization

Crosstab analysis revealed significant associations between the number of pregnancies and ANC utilization (Spearman′s rho = −0.181). Additionally, there was a weak positive association between husbands′ education level and ANC utilization (*p* = 0.010, Somers′s *D* = 0.12).

### 3.4. Maternal and Fetal Complications

A total of 251 cases were analyzed to examine the associations between various maternal complications during the last pregnancy and adherence to WHO ANC recommendations (Table 2 summarizes the complication prevalence).

The majority of participants did not experience anemia (90.8%) or hypertension (97.2%) in their last pregnancy. No significant association was found between the last and ANC adherence, with *χ* ^2^ = 0.003 and *p* = 0.956 for anemia and *χ* ^2^ = 1.651 and *p* = 0.199 for hypertension. Ten participants (4%) experienced other illnesses during their last pregnancy, and 10 participants (4%) reported symptoms related to thyroid gland problems. No significant association was found between these illnesses and ANC adherence (*χ* ^2^ = 0.074, *p* = 0.785).

### 3.5. Delivery Complications

A total of 22.3% of the respondents reported delivery complications, the largest percentage reported. No significant association was found between delivery complications and ANC adherence (*p* = 0.579).

Other complications were highlighted, such as preterm delivery (8.4%) (*p* = 0.956), low birth weight (8.4%) (*p* = 0.938), obstructed labor (8%) (*p* = 0.694), postpartum hemorrhage (4.4%) (*p* = 0.623), impaired fetal growth (2.4%) (*p* = 0.623), and fetal death (7.2%) (*p* = 0.951).

### 3.6. Drivers and Barriers to ANC Utilization

The drivers and barriers to ANC utilization were assessed using 16 questions (Table 3 summarizes the drivers and barriers). Items demonstrated acceptable reliability (Cronbach′s alpha = 0.643). The participants identified the perceived importance of ANC follow‐up and trust in service providers as the most frequent drivers (91.2%). Notably, 94.4% of the participants believed that ANC services positively impacted their health, and over 85.2% reported a good perception of ANC from their partners, family, and peers.

**Table 3:** Drivers and barriers, *n* (%).

Item	Yes	No	Neutral	Do not know/not applicable^a^
Drivers				
ANC positively affects the health of the mother and child	236 (94.4%)	3 (1.2%)	9 (3.6%)	2 (0.8%)
Do you get pregnancy advice from family/friends	**113 (45%)**	**110 (43.8%)**	26 (10.4%)	2 (0.8%)
Is the duration of ANC visits short and fast	**106 (42.2%)**	**90 (35.9%)**	49 (19.5%)	6 (2.4%)
Are the ANC facility working hours suitable for you	208 (83.5%)	21 (8.4%)	11 (4.4%)	9 (3.6%)
Do you trust in ANC service providers	229 (92.0%)	7 (2.8%)	7 (2.8%)	6 (2.4%)
Do the service providers understand your health problems	227 (91.2%)	6 (2.4%)	9 (3.6%)	7 (2.8%)
Do you have someone to care for your children during ANC visits	211 (84.7%)	11 (4.4%)	7 (2.8%)	20 (8.0%)
Barriers				
Do you regularly forget the schedule of the visits	**52 (20.8%)**	175 (70.0%)	11 (4.4%)	12 (4.8%)
Do you wait long times at ANC visits	**144 (57.6%)**	**60 (24.0%)**	39 (15.6%)	7 (2.8%)
Do you have other commitments with priority to ANC visits	37 (14.9%)	198 (79.5%)	10 (4.0%)	4 (1.6%)
Does your husband have a negative perception of ANC visits	21 (8.4%)	225 (89.6%)	4 (1.6%)	1 (0.4%)
Does your mother have a negative perception of ANC visits	17 (6.8%)	226 (90.4%)	3 (1.2%)	4 (1.6%)
Do your peers have a negative perception of ANC visits	32 (12.8%)	213 (85.2%)	4 (1.6%)	1 (0.4%)
Is reaching the ANC facility take much time from you	**98 (39.2%)**	128 (51.2%)	18 (7.2%)	6 (2.4%)
Is reaching the ANC facility cost much money from you	**99 (39.6%)**	**108 (45.6%)**	37 (14.8%)	6 (2.4%)
Is reaching the ANC facility physically exhausting	**93 (37.2%)**	140 (56.0%)	10 (4.0%)	7 (2.8%)

*Note:* Values in bold indicate the following: in drivers, less than 50%, yes; more than 30%, neutral; more than 20%, no. In barriers, less than 50%, no; more than 30%, neutral; more than 20%, yes.

^a^Not applicable responses have been added to the “do not know column” items included, such as “do not have another child”.

The primary barriers to ANC follow‐up were forgetting scheduled appointments (70.0%) and long waiting times (57.6%). Among the identified factors, trust in service providers was the only variable that was significantly related to ANC utilization (*p* = 0.011, log OR −21.74).

### 3.7. Quality of ANC Services

The quality of ANC services was examined using a 17‐item Likert scale (Table 4 summarizes the service quality items). Those scale questions demonstrated acceptable reliability (Cronbach′s alpha = 0.673). The items with the lowest mean scores were providing information on emergency approaches (mean = 1.39), preparation for delivery (mean = 1.39), and breastfeeding guidance (mean = 1.30). In contrast, the highest‐rated items were blood pressure tests (mean = 3.67), blood tests (mean = 3.64), and urine tests (mean = 3.68).

**Table 4:** Service quality during the last pregnancy visit (%).

Item	Every/most times	I do not know/remember	Few times/never
Has your weight been measured	**(13.6%)**	(1.8%)	**(84.6%)**
Has your pressure been measured	(88.6%)	(0.9%)	(10.5%)
Has your urine been tested	(87.3%)	(0.9%)	(11.8%)
Has your blood been tested	(89.0%)	(0.9%)	(10.1%)
Did you understand the purpose of the investigations being done	(53.8%)	(2.6%)	**(43.2%)**
Did you understand the purpose of the medications being given	(75.5%)	(1.3%)	**(21.9%)**
Have you been told of the normal changes during pregnancies and delivery	(64.9%)	(1.3%)	**(33.8%)**
Have you been told about signs of pregnancy complications	**(41.0%)**	(0.9%)	**(58.1%)**
Have you been told where to go if a complication happened	**(41.6%)**	(3.5%)	**(54.9%)**
Have you been guided on delivery preparation	**(38.3%)**	(0.9%)	**(60.8%)**
Have you been given information about nutrition	(62.3%)	(1.3%)	**(36.4%)**
Have you been given information about breastfeeding	**(30.3%)**	(0.4%)	**(69.3%)**
Have you been able to ask questions	(83.6%)	(0.4%)	(16.0%)
Have you been discussing your problems with privacy	(80.2%)	(0.4%)	(19.3%)
Has the provider treated you with respect	(92.1%)	(0.4%)	(7.5%)
Has the provider mistreated you due to personal difference^a^	(14.5%)	(1.8%)	(83.8%)
Have you felt that the facility was clean	(88.2%)	(1.3%)	(10.6%)

*Note:* Values in bold indicate the following: less than 50%, every/most times; more than 30%, I do not know/remember; more than 20%, few times/never.

^a^This item is flagged oppositely since the question measures the quality negatively.

When the quality items were tested against ANC utilization, providing information on nutrition was the only item that showed a statistically significant association (*p* = 0.018, log OR −0.020).

## 4. Discussion

This study is aimed at assessing ANC service utilization and its association with maternal and fetal complications in El‐Digysab village, Sudan, besides the service quality, drivers, and barriers to utilization. Although previous studies from eastern Sudan (Kassala) [15] and central Sudan (Khartoum) [26] linked pregnancy complications to a lack of ANC services, our findings did not reveal a statistically significant association between ANC adherence (defined as meeting the WHO recommendation of four visits) and most complications. This discrepancy may be attributed to several factors. First, the evolving standards of ANC utilization, which are influenced by study timelines and local guidelines, could play a significant role. For example, a prior Kassala study classified sufficient ANC as only two visits [15], potentially influencing the significance level of their findings. Second, despite limited ANC adherence, our study revealed complication rates (anemia, obstructed labor, and low birth weight) comparable with those reported in eastern and central Sudan [15, 26], suggesting that these complications might be prevalent across the country, irrespective of ANC adherence, and the significant difference in the association is due to the number of visits. Furthermore, the relatively small respondent numbers may underline this difference.

Although 34.3% of participants adhered to the WHO recommendation of four ANC visits, this number is considerably higher than the 11% reported in Kassala state [15] and 29% reported by the national ministry of health in 2022 [21], yet still less than the global number [14]. This highlights the need for improved awareness campaigns to address misconceptions and enhance awareness about the benefits and implications of ANC, promoting adherence to recommended visit schedules and polices. Furthermore, updating local healthcare facilities with current ANC guidelines and ensuring that they have the resources to accommodate the recommended visit frequency are crucial. This inadequate utilization aligned with a cross‐sectional finding from Ethiopia, which indicate linked living in rural areas as a predictor to adherence to the four‐visit recommendation of WHO [27].

Our study revealed a significant negative association between the number of pregnancies and ANC utilization. This suggests that women with more pregnancies might have lower utilization rates. This finding aligned with other studies from the region: Kenya , Ethiopia, and Nigeria [27–29], which linked parity to utilization of ANC. Further qualitative studies are needed to explore the reasons behind this association and develop interventions to mitigate it. The study also confirmed previous findings that a husband′s education level positively influences ANC utilization [15]. This emphasizes the importance of involving partners in maternal healthcare education.

Despite positive perceptions of ANC services and their impact on health, our findings revealed communication gaps and educational deficiencies. Fewer than half of the women understood the purpose of the tests and medications they received during ANC visits. Improved communication strategies are needed to ensure that women comprehend the rationale behind ANC procedures. Furthermore, more than one‐third of the women did not receive information about normal pregnancy changes, a percentage slightly higher than the findings in another study conducted in the same state but in an urban setting, where 25.5% never been told about it [30]. This also highlights the urban–rural disparities. In addition, more than half lacked guidance on the signs of complications and where to seek help. Delivery preparation and breastfeeding education were also inadequate. These findings highlight the need for a more comprehensive ANC service approach that prioritizes patient education and informed decision‐making.

The study also identified positive aspects of ANC service delivery in El‐Digysab village. Medical investigations have received a high evaluation, supporting other studies about service quality in Africa [31, 32]. A high proportion of women felt comfortable asking questions and discussing problems privately during visits, indicating a foundation for open communication between providers and patients. Respectful and fair treatment by providers was reported positively. Facility cleanliness was also satisfactory. These positive aspects provide a strong basis for building a more informative and comprehensive ANC service program.

Future interventions should focus on enhancing communication regarding the purpose of tests and medications, ensuring comprehensive information about pregnancy and potential complications, and providing guidance on delivery preparation and breastfeeding. Strengthening educational components within ANC services is also crucial to empower women with knowledge about pregnancy, potential complications, and self‐care practices. More quality interventions like an appointment system may also be adopted to mitigate the waiting barrier. Further studies should include the healthcare provider′s perspective on the barriers and services to draw a full picture for interventions. Experimental studies should accompany the interventions to ensure good outcomes and adherence. Furthermore, we suggest a large coverage study to assess ANC utilization after the conflict and compare it with the available literature before and use the comparison as a reference for future interventions.

## 5. Study Limitation

We encountered a limitation in that few studies have highlighted the association between ANC utilization and complications during pregnancy in Sudan, as most researchers have emphasized utilization coverage only which limits our discussion domain to compare the findings. Therefore, we recommend that more investigations and studies focus on the associations between ANC adherence and pregnancy complications, which may lead to the promotion of maternal and fetal health.

The cross‐sectional design limits the establishment of causal relationships between ANC utilization and pregnancy complications. Another limitation of this descriptive study is the small sample size despite covering the whole village—as an approach to minimize the bias of the village selection—which may affect the generalizability of the study findings. This is due to limited resources and the inapplicability to conduct such surveillances separately not as part of other small projects as the mission in our case. But, in a low‐income country like Sudan, such approaches still provide a valuable piece of evidence that could fill the huge missing parts of our public health story.

## 6. Conclusion

This study highlights the need for improved ANC service delivery in El‐Digysab village, which may also be the case for similar rural areas in Sudan. Surprisingly count for the living for 63.66% of Sudan′s population in 2023 [33]– bringing attention to the need for similar surveillances to assess these disparities in rural areas. There is limited evidence in ANC services in Sudan, though the findings are quite comparable across different regions in Sudan. We call for a wide range of well‐supported research to assess ANC services across Sudan and then a systematic approach to improve it, with a specific focus on communication, education, and adherence to the global health policies. Building upon the existing strengths, such as respctful care and facility usual improvments ‐ as percived from our population‐, a more comprehensive ANC program could focus on empowering women, improving pregnancy outcomes, and contributing to better maternal and fetal health while keeping those strengths. Moreover, this study highlights urgent needs for targeted interventions—such as community‐based education and improved physical access—to enhance ANC utilization in rural Sudan. These findings are especially critical for policymakers and NGOs operating in conflict‐affected areas where maternal health services are increasingly fragile. A new assessment shall be made after the conflict to support such findings of our research and similar ones before the conflict.

**Nomenclature**
ANC: antenatal care
WHO: World Health Organization
SD: standard deviation
OR: odds ratio

## Funding

No funding was received for this manuscript.

## Ethics Statement

This study received ethical approval from the Federal Ministry of Health, the El‐Jazeera Ministry of Health, and the El‐Managel health locality (the head locality of the village). Prior to data collection, the research purpose and objectives were explained to all participants clearly and concisely. Informed consent was obtained from each participant verbally.

## Conflicts of Interest

The authors declare no conflicts of interest.

## Supporting information


**Supporting Information 1** Additional supporting information can be found online in the Supporting Information section. Supporting Information A file containing all the data sheets and analysis outputs generated in the study, including the tests, frequencies, and descriptive statistics.

## Data Availability

All data generated or analyzed during this study are included in this published article (and its supporting information files).
