# Supplementary material for: Antenatal Care Service Utilization Among Childbearing Women at El‐Digysab Village, El‐Jazeera State, Sudan, 2023
Source: J Pregnancy. 2026 Mar 27;2026:5565023. doi: 10.1155/jp/5565023 (PMC13140838; doi:10.1155/jp/5565023)
Supplement: Supplementary file 1 — Supporting Information 1 Additional supporting information can be found online in the Supporting Information section. Supporting Information A file containing all the data sheets and analysis outputs generated in the study, including the tests, frequencies, and descriptive statistics. [file JP-2026-5565023-s001.zip › Revised analysis output/Revised copy of anc vs demographics.docx]

shorGET

FILE='C:\Users\hp\Downloads\ANC cleaned.sav'.

DATASET NAME DataSet1 WINDOW=FRONT.

CROSSTABS

/TABLES=ANC_utilization_status BY

- What is your level of education?
- What is your husband's level of education?
- What is your occupation?
- What is your husband's occupation?
- How many times have you been pregnant?
- How many times have you experienced a miscarriage?
- How many living children have you given birth to?

/FORMAT=AVALUE TABLES

/STATISTICS=CHISQ CORR

/CELLS=COUNT

/COUNT ROUND CELL.

**Crosstabs**

| **Notes** | | |
| --- | --- | --- |
| Output Created | | 11-DEC-2024 22:46:48 |
| Comments | |  |
| Input | Data | C:\Users\hp\Downloads\ANC cleaned.sav |
|  | Active Dataset | DataSet1 |
|  | Filter | <none> |
|  | Weight | <none> |
|  | Split File | <none> |
|  | N of Rows in Working Data File | 251 |
| Missing Value Handling | Definition of Missing | User-defined missing values are treated as missing. |
|  | Cases Used | Statistics for each table are based on all the cases with valid data in the specified range(s) for all variables in each table. |
| Syntax | | CROSSTABS  /TABLES=ANC_utilization_status BY   - What is your level of education? - What is your husband's level of education? - What is your occupation? - What is your husband's occupation? - How many times have you been pregnant? - How many times have you experienced a miscarriage? - How many living children have you given birth to?   /FORMAT=AVALUE TABLES  /STATISTICS=CHISQ CORR  /CELLS=COUNT  /COUNT ROUND CELL. |
| Resources | Processor Time | 00:00:00.02 |
|  | Elapsed Time | 00:00:07.29 |
|  | Dimensions Requested | 2 |
|  | Cells Available | 524245 |

[DataSet1] C:\Users\hp\Downloads\ANC cleaned.sav

| **Warnings** |
| --- |
| CORR statistics are available for numeric data only. |
| CORR statistics are available for numeric data only. |
| CORR statistics are available for numeric data only. |
| CORR statistics are available for numeric data only. |
| CORR statistics are available for numeric data only. |
| CORR statistics are available for numeric data only. |
| CORR statistics are available for numeric data only. |

| **Case Processing Summary** | | | | | | |
| --- | --- | --- | --- | --- | --- | --- |
|  | Cases | | | | | |
|  | Valid | | Missing | | Total | |
|  | N | Percent | N | Percent | N | Percent |
| ANC_utilization_status * What is your level of education? | 251 | 100.0% | 0 | 0.0% | 251 | 100.0% |
| ANC_utilization_status * What is your husband's level of education? | 251 | 100.0% | 0 | 0.0% | 251 | 100.0% |
| ANC_utilization_status * What is your occupation? | 251 | 100.0% | 0 | 0.0% | 251 | 100.0% |
| ANC_utilization_status * What is your husband's occupation? | 251 | 100.0% | 0 | 0.0% | 251 | 100.0% |
| ANC_utilization_status * How many times have you been pregnant? | 251 | 100.0% | 0 | 0.0% | 251 | 100.0% |
| ANC_utilization_status * How many times have you experienced a miscarriage? | 251 | 100.0% | 0 | 0.0% | 251 | 100.0% |
| ANC_utilization_status * How many living children have you given birth to? | 251 | 100.0% | 0 | 0.0% | 251 | 100.0% |

**ANC_utilization_status * What is your level of education?**

| **Crosstab** | | | | | | | | |
| --- | --- | --- | --- | --- | --- | --- | --- | --- |
| Count | | | | | | | | |
|  | | What is your level of education? | | | | | | Total |
|  | | illiterate | primary school | middle school | secondary school | bachelor degree | 99 |  |
| ANC_utilization_status | no | 4 | 73 | 39 | 46 | 3 | 0 | 165 |
|  | yes | 0 | 26 | 21 | 36 | 2 | 1 | 86 |
| Total | | 4 | 99 | 60 | 82 | 5 | 1 | 251 |

| **Chi-Square Tests** | | | |
| --- | --- | --- | --- |
|  | Value | df | Asymptotic Significance (2-sided) |
| Pearson Chi-Square | 10.287^a^ | 5 | .067 |
| Likelihood Ratio | 11.785 | 5 | .038 |
| N of Valid Cases | 251 |  |  |
| a. 6 cells (50.0%) have expected count less than 5. The minimum expected count is .34. | | | |

| **Symmetric Measures^a^** | |
| --- | --- |
|  | Value |
| N of Valid Cases | 251 |
| a. Correlation statistics are available for numeric data only. | |

**ANC_utilization_status * What is your husband's level of education?**

| **Crosstab** | | | | | | | | | | | |
| --- | --- | --- | --- | --- | --- | --- | --- | --- | --- | --- | --- |
| Count | | | | | | | | | | | |
|  | | What is your husband's level of education? | | | | | | | | | Total |
|  | | illiterate | primary school | middle school | secondary school | bachelor | diploma | (Khalwa) | others | 99 |  |
| ANC_utilization_status | no | 7 | 68 | 38 | 34 | 11 | 1 | 4 | 1 | 1 | 165 |
|  | yes | 0 | 26 | 18 | 32 | 3 | 5 | 2 | 0 | 0 | 86 |
| Total | | 7 | 94 | 56 | 66 | 14 | 6 | 6 | 1 | 1 | 251 |

| **Chi-Square Tests** | | | |
| --- | --- | --- | --- |
|  | Value | df | Asymptotic Significance (2-sided) |
| Pearson Chi-Square | 19.990^a^ | 8 | .010 |
| Likelihood Ratio | 22.445 | 8 | .004 |
| N of Valid Cases | 251 |  |  |
| a. 11 cells (61.1%) have expected count less than 5. The minimum expected count is .34. | | | |

| **Symmetric Measures^a^** | |
| --- | --- |
|  | Value |
| N of Valid Cases | 251 |
| a. Correlation statistics are available for numeric data only. | |

**ANC_utilization_status * What is your occupation?**

| **Crosstab** | | | | | |
| --- | --- | --- | --- | --- | --- |
| Count | | | | | |
|  | | What is your occupation? | | | Total |
|  | | 0 | Free work | other |  |
| ANC_utilization_status | no | 150 | 9 | 6 | 165 |
|  | yes | 79 | 5 | 2 | 86 |
| Total | | 229 | 14 | 8 | 251 |

| **Chi-Square Tests** | | | |
| --- | --- | --- | --- |
|  | Value | df | Asymptotic Significance (2-sided) |
| Pearson Chi-Square | .323^a^ | 2 | .851 |
| Likelihood Ratio | .340 | 2 | .844 |
| N of Valid Cases | 251 |  |  |
| a. 2 cells (33.3%) have expected count less than 5. The minimum expected count is 2.74. | | | |

| **Symmetric Measures^a^** | |
| --- | --- |
|  | Value |
| N of Valid Cases | 251 |
| a. Correlation statistics are available for numeric data only. | |

**ANC_utilization_status * What is your husband's occupation?**

| **Crosstab** | | | | | | | | |
| --- | --- | --- | --- | --- | --- | --- | --- | --- |
| Count | | | | | | | | |
|  | | What is your husband's occupation? | | | | | | Total |
|  | | I do not work | free work | other | student | governmental employee | privite sector employee |  |
| ANC_utilization_status | no | 4 | 154 | 1 | 1 | 2 | 3 | 165 |
|  | yes | 1 | 81 | 3 | 0 | 1 | 0 | 86 |
| Total | | 5 | 235 | 4 | 1 | 3 | 3 | 251 |

| **Chi-Square Tests** | | | |
| --- | --- | --- | --- |
|  | Value | df | Asymptotic Significance (2-sided) |
| Pearson Chi-Square | 5.489^a^ | 5 | .359 |
| Likelihood Ratio | 6.623 | 5 | .250 |
| N of Valid Cases | 251 |  |  |
| a. 10 cells (83.3%) have expected count less than 5. The minimum expected count is .34. | | | |

| **Symmetric Measures^a^** | |
| --- | --- |
|  | Value |
| N of Valid Cases | 251 |
| a. Correlation statistics are available for numeric data only. | |

**ANC_utilization_status * How many times have you been pregnant?**

| **Crosstab** | | | | | | | | | | | | | | | |
| --- | --- | --- | --- | --- | --- | --- | --- | --- | --- | --- | --- | --- | --- | --- | --- |
| Count | | | | | | | | | | | | | | | |
|  | | How many times have you been pregnant? | | | | | | | | | | | | | Total |
|  | | 1 | 2 | 3 | 4 | 5 | 6 | 7 | 8 | 9 | 10 | 11 | 12 | 13 |  |
| ANC_utilization_status | no | 11 | 18 | 18 | 21 | 26 | 26 | 15 | 17 | 5 | 2 | 3 | 1 | 2 | 165 |
|  | yes | 13 | 11 | 21 | 9 | 6 | 8 | 6 | 4 | 4 | 3 | 1 | 0 | 0 | 86 |
| Total | | 24 | 29 | 39 | 30 | 32 | 34 | 21 | 21 | 9 | 5 | 4 | 1 | 2 | 251 |

| **Chi-Square Tests** | | | |
| --- | --- | --- | --- |
|  | Value | df | Asymptotic Significance (2-sided) |
| Pearson Chi-Square | 22.496^a^ | 12 | .032 |
| Likelihood Ratio | 23.424 | 12 | .024 |
| N of Valid Cases | 251 |  |  |
| a. 9 cells (34.6%) have expected count less than 5. The minimum expected count is .34. | | | |

| **Symmetric Measures^a^** | |
| --- | --- |
|  | Value |
| N of Valid Cases | 251 |
| a. Correlation statistics are available for numeric data only. | |

**ANC_utilization_status * How many times have you experienced a miscarriage?**

| **Crosstab** | | | | | | | |
| --- | --- | --- | --- | --- | --- | --- | --- |
| Count | | | | | | | |
|  | | How many times have you experienced a miscarriage? | | | | | Total |
|  | | 0 | 1 | 2 | 3 | 4 |  |
| ANC_utilization_status | no | 101 | 37 | 19 | 6 | 2 | 165 |
|  | yes | 55 | 17 | 11 | 1 | 2 | 86 |
| Total | | 156 | 54 | 30 | 7 | 4 | 251 |

| **Chi-Square Tests** | | | |
| --- | --- | --- | --- |
|  | Value | df | Asymptotic Significance (2-sided) |
| Pearson Chi-Square | 2.011^a^ | 4 | .734 |
| Likelihood Ratio | 2.184 | 4 | .702 |
| N of Valid Cases | 251 |  |  |
| a. 4 cells (40.0%) have expected count less than 5. The minimum expected count is 1.37. | | | |

| **Symmetric Measures^a^** | |
| --- | --- |
|  | Value |
| N of Valid Cases | 251 |
| a. Correlation statistics are available for numeric data only. | |

**ANC_utilization_status * How many living children have you given birth to?**

| **Crosstab** | | | | | | | | | | | | | | | |
| --- | --- | --- | --- | --- | --- | --- | --- | --- | --- | --- | --- | --- | --- | --- | --- |
| Count | | | | | | | | | | | | | | | |
|  | | How many living children have you given birth to? | | | | | | | | | | | | | Total |
|  | | 0 | 1 | 2 | 3 | 4 | 5 | 6 | 7 | 8 | 9 | 10 | 11 | 99 |  |
| ANC_utilization_status | no | 7 | 15 | 24 | 17 | 20 | 24 | 30 | 11 | 8 | 4 | 1 | 3 | 1 | 165 |
|  | yes | 2 | 14 | 17 | 15 | 11 | 6 | 9 | 4 | 2 | 2 | 1 | 0 | 3 | 86 |
| Total | | 9 | 29 | 41 | 32 | 31 | 30 | 39 | 15 | 10 | 6 | 2 | 3 | 4 | 251 |

| **Chi-Square Tests** | | | |
| --- | --- | --- | --- |
|  | Value | df | Asymptotic Significance (2-sided) |
| Pearson Chi-Square | 17.228^a^ | 12 | .141 |
| Likelihood Ratio | 18.292 | 12 | .107 |
| N of Valid Cases | 251 |  |  |
| a. 10 cells (38.5%) have expected count less than 5. The minimum expected count is .69. | | | |

| **Symmetric Measures^a^** | |
| --- | --- |
|  | Value |
| N of Valid Cases | 251 |
| a. Correlation statistics are available for numeric data only. | |
