# Supplementary material for: Antenatal Care Service Utilization Among Childbearing Women at El‐Digysab Village, El‐Jazeera State, Sudan, 2023
Source: J Pregnancy. 2026 Mar 27;2026:5565023. doi: 10.1155/jp/5565023 (PMC13140838; doi:10.1155/jp/5565023)
Supplement: Supplementary file 1 — Supporting Information 1 Additional supporting information can be found online in the Supporting Information section. Supporting Information A file containing all the data sheets and analysis outputs generated in the study, including the tests, frequencies, and descriptive statistics. [file JP-2026-5565023-s001.zip › Revised analysis output/Revised copy of ANC Utilization vs nominal Chi squere.docx]

CROSSTABS

/TABLES=

- Who was the healthcare provider during your pregnancy?
- What type of healthcare services were provided?
- Do you have health insurance?
- What means of transportation do you use to reach the maternity healthcare center?
- Was the circumcision procedure performed on you?

BY

WHO_recommendation_for_ANC_visits

/FORMAT=AVALUE TABLES

/STATISTICS=CHISQ CC

/CELLS=COUNT EXPECTED

/COUNT ROUND CELL.

**Crosstabs**

| **Notes** | | |
| --- | --- | --- |
| Output Created | | 05-MAY-2023 19:19:31 |
| Comments | |  |
| Input | Data | C:\Users\hp\Documents\elmnagel medical day\SPSS DATA\ANC CLEANED Eyad.sav |
|  | Active Dataset | DataSet1 |
|  | Filter | <none> |
|  | Weight | <none> |
|  | Split File | <none> |
|  | N of Rows in Working Data File | 251 |
| Missing Value Handling | Definition of Missing | User-defined missing values are treated as missing. |
|  | Cases Used | Statistics for each table are based on all the cases with valid data in the specified range(s) for all variables in each table. |
| Syntax | | CROSSTABS  /TABLES=   - Who was the healthcare provider during your pregnancy? - What type of healthcare services were provided? - Do you have health insurance? - What means of transportation do you use to reach the maternity healthcare center? - Was the circumcision procedure performed on you?   BY  WHO_recommendation_for_ANC_visits  /FORMAT=AVALUE TABLES  /STATISTICS=CHISQ CC  /CELLS=COUNT EXPECTED  /COUNT ROUND CELL. |
| Resources | Processor Time | 00:00:00.02 |
|  | Elapsed Time | 00:00:00.08 |
|  | Dimensions Requested | 2 |
|  | Cells Available | 524245 |

| **Case Processing Summary** | | | | | | |
| --- | --- | --- | --- | --- | --- | --- |
|  | Cases | | | | | |
|  | Valid | | Missing | | Total | |
|  | N | Percent | N | Percent | N | Percent |
| Who was the healthcare provider during your pregnancy?* WHO_recommendation_for_ANC_visits | 191 | 76.1% | 60 | 23.9% | 251 | 100.0% |
| What type of healthcare services were provided?* WHO_recommendation_for_ANC_visits | 228 | 90.8% | 23 | 9.2% | 251 | 100.0% |
| Do you have health insurance?* WHO_recommendation_for_ANC_visits | 251 | 100.0% | 0 | 0.0% | 251 | 100.0% |
| What means of transportation do you use to reach the maternity healthcare center?* WHO_recommendation_for_ANC_visits | 218 | 86.9% | 33 | 13.1% | 251 | 100.0% |
| Was the circumcision procedure performed on you?* WHO_recommendation_for_ANC_visits | 251 | 100.0% | 0 | 0.0% | 251 | 100.0% |

**Who was the healthcare provider during your pregnancy?* WHO_recommendation_for_ANC_visits**

| **Crosstab** | | | | | |
| --- | --- | --- | --- | --- | --- |
|  | | | WHO_recommendation_for_ANC_visits | |  |
|  | | | no | yes |  |
| Who was the healthcare provider during your pregnancy? | primary health center | Count | 112 | 67 |  |
|  |  | Expected Count | 115.3 | 63.7 |  |
|  | Lady Health Visitor | Count | 6 | 0 |  |
|  |  | Expected Count | 3.9 | 2.1 |  |
|  | medical assistant | Count | 5 | 0 |  |
|  |  | Expected Count | 3.2 | 1.8 |  |
|  | Trained midwife | Count | 0 | 1 |  |
|  |  | Expected Count | .6 | .4 |  |
| Total | | Count | 123 | 68 |  |
|  | | Expected Count | 123.0 | 68.0 |  |

| **Chi-Square Tests** | | | |
| --- | --- | --- | --- |
|  | Value | df | Asymptotic Significance (2-sided) |
| Pearson Chi-Square | 8.151^a^ | 3 | .043 |
| Likelihood Ratio | 12.006 | 3 | .007 |
| Linear-by-Linear Association | 1.857 | 1 | .173 |
| N of Valid Cases | 191 |  |  |

| a. 6 cells (75.0%) have expected count less than 5. The minimum expected count is .36. |
| --- |

| **Symmetric Measures** | | | |
| --- | --- | --- | --- |
|  | | Value | Approximate Significance |
| Nominal by Nominal | Contingency Coefficient | .202 | .043 |
| N of Valid Cases | | 191 |  |

**What type of healthcare services were provided?* WHO_recommendation_for_ANC_visits**

| **Crosstab** | | | | | |
| --- | --- | --- | --- | --- | --- |
|  | | | WHO_recommendation_for_ANC_visits | | Total |
|  | | | no | yes |  |
| What type of healthcare services were provided? | private | Count | 104 | 52 | 156 |
|  |  | Expected Count | 97.2 | 58.8 | 156.0 |
|  | public | Count | 38 | 34 | 72 |
|  |  | Expected Count | 44.8 | 27.2 | 72.0 |
| Total | | Count | 142 | 86 | 228 |
|  | | Expected Count | 142.0 | 86.0 | 228.0 |

| **Chi-Square Tests** | | | | | |
| --- | --- | --- | --- | --- | --- |
|  | Value | df | Asymptotic Significance (2-sided) | Exact Sig. (2-sided) | Exact Sig. (1-sided) |
| Pearson Chi-Square | 4.045^a^ | 1 | .044 |  |  |
| Continuity Correction^b^ | 3.476 | 1 | .062 |  |  |
| Likelihood Ratio | 3.996 | 1 | .046 |  |  |
| Fisher's Exact Test |  |  |  | .056 | .032 |
| Linear-by-Linear Association | 4.027 | 1 | .045 |  |  |
| N of Valid Cases | 228 |  |  |  |  |

| a. 0 cells (0.0%) have expected count less than 5. The minimum expected count is 27.16. |
| --- |
| b. Computed only for a 2x2 table |

| **Symmetric Measures** | | | |
| --- | --- | --- | --- |
|  | | Value | Approximate Significance |
| Nominal by Nominal | Contingency Coefficient | .132 | .044 |
| N of Valid Cases | | 228 |  |

**Do you have health insurance? * WHO_recommendation_for_ANC_visits**

| **Crosstab** | | | | | |
| --- | --- | --- | --- | --- | --- |
|  | | | WHO_recommendation_for_ANC_visits | | Total |
|  | | | no | yes |  |
| Do you have health insurance? | no | Count | 138 | 75 | 213 |
|  |  | Expected Count | 140.0 | 73.0 | 213.0 |
|  | yes | Count | 26 | 11 | 37 |
|  |  | Expected Count | 24.3 | 12.7 | 37.0 |
|  | I do not know | Count | 1 | 0 | 1 |
|  |  | Expected Count | .7 | .3 | 1.0 |
| Total | | Count | 165 | 86 | 251 |
|  | | Expected Count | 165.0 | 86.0 | 251.0 |

| **Chi-Square Tests** | | | |
| --- | --- | --- | --- |
|  | Value | df | Asymptotic Significance (2-sided) |
| Pearson Chi-Square | .944^a^ | 2 | .624 |
| Likelihood Ratio | 1.269 | 2 | .530 |
| Linear-by-Linear Association | .706 | 1 | .401 |
| N of Valid Cases | 251 |  |  |

| a. 2 cells (33.3%) have expected count less than 5. The minimum expected count is .34. |
| --- |

| **Symmetric Measures** | | | |
| --- | --- | --- | --- |
|  | | Value | Approximate Significance |
| Nominal by Nominal | Contingency Coefficient | .061 | .624 |
| N of Valid Cases | | 251 |  |

**What means of transportation do you use to reach the maternity healthcare center?* WHO_recommendation_for_ANC_visits**

| **Crosstab** | | | | | |
| --- | --- | --- | --- | --- | --- |
|  | | | WHO_recommendation_for_ANC_visits | |  |
|  | | | no | yes |  |
| What means of transportation do you use to reach the maternity healthcare center? | walking | Count | 72 | 32 |  |
|  |  | Expected Count | 66.3 | 37.7 |  |
|  | public transportation | Count | 57 | 40 |  |
|  |  | Expected Count | 61.8 | 35.2 |  |
|  | private car | Count | 10 | 7 |  |
|  |  | Expected Count | 10.8 | 6.2 |  |
| Total | | Count | 139 | 79 |  |
|  | | Expected Count | 139.0 | 79.0 |  |

| **Chi-Square Tests** | | | |
| --- | --- | --- | --- |
|  | Value | df | Asymptotic Significance (2-sided) |
| Pearson Chi-Square | 2.575^a^ | 2 | .276 |
| Likelihood Ratio | 2.587 | 2 | .274 |
| Linear-by-Linear Association | 2.127 | 1 | .145 |
| N of Valid Cases | 218 |  |  |

| a. 0 cells (0.0%) have expected count less than 5. The minimum expected count is 6.16. |
| --- |

| **Symmetric Measures** | | | |
| --- | --- | --- | --- |
|  | | Value | Approximate Significance |
| Nominal by Nominal | Contingency Coefficient | .108 | .276 |
| N of Valid Cases | | 218 |  |

**Was the circumcision procedure performed on you?* WHO_recommendation_for_ANC_visits**

| **Crosstab** | | | | | |
| --- | --- | --- | --- | --- | --- |
|  | | | WHO_recommendation_for_ANC_visits | | Total |
|  | | | no | yes |  |
| Was the circumcision procedure performed on you? | no | Count | 3 | 1 | 4 |
|  |  | Expected Count | 2.6 | 1.4 | 4.0 |
|  | yes | Count | 161 | 85 | 246 |
|  |  | Expected Count | 161.7 | 84.3 | 246.0 |
|  | I do not know | Count | 1 | 0 | 1 |
|  |  | Expected Count | .7 | .3 | 1.0 |
| Total | | Count | 165 | 86 | 251 |
|  | | Expected Count | 165.0 | 86.0 | 251.0 |

| **Chi-Square Tests** | | | |
| --- | --- | --- | --- |
|  | Value | df | Asymptotic Significance (2-sided) |
| Pearson Chi-Square | .683^a^ | 2 | .711 |
| Likelihood Ratio | 1.009 | 2 | .604 |
| Linear-by-Linear Association | .001 | 1 | .979 |
| N of Valid Cases | 251 |  |  |

| a. 4 cells (66.7%) have expected count less than 5. The minimum expected count is .34. |
| --- |

| **Symmetric Measures** | | | |
| --- | --- | --- | --- |
|  | | Value | Approximate Significance |
| Nominal by Nominal | Contingency Coefficient | .052 | .711 |
| N of Valid Cases | | 251 |  |
