# Supplementary material for: Antenatal Care Service Utilization Among Childbearing Women at El‐Digysab Village, El‐Jazeera State, Sudan, 2023
Source: J Pregnancy. 2026 Mar 27;2026:5565023. doi: 10.1155/jp/5565023 (PMC13140838; doi:10.1155/jp/5565023)
Supplement: Supplementary file 1 — Supporting Information 1 Additional supporting information can be found online in the Supporting Information section. Supporting Information A file containing all the data sheets and analysis outputs generated in the study, including the tests, frequencies, and descriptive statistics. [file JP-2026-5565023-s001.zip › Revised analysis output/Revised copy of ANC vs Complications.docx]

GET

FILE='C:\Users\hp\Documents\elmnagel medical day\SPSS DATA\ANC CLEANED Eyad.sav'.

DATASET NAME DataSet1 WINDOW=FRONT.

CROSSTABS

/TABLES=

- During your last pregnancy, did you experience anemia?
- During your last pregnancy, did you experience cramps
- During your last pregnancy, did you experience thyroid diseases or their complications?
- During your last pregnancy, did you experience preterm labor or incomplete delivery?
- During your last pregnancy, did you experience obstructed or difficult labor?
- During your last pregnancy, did you experience shoulder dystocia?
- During your last pregnancy, did you experience a large baby weighing ≥ 4.5 kg?
- During your last pregnancy, did you experience postpartum hemorrhage?
- During your last pregnancy, did you experience intrauterine growth restriction (IUGR) or halted fetal growth?
- During your last pregnancy, did you experience intrauterine fetal death?
- Did you experience any other complications? If so, what were they?

BY

WHO_recommendation_for_ANC_visits

/FORMAT=AVALUE TABLES

/STATISTICS=CHISQ CC

/CELLS=COUNT EXPECTED

/COUNT ROUND CELL.

**Crosstabs**

| **Notes** | | |
| --- | --- | --- |
| Output Created | | 05-MAY-2023 20:39:49 |
| Comments | |  |
| Input | Data | C:\Users\hp\Documents\elmnagel medical day\SPSS DATA\ANC CLEANED Eyad.sav |
|  | Active Dataset | DataSet1 |
|  | Filter | <none> |
|  | Weight | <none> |
|  | Split File | <none> |
|  | N of Rows in Working Data File | 251 |
| Missing Value Handling | Definition of Missing | User-defined missing values are treated as missing. |
|  | Cases Used | Statistics for each table are based on all the cases with valid data in the specified range(s) for all variables in each table. |
| Syntax | | CROSSTABS  /TABLES=   - During your last pregnancy, did you experience anemia? - During your last pregnancy, did you experience cramps? - During your last pregnancy, did you experience thyroid diseases or their complications? - During your last pregnancy, did you experience preterm labor or incomplete delivery? - During your last pregnancy, did you experience obstructed or difficult labor? - During your last pregnancy, did you experience shoulder dystocia? - During your last pregnancy, did you experience a large baby weighing ≥ 4.5 kg? - During your last pregnancy, did you experience postpartum hemorrhage? - During your last pregnancy, did you experience intrauterine growth restriction (IUGR) or halted fetal growth? - During your last pregnancy, did you experience intrauterine fetal death? - Did you experience any other complications? If so, what were they?   BY  WHO_recommendation_for_ANC_visits  /FORMAT=AVALUE TABLES  /STATISTICS=CHISQ CC  /CELLS=COUNT EXPECTED  /COUNT ROUND CELL. |
| Resources | Processor Time | 00:00:00.03 |
|  | Elapsed Time | 00:00:00.02 |
|  | Dimensions Requested | 2 |
|  | Cells Available | 524245 |

[DataSet1] C:\Users\hp\Documents\elmnagel medical day\SPSS DATA\ANC CLEANED Eyad.sav

| **Warnings** |
| --- |
| The crosstabulation of Did you experience any other complications? If so, what were they?* WHO_recommendation_for_ANC_visits is empty. |

| **Case Processing Summary** | | | | | | |
| --- | --- | --- | --- | --- | --- | --- |
|  | Cases | | | | | |
|  | Valid | | Missing | | Total | |
|  | N | Percent | N | Percent | N | Percent |
| During your last pregnancy, did you experience anemia? * WHO_recommendation_for_ANC_visits | 251 | 100.0% | 0 | 0.0% | 251 | 100.0% |
| During your last pregnancy, did you experience cramps? * WHO_recommendation_for_ANC_visits | 250 | 99.6% | 1 | 0.4% | 251 | 100.0% |
| During your last pregnancy, did you experience thyroid diseases or their complications? * WHO_recommendation_for_ANC_visits | 250 | 99.6% | 1 | 0.4% | 251 | 100.0% |
| During your last pregnancy, did you experience preterm labor or incomplete delivery?* WHO_recommendation_for_ANC_visits | 251 | 100.0% | 0 | 0.0% | 251 | 100.0% |
| During your last pregnancy, did you experience obstructed or difficult labor?* WHO_recommendation_for_ANC_visits | 250 | 99.6% | 1 | 0.4% | 251 | 100.0% |
| During your last pregnancy, did you experience shoulder dystocia?* WHO_recommendation_for_ANC_visits | 250 | 99.6% | 1 | 0.4% | 251 | 100.0% |
| During your last pregnancy, did you experience a large baby weighing ≥ 4.5 kg?* WHO_recommendation_for_ANC_visits | 249 | 99.2% | 2 | 0.8% | 251 | 100.0% |
| During your last pregnancy, did you experience postpartum hemorrhage?* WHO_recommendation_for_ANC_visits | 249 | 99.2% | 2 | 0.8% | 251 | 100.0% |
| During your last pregnancy, did you experience intrauterine growth restriction (IUGR) or halted fetal growth?* WHO_recommendation_for_ANC_visits | 250 | 99.6% | 1 | 0.4% | 251 | 100.0% |
| During your last pregnancy, did you experience intrauterine fetal death?* WHO_recommendation_for_ANC_visits | 250 | 99.6% | 1 | 0.4% | 251 | 100.0% |

**During your last pregnancy, did you experience anemia?**

*** WHO_recommendation_for_ANC_visits**

| **Crosstab** | | | | | |
| --- | --- | --- | --- | --- | --- |
|  | | | WHO_recommendation_for_ANC_visits | | Total |
|  | | | no | yes |  |
| During your last pregnancy, did you experience anemia? | no | Count | 150 | 78 | 228 |
|  |  | Expected Count | 149.9 | 78.1 | 228.0 |
|  | yes | Count | 15 | 8 | 23 |
|  |  | Expected Count | 15.1 | 7.9 | 23.0 |
| Total | | Count | 165 | 86 | 251 |
|  | | Expected Count | 165.0 | 86.0 | 251.0 |

| **Chi-Square Tests** | | | | | |
| --- | --- | --- | --- | --- | --- |
|  | Value | df | Asymptotic Significance (2-sided) | Exact Sig. (2-sided) | Exact Sig. (1-sided) |
| Pearson Chi-Square | .003^a^ | 1 | .956 |  |  |
| Continuity Correction^b^ | .000 | 1 | 1.000 |  |  |
| Likelihood Ratio | .003 | 1 | .956 |  |  |
| Fisher's Exact Test |  |  |  | 1.000 | .562 |
| Linear-by-Linear Association | .003 | 1 | .956 |  |  |
| N of Valid Cases | 251 |  |  |  |  |

| a. 0 cells (0.0%) have expected count less than 5. The minimum expected count is 7.88. |
| --- |
| b. Computed only for a 2x2 table |

| **Symmetric Measures** | | | |
| --- | --- | --- | --- |
|  | | Value | Approximate Significance |
| Nominal by Nominal | Contingency Coefficient | .003 | .956 |
| N of Valid Cases | | 251 |  |

**During your last pregnancy, did you experience cramps?* WHO_recommendation_for_ANC_visits**

| **Crosstab** | | | | | |
| --- | --- | --- | --- | --- | --- |
|  | | | WHO_recommendation_for_ANC_visits | | Total |
|  | | | no | yes |  |
| During your last pregnancy, did you experience cramps? | no | Count | 161 | 82 | 243 |
|  |  | Expected Count | 159.4 | 83.6 | 243.0 |
|  | yes | Count | 3 | 4 | 7 |
|  |  | Expected Count | 4.6 | 2.4 | 7.0 |
| Total | | Count | 164 | 86 | 250 |
|  | | Expected Count | 164.0 | 86.0 | 250.0 |

| **Chi-Square Tests** | | | | | |
| --- | --- | --- | --- | --- | --- |
|  | Value | df | Asymptotic Significance (2-sided) | Exact Sig. (2-sided) | Exact Sig. (1-sided) |
| Pearson Chi-Square | 1.651^a^ | 1 | .199 |  |  |
| Continuity Correction^b^ | .777 | 1 | .378 |  |  |
| Likelihood Ratio | 1.552 | 1 | .213 |  |  |
| Fisher's Exact Test |  |  |  | .237 | .187 |
| Linear-by-Linear Association | 1.644 | 1 | .200 |  |  |
| N of Valid Cases | 250 |  |  |  |  |

| a. 2 cells (50.0%) have expected count less than 5. The minimum expected count is 2.41. |
| --- |
| b. Computed only for a 2x2 table |

| **Symmetric Measures** | | | |
| --- | --- | --- | --- |
|  | | Value | Approximate Significance |
| Nominal by Nominal | Contingency Coefficient | .081 | .199 |
| N of Valid Cases | | 250 |  |

**During your last pregnancy, did you experience thyroid diseases or their complications?* WHO_recommendation_for_ANC_visits**

| **Crosstab** | | | | | |
| --- | --- | --- | --- | --- | --- |
|  | | | WHO_recommendation_for_ANC_visits | | Total |
|  | | | no | yes |  |
| During your last pregnancy, did you experience thyroid diseases or their complications? | no | Count | 158 | 82 | 240 |
|  |  | Expected Count | 158.4 | 81.6 | 240.0 |
|  | yes | Count | 7 | 3 | 10 |
|  |  | Expected Count | 6.6 | 3.4 | 10.0 |
| Total | | Count | 165 | 85 | 250 |
|  | | Expected Count | 165.0 | 85.0 | 250.0 |

| **Chi-Square Tests** | | | | | |
| --- | --- | --- | --- | --- | --- |
|  | Value | df | Asymptotic Significance (2-sided) | Exact Sig. (2-sided) | Exact Sig. (1-sided) |
| Pearson Chi-Square | .074^a^ | 1 | .785 |  |  |
| Continuity Correction^b^ | .000 | 1 | 1.000 |  |  |
| Likelihood Ratio | .076 | 1 | .783 |  |  |
| Fisher's Exact Test |  |  |  | 1.000 | .541 |
| Linear-by-Linear Association | .074 | 1 | .786 |  |  |
| N of Valid Cases | 250 |  |  |  |  |

| a. 1 cells (25.0%) have expected count less than 5. The minimum expected count is 3.40. |
| --- |
| b. Computed only for a 2x2 table |

| **Symmetric Measures** | | | |
| --- | --- | --- | --- |
|  | | Value | Approximate Significance |
| Nominal by Nominal | Contingency Coefficient | .017 | .785 |
| N of Valid Cases | | 250 |  |

**During your last pregnancy, did you experience preterm labor or incomplete delivery?* WHO_recommendation_for_ANC_visits**

| **Crosstab** | | | | | |
| --- | --- | --- | --- | --- | --- |
|  | | | WHO_recommendation_for_ANC_visits | | Total |
|  | | | no | yes |  |
| During your last pregnancy, did you experience preterm labor or incomplete delivery? | no | Count | 150 | 78 | 228 |
|  |  | Expected Count | 149.9 | 78.1 | 228.0 |
|  | yes | Count | 15 | 8 | 23 |
|  |  | Expected Count | 15.1 | 7.9 | 23.0 |
| Total | | Count | 165 | 86 | 251 |
|  | | Expected Count | 165.0 | 86.0 | 251.0 |

| **Chi-Square Tests** | | | | | |
| --- | --- | --- | --- | --- | --- |
|  | Value | df | Asymptotic Significance (2-sided) | Exact Sig. (2-sided) | Exact Sig. (1-sided) |
| Pearson Chi-Square | .003^a^ | 1 | .956 |  |  |
| Continuity Correction^b^ | .000 | 1 | 1.000 |  |  |
| Likelihood Ratio | .003 | 1 | .956 |  |  |
| Fisher's Exact Test |  |  |  | 1.000 | .562 |
| Linear-by-Linear Association | .003 | 1 | .956 |  |  |
| N of Valid Cases | 251 |  |  |  |  |

| a. 0 cells (0.0%) have expected count less than 5. The minimum expected count is 7.88. |
| --- |
| b. Computed only for a 2x2 table |

| **Symmetric Measures** | | | |
| --- | --- | --- | --- |
|  | | Value | Approximate Significance |
| Nominal by Nominal | Contingency Coefficient | .003 | .956 |
| N of Valid Cases | | 251 |  |

**During your last pregnancy, did you experience obstructed or difficult labor?* WHO_recommendation_for_ANC_visits**

| **Crosstab** | | | | | |
| --- | --- | --- | --- | --- | --- |
|  | | | WHO_recommendation_for_ANC_visits | | Total |
|  | | | no | yes |  |
| During your last pregnancy, did you experience obstructed or difficult labor? | no | Count | 129 | 65 | 194 |
|  |  | Expected Count | 127.3 | 66.7 | 194.0 |
|  | yes | Count | 35 | 21 | 56 |
|  |  | Expected Count | 36.7 | 19.3 | 56.0 |
| Total | | Count | 164 | 86 | 250 |
|  | | Expected Count | 164.0 | 86.0 | 250.0 |

| **Chi-Square Tests** | | | | | |
| --- | --- | --- | --- | --- | --- |
|  | Value | df | Asymptotic Significance (2-sided) | Exact Sig. (2-sided) | Exact Sig. (1-sided) |
| Pearson Chi-Square | .307^a^ | 1 | .579 |  |  |
| Continuity Correction^b^ | .156 | 1 | .693 |  |  |
| Likelihood Ratio | .304 | 1 | .581 |  |  |
| Fisher's Exact Test |  |  |  | .633 | .344 |
| Linear-by-Linear Association | .306 | 1 | .580 |  |  |
| N of Valid Cases | 250 |  |  |  |  |

| a. 0 cells (0.0%) have expected count less than 5. The minimum expected count is 19.26. |
| --- |
| b. Computed only for a 2x2 table |

| **Symmetric Measures** | | | |
| --- | --- | --- | --- |
|  | | Value | Approximate Significance |
| Nominal by Nominal | Contingency Coefficient | .035 | .579 |
| N of Valid Cases | | 250 |  |

**During your last pregnancy, did you experience shoulder dystocia?**

*** WHO_recommendation_for_ANC_visits**

| **Crosstab** | | | | | |
| --- | --- | --- | --- | --- | --- |
|  | | | WHO_recommendation_for_ANC_visits | | Total |
|  | | | no | yes |  |
| During your last pregnancy, did you experience shoulder dystocia? | no | Count | 151 | 79 | 230 |
|  |  | Expected Count | 151.8 | 78.2 | 230.0 |
|  | yes | Count | 14 | 6 | 20 |
|  |  | Expected Count | 13.2 | 6.8 | 20.0 |
| Total | | Count | 165 | 85 | 250 |
|  | | Expected Count | 165.0 | 85.0 | 250.0 |

| **Chi-Square Tests** | | | | | |
| --- | --- | --- | --- | --- | --- |
|  | Value | df | Asymptotic Significance (2-sided) | Exact Sig. (2-sided) | Exact Sig. (1-sided) |
| Pearson Chi-Square | .155^a^ | 1 | .694 |  |  |
| Continuity Correction^b^ | .022 | 1 | .883 |  |  |
| Likelihood Ratio | .158 | 1 | .691 |  |  |
| Fisher's Exact Test |  |  |  | .809 | .450 |
| Linear-by-Linear Association | .154 | 1 | .694 |  |  |
| N of Valid Cases | 250 |  |  |  |  |

| a. 0 cells (0.0%) have expected count less than 5. The minimum expected count is 6.80. |
| --- |
| b. Computed only for a 2x2 table |

| **Symmetric Measures** | | | |
| --- | --- | --- | --- |
|  | | Value | Approximate Significance |
| Nominal by Nominal | Contingency Coefficient | .025 | .694 |
| N of Valid Cases | | 250 |  |

**During your last pregnancy, did you experience a large baby weighing ≥ 4.5 kg?* WHO_recommendation_for_ANC_visits**

| **Crosstab** | | | | | |
| --- | --- | --- | --- | --- | --- |
|  | | | WHO_recommendation_for_ANC_visits | | Total |
|  | | | no | yes |  |
| During your last pregnancy, did you experience a large baby weighing ≥ 4.5 kg? | no | Count | 151 | 77 | 228 |
|  |  | Expected Count | 150.2 | 77.8 | 228.0 |
|  | yes | Count | 13 | 8 | 21 |
|  |  | Expected Count | 13.8 | 7.2 | 21.0 |
| Total | | Count | 164 | 85 | 249 |
|  | | Expected Count | 164.0 | 85.0 | 249.0 |

| **Chi-Square Tests** | | | | | |
| --- | --- | --- | --- | --- | --- |
|  | Value | df | Asymptotic Significance (2-sided) | Exact Sig. (2-sided) | Exact Sig. (1-sided) |
| Pearson Chi-Square | .160^a^ | 1 | .689 |  |  |
| Continuity Correction^b^ | .025 | 1 | .873 |  |  |
| Likelihood Ratio | .157 | 1 | .692 |  |  |
| Fisher's Exact Test |  |  |  | .810 | .429 |
| Linear-by-Linear Association | .159 | 1 | .690 |  |  |
| N of Valid Cases | 249 |  |  |  |  |

| a. 0 cells (0.0%) have expected count less than 5. The minimum expected count is 7.17. |
| --- |
| b. Computed only for a 2x2 table |

| **Symmetric Measures** | | | |
| --- | --- | --- | --- |
|  | | Value | Approximate Significance |
| Nominal by Nominal | Contingency Coefficient | .025 | .689 |
| N of Valid Cases | | 249 |  |

**During your last pregnancy, did you experience postpartum hemorrhage?**

*** WHO_recommendation_for_ANC_visits**

| **Crosstab** | | | | | |
| --- | --- | --- | --- | --- | --- |
|  | | | WHO_recommendation_for_ANC_visits | | Total |
|  | | | no | yes |  |
| During your last pregnancy, did you experience postpartum hemorrhage? | no | Count | 156 | 82 | 238 |
|  |  | Expected Count | 156.8 | 81.2 | 238.0 |
|  | yes | Count | 8 | 3 | 11 |
|  |  | Expected Count | 7.2 | 3.8 | 11.0 |
| Total | | Count | 164 | 85 | 249 |
|  | | Expected Count | 164.0 | 85.0 | 249.0 |

| **Chi-Square Tests** | | | | | |
| --- | --- | --- | --- | --- | --- |
|  | Value | df | Asymptotic Significance (2-sided) | Exact Sig. (2-sided) | Exact Sig. (1-sided) |
| Pearson Chi-Square | .241^a^ | 1 | .623 |  |  |
| Continuity Correction^b^ | .028 | 1 | .868 |  |  |
| Likelihood Ratio | .250 | 1 | .617 |  |  |
| Fisher's Exact Test |  |  |  | .754 | .447 |
| Linear-by-Linear Association | .240 | 1 | .624 |  |  |
| N of Valid Cases | 249 |  |  |  |  |

| a. 1 cells (25.0%) have expected count less than 5. The minimum expected count is 3.76. |
| --- |
| b. Computed only for a 2x2 table |

| **Symmetric Measures** | | | |
| --- | --- | --- | --- |
|  | | Value | Approximate Significance |
| Nominal by Nominal | Contingency Coefficient | .031 | .623 |
| N of Valid Cases | | 249 |  |

**During your last pregnancy, did you experience intrauterine growth restriction (IUGR) or halted fetal growth?* WHO_recommendation_for_ANC_visits**

| **Crosstab** | | | | | |
| --- | --- | --- | --- | --- | --- |
|  | | | WHO_recommendation_for_ANC_visits | | Total |
|  | | | no | yes |  |
| During your last pregnancy, did you experience intrauterine growth restriction (IUGR) or halted fetal growth? | no | Count | 162 | 81 | 243 |
|  |  | Expected Count | 160.4 | 82.6 | 243.0 |
|  | yes | Count | 3 | 4 | 7 |
|  |  | Expected Count | 4.6 | 2.4 | 7.0 |
| Total | | Count | 165 | 85 | 250 |
|  | | Expected Count | 165.0 | 85.0 | 250.0 |

| **Chi-Square Tests** | | | | | |
| --- | --- | --- | --- | --- | --- |
|  | Value | df | Asymptotic Significance (2-sided) | Exact Sig. (2-sided) | Exact Sig. (1-sided) |
| Pearson Chi-Square | 1.719^a^ | 1 | .190 |  |  |
| Continuity Correction^b^ | .822 | 1 | .365 |  |  |
| Likelihood Ratio | 1.611 | 1 | .204 |  |  |
| Fisher's Exact Test |  |  |  | .233 | .181 |
| Linear-by-Linear Association | 1.712 | 1 | .191 |  |  |
| N of Valid Cases | 250 |  |  |  |  |

| a. 2 cells (50.0%) have expected count less than 5. The minimum expected count is 2.38. |
| --- |
| b. Computed only for a 2x2 table |

| **Symmetric Measures** | | | |
| --- | --- | --- | --- |
|  | | Value | Approximate Significance |
| Nominal by Nominal | Contingency Coefficient | .083 | .190 |
| N of Valid Cases | | 250 |  |

**During your last pregnancy, did you experience intrauterine fetal death?* WHO_recommendation_for_ANC_visits**

| **Crosstab** | | | | | |
| --- | --- | --- | --- | --- | --- |
|  | | | WHO_recommendation_for_ANC_visits | | Total |
|  | | | no | yes |  |
| During your last pregnancy, did you experience intrauterine fetal death? | no | Count | 153 | 79 | 232 |
|  |  | Expected Count | 153.1 | 78.9 | 232.0 |
|  | yes | Count | 12 | 6 | 18 |
|  |  | Expected Count | 11.9 | 6.1 | 18.0 |
| Total | | Count | 165 | 85 | 250 |
|  | | Expected Count | 165.0 | 85.0 | 250.0 |

| **Chi-Square Tests** | | | | | |
| --- | --- | --- | --- | --- | --- |
|  | Value | df | Asymptotic Significance (2-sided) | Exact Sig. (2-sided) | Exact Sig. (1-sided) |
| Pearson Chi-Square | .004^a^ | 1 | .951 |  |  |
| Continuity Correction^b^ | .000 | 1 | 1.000 |  |  |
| Likelihood Ratio | .004 | 1 | .951 |  |  |
| Fisher's Exact Test |  |  |  | 1.000 | .587 |
| Linear-by-Linear Association | .004 | 1 | .951 |  |  |
| N of Valid Cases | 250 |  |  |  |  |

| a. 0 cells (0.0%) have expected count less than 5. The minimum expected count is 6.12. |
| --- |
| b. Computed only for a 2x2 table |

| **Symmetric Measures** | | | |
| --- | --- | --- | --- |
|  | | Value | Approximate Significance |
| Nominal by Nominal | Contingency Coefficient | .004 | .951 |
| N of Valid Cases | | 250 |  |
