# Supplementary material for: Antenatal Care Service Utilization Among Childbearing Women at El‐Digysab Village, El‐Jazeera State, Sudan, 2023
Source: J Pregnancy. 2026 Mar 27;2026:5565023. doi: 10.1155/jp/5565023 (PMC13140838; doi:10.1155/jp/5565023)
Supplement: Supplementary file 1 — Supporting Information 1 Additional supporting information can be found online in the Supporting Information section. Supporting Information A file containing all the data sheets and analysis outputs generated in the study, including the tests, frequencies, and descriptive statistics. [file JP-2026-5565023-s001.zip › Revised analysis output/Revised copy of driver and barriers vs ANC chi.docx]

CROSSTABS

/TABLES=

- Do you have health insurance?
- ‬What mode of transportation do you use to reach the maternity healthcare center?
- ‬A_ If the answer is "other," what is it?
- ‏‫ How does pregnancy follow-up affect the well-being of the mother and her child?
- Do you often forget your appointment dates?
- ‬Can you obtain the information you need about pregnancy from family or friends?
- Are maternity healthcare appointments usually too short and rushed?
- Are the working hours of the maternity healthcare center convenient for you?
- Are there long waiting periods at the maternity healthcare center?
- ‏‫Do you have trust in your healthcare provider
- ‬Do you think that the people who provide pregnancy care understand your health concerns?
- Do you have someone to take care of your children while you visit the maternity healthcare center?
- Do you have work commitments that take priority over attending maternity healthcare appointments?
- Does your husband have a negative view of maternity care services?
- Does your mother have a negative view of maternity care services?
- ‏‫Do your peers have a negative view of maternity care services
- Does visiting the maternity healthcare center take up a significant amount of your time?
- Does attending the maternity healthcare center require significant financial expenses?
- Is attending the maternity healthcare center physically exhausting for you?

BY WHO_recommendation_for_ANC_visits

/FORMAT=AVALUE TABLES

/STATISTICS=CHISQ CC

/CELLS=COUNT EXPECTED

/COUNT ROUND CELL.

**Crosstabs**

| **Notes** | | |
| --- | --- | --- |
| Output Created | | 05-MAY-2023 21:21:33 |
| Comments | |  |
| Input | Data | C:\Users\hp\Documents\elmnagel medical day\SPSS DATA\ANC CLEANED Eyad.sav |
|  | Active Dataset | DataSet1 |
|  | Filter | <none> |
|  | Weight | <none> |
|  | Split File | <none> |
|  | N of Rows in Working Data File | 251 |
| Missing Value Handling | Definition of Missing | User-defined missing values are treated as missing. |
|  | Cases Used | Statistics for each table are based on all the cases with valid data in the specified range(s) for all variables in each table. |
| Syntax | | CROSSTABS  /TABLES=   - Do you have health insurance? - What mode of transportation do you use to reach the maternity healthcare center? - .A_ If the answer is "other," what is it? - How does pregnancy follow-up affect the well-being of the mother and her child? - Do you often forget your appointment dates? - Can you obtain the information you need about pregnancy from family or friends? - Are maternity healthcare appointments usually too short and rushed? - Are the working hours of the maternity healthcare center convenient for you? - Are there long waiting periods at the maternity healthcare center? - Do you have trust in your healthcare provider? - Do you think that the people who provide pregnancy care understand your health concerns? - Do you have someone to take care of your children while you visit the maternity healthcare center? - Do you have work commitments that take priority over attending maternity healthcare appointments? - Does your husband have a negative view of maternity care services? - Does your mother have a negative view of maternity care services? - Do your peers have a negative view of maternity care services? - Does visiting the maternity healthcare center take up a significant amount of your time? - Does attending the maternity healthcare center require significant financial expenses - Is attending the maternity healthcare center physically exhausting for you? BY WHO_recommendation_for_ANC_visits   /FORMAT=AVALUE TABLES  /STATISTICS=CHISQ CC  /CELLS=COUNT EXPECTED  /COUNT ROUND CELL. |
| Resources | Processor Time | 00:00:00.06 |
|  | Elapsed Time | 00:00:00.05 |
|  | Dimensions Requested | 2 |
|  | Cells Available | 524245 |

| **Warnings** |
| --- |
| The crosstabulation of If the answer is "Other," what is it?  _A * WHO_recommendation_for_ANC_visits is empty. |

| **Case Processing Summary** | | | | | | |
| --- | --- | --- | --- | --- | --- | --- |
|  | Cases | | | | | |
|  | Valid | | Missing | | Total | |
|  | N | Percent | N | Percent | N | Percent |
| Do you have health insurance? * WHO_recommendation_for_ANC_visits | 251 | 100.0% | 0 | 0.0% | 251 | 100.0% |
| What mode of transportation do you use to reach the maternity healthcare center?* WHO_recommendation_for_ANC_visits | 218 | 86.9% | 33 | 13.1% | 251 | 100.0% |
| How does pregnancy follow-up affect the well-being of the mother and her child? * WHO_recommendation_for_ANC_visits | 250 | 99.6% | 1 | 0.4% | 251 | 100.0% |
| Do you often forget your appointment dates? WHO_recommendation_for_ANC_visits | 250 | 99.6% | 1 | 0.4% | 251 | 100.0% |
| Can you obtain the information you need about pregnancy from family or friends? * WHO_recommendation_for_ANC_visits | 251 | 100.0% | 0 | 0.0% | 251 | 100.0% |
| Are maternity healthcare appointments usually too short and rushed?* WHO_recommendation_for_ANC_visits | 251 | 100.0% | 0 | 0.0% | 251 | 100.0% |
| Are the working hours of the maternity healthcare center convenient for you? * WHO_recommendation_for_ANC_visits | 249 | 99.2% | 2 | 0.8% | 251 | 100.0% |
| Are there long waiting periods at the maternity healthcare center?* WHO_recommendation_for_ANC_visits | 250 | 99.6% | 1 | 0.4% | 251 | 100.0% |
| Do you have trust in your healthcare provider? * WHO_recommendation_for_ANC_visits | 249 | 99.2% | 2 | 0.8% | 251 | 100.0% |
| Do you think that the people who provide pregnancy care understand your health concerns? * WHO_recommendation_for_ANC_visits | 249 | 99.2% | 2 | 0.8% | 251 | 100.0% |
| Do you have someone to take care of your children while you visit the maternity healthcare center? * WHO_recommendation_for_ANC_visits | 249 | 99.2% | 2 | 0.8% | 251 | 100.0% |
| Do you have work commitments that take priority over attending maternity healthcare appointments? * WHO_recommendation_for_ANC_visits | 249 | 99.2% | 2 | 0.8% | 251 | 100.0% |
| Does your husband have a negative view of maternity care services?* WHO_recommendation_for_ANC_visits | 251 | 100.0% | 0 | 0.0% | 251 | 100.0% |
| Does your mother have a negative view of maternity care services? * WHO_recommendation_for_ANC_visits | 250 | 99.6% | 1 | 0.4% | 251 | 100.0% |
| Do your peers have a negative view of maternity care services? * WHO_recommendation_for_ANC_visits | 250 | 99.6% | 1 | 0.4% | 251 | 100.0% |
| Does visiting the maternity healthcare center take up a significant amount of your time? * WHO_recommendation_for_ANC_visits | 250 | 99.6% | 1 | 0.4% | 251 | 100.0% |
| Does attending the maternity healthcare center require significant financial expenses? * WHO_recommendation_for_ANC_visits | 250 | 99.6% | 1 | 0.4% | 251 | 100.0% |
| Is attending the maternity healthcare center physically exhausting for you? * WHO_recommendation_for_ANC_visits | 250 | 99.6% | 1 | 0.4% | 251 | 100.0% |

**Do you have health insurance? * WHO_recommendation_for_ANC_visits**

| **Crosstab** | | | | | |
| --- | --- | --- | --- | --- | --- |
|  | | | WHO_recommendation_for_ANC_visits | | Total |
|  | | | no | yes |  |
| Do you have health insurance? | no | Count | 138 | 75 | 213 |
|  |  | Expected Count | 140.0 | 73.0 | 213.0 |
|  | yes | Count | 26 | 11 | 37 |
|  |  | Expected Count | 24.3 | 12.7 | 37.0 |
|  | I do not know | Count | 1 | 0 | 1 |
|  |  | Expected Count | .7 | .3 | 1.0 |
| Total | | Count | 165 | 86 | 251 |
|  | | Expected Count | 165.0 | 86.0 | 251.0 |

| **Chi-Square Tests** | | | |
| --- | --- | --- | --- |
|  | Value | df | Asymptotic Significance (2-sided) |
| Pearson Chi-Square | .944^a^ | 2 | .624 |
| Likelihood Ratio | 1.269 | 2 | .530 |
| Linear-by-Linear Association | .706 | 1 | .401 |
| N of Valid Cases | 251 |  |  |

| a. 2 cells (33.3%) have expected count less than 5. The minimum expected count is .34. |
| --- |

| **Symmetric Measures** | | | |
| --- | --- | --- | --- |
|  | | Value | Approximate Significance |
| Nominal by Nominal | Contingency Coefficient | .061 | .624 |
| N of Valid Cases | | 251 |  |

**What mode of transportation do you use to reach the maternity healthcare center? * WHO_recommendation_for_ANC_visits**

| **Crosstab** | | | | | |
| --- | --- | --- | --- | --- | --- |
|  | | | WHO_recommendation_for_ANC_visits | |  |
|  | | | no | yes |  |
| What mode of transportation do you use to reach the maternity healthcare center? | walking | Count | 72 | 32 |  |
|  |  | Expected Count | 66.3 | 37.7 |  |
|  | public transportation | Count | 57 | 40 |  |
|  |  | Expected Count | 61.8 | 35.2 |  |
|  | private car | Count | 10 | 7 |  |
|  |  | Expected Count | 10.8 | 6.2 |  |
| Total | | Count | 139 | 79 |  |
|  | | Expected Count | 139.0 | 79.0 |  |

| **Chi-Square Tests** | | | |
| --- | --- | --- | --- |
|  | Value | df | Asymptotic Significance (2-sided) |
| Pearson Chi-Square | 2.575^a^ | 2 | .276 |
| Likelihood Ratio | 2.587 | 2 | .274 |
| Linear-by-Linear Association | 2.127 | 1 | .145 |
| N of Valid Cases | 218 |  |  |

| a. 0 cells (0.0%) have expected count less than 5. The minimum expected count is 6.16. |
| --- |

| **Symmetric Measures** | | | |
| --- | --- | --- | --- |
|  | | Value | Approximate Significance |
| Nominal by Nominal | Contingency Coefficient | .108 | .276 |
| N of Valid Cases | | 218 |  |

**How does pregnancy follow-up affect the well-being of the mother and her child?* WHO_recommendation_for_ANC_visits**

| **Crosstab** | | | | | |
| --- | --- | --- | --- | --- | --- |
|  | | | WHO_recommendation_for_ANC_visits | | Total |
|  | | | no | yes |  |
| How does maternity care impact the health of the mother and baby? | I do not know | Count | 8 | 1 | 9 |
|  |  | Expected Count | 5.9 | 3.1 | 9.0 |
|  | positivly affect | Count | 152 | 84 | 236 |
|  |  | Expected Count | 154.8 | 81.2 | 236.0 |
|  | does not affect | Count | 3 | 0 | 3 |
|  |  | Expected Count | 2.0 | 1.0 | 3.0 |
|  | negetivly affect | Count | 1 | 1 | 2 |
|  |  | Expected Count | 1.3 | .7 | 2.0 |
| Total | | Count | 164 | 86 | 250 |
|  | | Expected Count | 164.0 | 86.0 | 250.0 |

| **Chi-Square Tests** | | | |
| --- | --- | --- | --- |
|  | Value | df | Asymptotic Significance (2-sided) |
| Pearson Chi-Square | 4.101^a^ | 3 | .251 |
| Likelihood Ratio | 5.483 | 3 | .140 |
| Linear-by-Linear Association | .629 | 1 | .428 |
| N of Valid Cases | 250 |  |  |

| a. 5 cells (62.5%) have expected count less than 5. The minimum expected count is .69. |
| --- |

| **Symmetric Measures** | | | |
| --- | --- | --- | --- |
|  | | Value | Approximate Significance |
| Nominal by Nominal | Contingency Coefficient | .127 | .251 |
| N of Valid Cases | | 250 |  |

**Do you often forget your appointment dates? * WHO_recommendation_for_ANC_visits**

| **Crosstab** | | | | | |
| --- | --- | --- | --- | --- | --- |
|  | | | WHO_recommendation_for_ANC_visits | |  |
|  | | | no | yes |  |
| هDo you often forget your appointment dates? | I do not know‎/ i do not remember | Count | 5 | 1 |  |
|  |  | Expected Count | 3.9 | 2.1 |  |
|  | YES | Count | 108 | 67 |  |
|  |  | Expected Count | 114.8 | 60.2 |  |
|  | neutral | Count | 39 | 13 |  |
|  |  | Expected Count | 34.1 | 17.9 |  |
|  | NO | Count | 8 | 3 |  |
|  |  | Expected Count | 7.2 | 3.8 |  |
|  | I do not know‎ there is a fixed visits schedule | Count | 4 | 2 |  |
|  |  | Expected Count | 3.9 | 2.1 |  |
| Total | | Count | 164 | 86 |  |
|  | | Expected Count | 164.0 | 86.0 |  |

| **Chi-Square Tests** | | | |
| --- | --- | --- | --- |
|  | Value | df | Asymptotic Significance (2-sided) |
| Pearson Chi-Square | 4.294^a^ | 4 | .368 |
| Likelihood Ratio | 4.502 | 4 | .342 |
| Linear-by-Linear Association | 1.089 | 1 | .297 |
| N of Valid Cases | 250 |  |  |

| a. 5 cells (50.0%) have expected count less than 5. The minimum expected count is 2.06. |
| --- |

| **Symmetric Measures** | | | |
| --- | --- | --- | --- |
|  | | Value | Approximate Significance |
| Nominal by Nominal | Contingency Coefficient | .130 | .368 |
| N of Valid Cases | | 250 |  |

**Can you obtain the information you need about pregnancy from family or friends? * WHO_recommendation_for_ANC_visits**

| **Crosstab** | | | | | |
| --- | --- | --- | --- | --- | --- |
|  | | | WHO_recommendation_for_ANC_visits | | Total |
|  | | | no | yes |  |
| Can you obtain the information you need about pregnancy from family or friends? | I do not know‎ | Count | 1 | 1 | 2 |
|  |  | Expected Count | 1.3 | .7 | 2.0 |
|  | YES | Count | 72 | 38 | 110 |
|  |  | Expected Count | 72.3 | 37.7 | 110.0 |
|  | neutral | Count | 75 | 38 | 113 |
|  |  | Expected Count | 74.3 | 38.7 | 113.0 |
|  | NO | Count | 17 | 9 | 26 |
|  |  | Expected Count | 17.1 | 8.9 | 26.0 |
| Total | | Count | 165 | 86 | 251 |
|  | | Expected Count | 165.0 | 86.0 | 251.0 |

| **Chi-Square Tests** | | | |
| --- | --- | --- | --- |
|  | Value | df | Asymptotic Significance (2-sided) |
| Pearson Chi-Square | .245^a^ | 3 | .970 |
| Likelihood Ratio | .234 | 3 | .972 |
| Linear-by-Linear Association | .028 | 1 | .867 |
| N of Valid Cases | 251 |  |  |

| a. 2 cells (25.0%) have expected count less than 5. The minimum expected count is .69. |
| --- |

| **Symmetric Measures** | | | |
| --- | --- | --- | --- |
|  | | Value | Approximate Significance |
| Nominal by Nominal | Contingency Coefficient | .031 | .970 |
| N of Valid Cases | | 251 |  |

**Are maternity healthcare appointments usually too short and rushed? * WHO_recommendation_for_ANC_visits**

| **Crosstab** | | | | | |
| --- | --- | --- | --- | --- | --- |
|  | | | WHO_recommendation_for_ANC_visits | | Total |
|  | | | no | yes |  |
| Are maternity healthcare appointments usually too short and rushed? | I do not know‎ | Count | 6 | 0 | 6 |
|  |  | Expected Count | 3.9 | 2.1 | 6.0 |
|  | YES | Count | 59 | 31 | 90 |
|  |  | Expected Count | 59.2 | 30.8 | 90.0 |
|  | neutral | Count | 73 | 33 | 106 |
|  |  | Expected Count | 69.7 | 36.3 | 106.0 |
|  | NO | Count | 27 | 22 | 49 |
|  |  | Expected Count | 32.2 | 16.8 | 49.0 |
| Total | | Count | 165 | 86 | 251 |
|  | | Expected Count | 165.0 | 86.0 | 251.0 |

| **Chi-Square Tests** | | | |
| --- | --- | --- | --- |
|  | Value | df | Asymptotic Significance (2-sided) |
| Pearson Chi-Square | 6.050^a^ | 3 | .109 |
| Likelihood Ratio | 7.869 | 3 | .049 |
| Linear-by-Linear Association | 2.444 | 1 | .118 |
| N of Valid Cases | 251 |  |  |

| a. 2 cells (25.0%) have expected count less than 5. The minimum expected count is 2.06. |
| --- |

| **Symmetric Measures** | | | |
| --- | --- | --- | --- |
|  | | Value | Approximate Significance |
| Nominal by Nominal | Contingency Coefficient | .153 | .109 |
| N of Valid Cases | | 251 |  |

**Are the working hours of the maternity healthcare center convenient for you? * WHO_recommendation_for_ANC_visits**

| **Crosstab** | | | | | |
| --- | --- | --- | --- | --- | --- |
|  | | | WHO_recommendation_for_ANC_visits | | Total |
|  | | | no | yes |  |
| Are the working hours of the maternity healthcare center convenient for you? | I do not know‎ | Count | 9 | 0 | 9 |
|  |  | Expected Count | 5.9 | 3.1 | 9.0 |
|  | YES | Count | 14 | 7 | 21 |
|  |  | Expected Count | 13.7 | 7.3 | 21.0 |
|  | neutral | Count | 133 | 75 | 208 |
|  |  | Expected Count | 136.2 | 71.8 | 208.0 |
|  | NO | Count | 7 | 4 | 11 |
|  |  | Expected Count | 7.2 | 3.8 | 11.0 |
| Total | | Count | 163 | 86 | 249 |
|  | | Expected Count | 163.0 | 86.0 | 249.0 |

| **Chi-Square Tests** | | | |
| --- | --- | --- | --- |
|  | Value | df | Asymptotic Significance (2-sided) |
| Pearson Chi-Square | 4.991^a^ | 3 | .172 |
| Likelihood Ratio | 7.867 | 3 | .049 |
| Linear-by-Linear Association | 3.023 | 1 | .082 |
| N of Valid Cases | 249 |  |  |

| a. 2 cells (25.0%) have expected count less than 5. The minimum expected count is 3.11. |
| --- |

| **Symmetric Measures** | | | |
| --- | --- | --- | --- |
|  | | Value | Approximate Significance |
| Nominal by Nominal | Contingency Coefficient | .140 | .172 |
| N of Valid Cases | | 249 |  |

**Are there long waiting periods at the maternity healthcare center? * WHO_recommendation_for_ANC_visits**

| **Crosstab** | | | | | |
| --- | --- | --- | --- | --- | --- |
|  | | | WHO_recommendation_for_ANC_visits | | Total |
|  | | | no | yes |  |
| Are there long waiting periods at the maternity healthcare center? | I do not know‎ | Count | 7 | 0 | 7 |
|  |  | Expected Count | 4.6 | 2.4 | 7.0 |
|  | YES | Count | 36 | 24 | 60 |
|  |  | Expected Count | 39.4 | 20.6 | 60.0 |
|  | neutral | Count | 96 | 48 | 144 |
|  |  | Expected Count | 94.5 | 49.5 | 144.0 |
|  | NO | Count | 25 | 14 | 39 |
|  |  | Expected Count | 25.6 | 13.4 | 39.0 |
| Total | | Count | 164 | 86 | 250 |
|  | | Expected Count | 164.0 | 86.0 | 250.0 |

| **Chi-Square Tests** | | | |
| --- | --- | --- | --- |
|  | Value | df | Asymptotic Significance (2-sided) |
| Pearson Chi-Square | 4.616^a^ | 3 | .202 |
| Likelihood Ratio | 6.829 | 3 | .078 |
| Linear-by-Linear Association | .150 | 1 | .698 |
| N of Valid Cases | 250 |  |  |

| a. 2 cells (25.0%) have expected count less than 5. The minimum expected count is 2.41. |
| --- |

| **Symmetric Measures** | | | |
| --- | --- | --- | --- |
|  | | Value | Approximate Significance |
| Nominal by Nominal | Contingency Coefficient | .135 | .202 |
| N of Valid Cases | | 250 |  |

**Do you have trust in your healthcare provider? * WHO_recommendation_for_ANC_visits**

| **Crosstab** | | | | | |
| --- | --- | --- | --- | --- | --- |
|  | | | WHO_recommendation_for_ANC_visits | | Total |
|  | | | no | yes |  |
| Do you have trust in your healthcare provider? | I do not know‎ | Count | 6 | 0 | 6 |
|  |  | Expected Count | 4.0 | 2.0 | 6.0 |
|  | YES | Count | 7 | 0 | 7 |
|  |  | Expected Count | 4.6 | 2.4 | 7.0 |
|  | neutral | Count | 145 | 84 | 229 |
|  |  | Expected Count | 151.7 | 77.3 | 229.0 |
|  | NO | Count | 7 | 0 | 7 |
|  |  | Expected Count | 4.6 | 2.4 | 7.0 |
| Total | | Count | 165 | 84 | 249 |
|  | | Expected Count | 165.0 | 84.0 | 249.0 |

| **Chi-Square Tests** | | | |
| --- | --- | --- | --- |
|  | Value | df | Asymptotic Significance (2-sided) |
| Pearson Chi-Square | 11.071^a^ | 3 | .011 |
| Likelihood Ratio | 17.338 | 3 | .001 |
| Linear-by-Linear Association | 1.951 | 1 | .162 |
| N of Valid Cases | 249 |  |  |

| a. 6 cells (75.0%) have expected count less than 5. The minimum expected count is 2.02. |
| --- |

| **Symmetric Measures** | | | |
| --- | --- | --- | --- |
|  | | Value | Approximate Significance |
| Nominal by Nominal | Contingency Coefficient | .206 | .011 |
| N of Valid Cases | | 249 |  |

**Do you think that the people who provide pregnancy care understand your health concerns? * WHO_recommendation_for_ANC_visits**

| **Crosstab** | | | | | |
| --- | --- | --- | --- | --- | --- |
|  | | | WHO_recommendation_for_ANC_visits | | Total |
|  | | | no | yes |  |
| Do you think that the people who provide pregnancy care understand your health concerns? | I do not know‎ | Count | 7 | 0 | 7 |
|  |  | Expected Count | 4.6 | 2.4 | 7.0 |
|  | YES | Count | 5 | 1 | 6 |
|  |  | Expected Count | 3.9 | 2.1 | 6.0 |
|  | neutral | Count | 146 | 81 | 227 |
|  |  | Expected Count | 148.6 | 78.4 | 227.0 |
|  | NO | Count | 5 | 4 | 9 |
|  |  | Expected Count | 5.9 | 3.1 | 9.0 |
| Total | | Count | 163 | 86 | 249 |
|  | | Expected Count | 163.0 | 86.0 | 249.0 |

| **Chi-Square Tests** | | | |
| --- | --- | --- | --- |
|  | Value | df | Asymptotic Significance (2-sided) |
| Pearson Chi-Square | 5.063^a^ | 3 | .167 |
| Likelihood Ratio | 7.396 | 3 | .060 |
| Linear-by-Linear Association | 4.790 | 1 | .029 |
| N of Valid Cases | 249 |  |  |

| a. 5 cells (62.5%) have expected count less than 5. The minimum expected count is 2.07. |
| --- |

| **Symmetric Measures** | | | |
| --- | --- | --- | --- |
|  | | Value | Approximate Significance |
| Nominal by Nominal | Contingency Coefficient | .141 | .167 |
| N of Valid Cases | | 249 |  |

**Do you have someone to take care of your children while you visit the maternity healthcare center? * WHO_recommendation_for_ANC_visits**

| **Crosstab** | | | | | |
| --- | --- | --- | --- | --- | --- |
|  | | | WHO_recommendation_for_ANC_visits | | Total |
|  | | | no | yes |  |
| Do you have someone to take care of your children while you visit the maternity healthcare center? | I do not know‎ | Count | 11 | 9 | 20 |
|  |  | Expected Count | 13.1 | 6.9 | 20.0 |
|  | YES | Count | 7 | 4 | 11 |
|  |  | Expected Count | 7.2 | 3.8 | 11.0 |
|  | neutral | Count | 140 | 71 | 211 |
|  |  | Expected Count | 138.1 | 72.9 | 211.0 |
|  | NO | Count | 5 | 2 | 7 |
|  |  | Expected Count | 4.6 | 2.4 | 7.0 |
| Total | | Count | 163 | 86 | 249 |
|  | | Expected Count | 163.0 | 86.0 | 249.0 |

| **Chi-Square Tests** | | | |
| --- | --- | --- | --- |
|  | Value | df | Asymptotic Significance (2-sided) |
| Pearson Chi-Square | 1.168^a^ | 3 | .761 |
| Likelihood Ratio | 1.136 | 3 | .768 |
| Linear-by-Linear Association | 1.126 | 1 | .289 |
| N of Valid Cases | 249 |  |  |

| a. 3 cells (37.5%) have expected count less than 5. The minimum expected count is 2.42. |
| --- |

| **Symmetric Measures** | | | |
| --- | --- | --- | --- |
|  | | Value | Approximate Significance |
| Nominal by Nominal | Contingency Coefficient | .068 | .761 |
| N of Valid Cases | | 249 |  |

**Do you have work commitments that take priority over attending maternity healthcare appointments? * WHO_recommendation_for_ANC_visits**

| **Crosstab** | | | | | |
| --- | --- | --- | --- | --- | --- |
|  | | | WHO_recommendation_for_ANC_visits | | Total |
|  | | | no | yes |  |
| Do you have work commitments that take priority over attending maternity healthcare appointments? | I do not know‎ | Count | 4 | 0 | 4 |
|  |  | Expected Count | 2.6 | 1.4 | 4.0 |
|  | YES | Count | 124 | 74 | 198 |
|  |  | Expected Count | 129.6 | 68.4 | 198.0 |
|  | neutral | Count | 27 | 10 | 37 |
|  |  | Expected Count | 24.2 | 12.8 | 37.0 |
|  | NO | Count | 8 | 2 | 10 |
|  |  | Expected Count | 6.5 | 3.5 | 10.0 |
| Total | | Count | 163 | 86 | 249 |
|  | | Expected Count | 163.0 | 86.0 | 249.0 |

| **Chi-Square Tests** | | | |
| --- | --- | --- | --- |
|  | Value | df | Asymptotic Significance (2-sided) |
| Pearson Chi-Square | 4.673^a^ | 3 | .197 |
| Likelihood Ratio | 6.070 | 3 | .108 |
| Linear-by-Linear Association | 1.171 | 1 | .279 |
| N of Valid Cases | 249 |  |  |

| a. 3 cells (37.5%) have expected count less than 5. The minimum expected count is 1.38. |
| --- |

| **Symmetric Measures** | | | |
| --- | --- | --- | --- |
|  | | Value | Approximate Significance |
| Nominal by Nominal | Contingency Coefficient | .136 | .197 |
| N of Valid Cases | | 249 |  |

**Does your husband have a negative view of maternity care services? * WHO_recommendation_for_ANC_visits**

| **Crosstab** | | | | | |
| --- | --- | --- | --- | --- | --- |
|  | | | WHO_recommendation_for_ANC_visits | | Total |
|  | | | no | yes |  |
| Does your husband have a negative view of maternity care services? | I do not know‎ | Count | 1 | 0 | 1 |
|  |  | Expected Count | .7 | .3 | 1.0 |
|  | YES | Count | 145 | 80 | 225 |
|  |  | Expected Count | 147.9 | 77.1 | 225.0 |
|  | neutral | Count | 15 | 6 | 21 |
|  |  | Expected Count | 13.8 | 7.2 | 21.0 |
|  | NO | Count | 4 | 0 | 4 |
|  |  | Expected Count | 2.6 | 1.4 | 4.0 |
| Total | | Count | 165 | 86 | 251 |
|  | | Expected Count | 165.0 | 86.0 | 251.0 |

| **Chi-Square Tests** | | | |
| --- | --- | --- | --- |
|  | Value | df | Asymptotic Significance (2-sided) |
| Pearson Chi-Square | 3.075^a^ | 3 | .380 |
| Likelihood Ratio | 4.672 | 3 | .197 |
| Linear-by-Linear Association | 1.637 | 1 | .201 |
| N of Valid Cases | 251 |  |  |

| a. 4 cells (50.0%) have expected count less than 5. The minimum expected count is .34. |
| --- |

| **Symmetric Measures** | | | |
| --- | --- | --- | --- |
|  | | Value | Approximate Significance |
| Nominal by Nominal | Contingency Coefficient | .110 | .380 |
| N of Valid Cases | | 251 |  |

**Does your mother have a negative view of maternity care services? * WHO_recommendation_for_ANC_visits**

| **Crosstab** | | | | | |
| --- | --- | --- | --- | --- | --- |
|  | | | WHO_recommendation_for_ANC_visits | | Total |
|  | | | no | yes |  |
| Does your mother have a negative view of maternity care services? | I do not know‎ | Count | 2 | 2 | 4 |
|  |  | Expected Count | 2.6 | 1.4 | 4.0 |
|  | YES | Count | 145 | 81 | 226 |
|  |  | Expected Count | 148.3 | 77.7 | 226.0 |
|  | neutral | Count | 14 | 3 | 17 |
|  |  | Expected Count | 11.2 | 5.8 | 17.0 |
|  | NO | Count | 3 | 0 | 3 |
|  |  | Expected Count | 2.0 | 1.0 | 3.0 |
| Total | | Count | 164 | 86 | 250 |
|  | | Expected Count | 164.0 | 86.0 | 250.0 |

| **Chi-Square Tests** | | | |
| --- | --- | --- | --- |
|  | Value | df | Asymptotic Significance (2-sided) |
| Pearson Chi-Square | 4.327^a^ | 3 | .228 |
| Likelihood Ratio | 5.509 | 3 | .138 |
| Linear-by-Linear Association | 4.287 | 1 | .038 |
| N of Valid Cases | 250 |  |  |

| a. 4 cells (50.0%) have expected count less than 5. The minimum expected count is 1.03. |
| --- |

| **Symmetric Measures** | | | |
| --- | --- | --- | --- |
|  | | Value | Approximate Significance |
| Nominal by Nominal | Contingency Coefficient | .130 | .228 |
| N of Valid Cases | | 250 |  |

**Do your peers have a negative view of maternity care services? * WHO_recommendation_for_ANC_visits**

| **Crosstab** | | | | | |
| --- | --- | --- | --- | --- | --- |
|  | | | WHO_recommendation_for_ANC_visits | | Total |
|  | | | no | yes |  |
| Do your peers have a negative view of maternity care services? | I do not know‎ | Count | 1 | 0 | 1 |
|  |  | Expected Count | .7 | .3 | 1.0 |
|  | YES | Count | 139 | 74 | 213 |
|  |  | Expected Count | 139.7 | 73.3 | 213.0 |
|  | neutral | Count | 21 | 11 | 32 |
|  |  | Expected Count | 21.0 | 11.0 | 32.0 |
|  | NO | Count | 3 | 1 | 4 |
|  |  | Expected Count | 2.6 | 1.4 | 4.0 |
| Total | | Count | 164 | 86 | 250 |
|  | | Expected Count | 164.0 | 86.0 | 250.0 |

| **Chi-Square Tests** | | | |
| --- | --- | --- | --- |
|  | Value | df | Asymptotic Significance (2-sided) |
| Pearson Chi-Square | .692^a^ | 3 | .875 |
| Likelihood Ratio | 1.019 | 3 | .797 |
| Linear-by-Linear Association | .018 | 1 | .894 |
| N of Valid Cases | 250 |  |  |

| a. 4 cells (50.0%) have expected count less than 5. The minimum expected count is .34. |
| --- |

| **Symmetric Measures** | | | |
| --- | --- | --- | --- |
|  | | Value | Approximate Significance |
| Nominal by Nominal | Contingency Coefficient | .053 | .875 |
| N of Valid Cases | | 250 |  |

**Does visiting the maternity healthcare center take up a significant amount of your time?* WHO_recommendation_for_ANC_visits**

| **Crosstab** | | | | | |
| --- | --- | --- | --- | --- | --- |
|  | | | WHO_recommendation_for_ANC_visits | | Total |
|  | | | no | yes |  |
| Does visiting the maternity healthcare center take up a significant amount of your time? | I do not know‎ | Count | 6 | 0 | 6 |
|  |  | Expected Count | 3.9 | 2.1 | 6.0 |
|  | YES | Count | 84 | 44 | 128 |
|  |  | Expected Count | 84.0 | 44.0 | 128.0 |
|  | neutral | Count | 62 | 36 | 98 |
|  |  | Expected Count | 64.3 | 33.7 | 98.0 |
|  | NO | Count | 12 | 6 | 18 |
|  |  | Expected Count | 11.8 | 6.2 | 18.0 |
| Total | | Count | 164 | 86 | 250 |
|  | | Expected Count | 164.0 | 86.0 | 250.0 |

| **Chi-Square Tests** | | | |
| --- | --- | --- | --- |
|  | Value | df | Asymptotic Significance (2-sided) |
| Pearson Chi-Square | 3.392^a^ | 3 | .335 |
| Likelihood Ratio | 5.303 | 3 | .151 |
| Linear-by-Linear Association | .629 | 1 | .428 |
| N of Valid Cases | 250 |  |  |

| a. 2 cells (25.0%) have expected count less than 5. The minimum expected count is 2.06. |
| --- |

| **Symmetric Measures** | | | |
| --- | --- | --- | --- |
|  | | Value | Approximate Significance |
| Nominal by Nominal | Contingency Coefficient | .116 | .335 |
| N of Valid Cases | | 250 |  |

**Does attending the maternity healthcare center require significant financial expenses?* WHO_recommendation_for_ANC_visits**

| **Crosstab** | | | | | |
| --- | --- | --- | --- | --- | --- |
|  | | | WHO_recommendation_for_ANC_visits | | Total |
|  | | | no | yes |  |
| Does attending the maternity healthcare center require significant financial expenses? | I do not know‎ | Count | 6 | 0 | 6 |
|  |  | Expected Count | 3.9 | 2.1 | 6.0 |
|  | YES | Count | 67 | 41 | 108 |
|  |  | Expected Count | 70.8 | 37.2 | 108.0 |
|  | neutral | Count | 67 | 32 | 99 |
|  |  | Expected Count | 64.9 | 34.1 | 99.0 |
|  | NO | Count | 24 | 13 | 37 |
|  |  | Expected Count | 24.3 | 12.7 | 37.0 |
| Total | | Count | 164 | 86 | 250 |
|  | | Expected Count | 164.0 | 86.0 | 250.0 |

| **Chi-Square Tests** | | | |
| --- | --- | --- | --- |
|  | Value | df | Asymptotic Significance (2-sided) |
| Pearson Chi-Square | 3.952^a^ | 3 | .267 |
| Likelihood Ratio | 5.857 | 3 | .119 |
| Linear-by-Linear Association | .010 | 1 | .922 |
| N of Valid Cases | 250 |  |  |

| a. 2 cells (25.0%) have expected count less than 5. The minimum expected count is 2.06. |
| --- |

| **Symmetric Measures** | | | |
| --- | --- | --- | --- |
|  | | Value | Approximate Significance |
| Nominal by Nominal | Contingency Coefficient | .125 | .267 |
| N of Valid Cases | | 250 |  |

**Is attending the maternity healthcare center physically exhausting for you?* WHO_recommendation_for_ANC_visits**

| **Crosstab** | | | | | |
| --- | --- | --- | --- | --- | --- |
|  | | | WHO_recommendation_for_ANC_visits | | Total |
|  | | | no | yes |  |
| Is attending the maternity healthcare center physically exhausting for you? | I do not know‎ | Count | 7 | 0 | 7 |
|  |  | Expected Count | 4.6 | 2.4 | 7.0 |
|  | YES | Count | 89 | 51 | 140 |
|  |  | Expected Count | 91.8 | 48.2 | 140.0 |
|  | neutral | Count | 61 | 32 | 93 |
|  |  | Expected Count | 61.0 | 32.0 | 93.0 |
|  | NO | Count | 7 | 3 | 10 |
|  |  | Expected Count | 6.6 | 3.4 | 10.0 |
| Total | | Count | 164 | 86 | 250 |
|  | | Expected Count | 164.0 | 86.0 | 250.0 |

| **Chi-Square Tests** | | | |
| --- | --- | --- | --- |
|  | Value | df | Asymptotic Significance (2-sided) |
| Pearson Chi-Square | 4.012^a^ | 3 | .260 |
| Likelihood Ratio | 6.243 | 3 | .100 |
| Linear-by-Linear Association | .110 | 1 | .741 |
| N of Valid Cases | 250 |  |  |

| a. 3 cells (37.5%) have expected count less than 5. The minimum expected count is 2.41. |
| --- |

| **Symmetric Measures** | | | |
| --- | --- | --- | --- |
|  | | Value | Approximate Significance |
| Nominal by Nominal | Contingency Coefficient | .126 | .260 |
| N of Valid Cases | | 250 |  |
