# Supplementary material for: Antenatal Care Service Utilization Among Childbearing Women at El‐Digysab Village, El‐Jazeera State, Sudan, 2023
Source: J Pregnancy. 2026 Mar 27;2026:5565023. doi: 10.1155/jp/5565023 (PMC13140838; doi:10.1155/jp/5565023)
Supplement: Supplementary file 1 — Supporting Information 1 Additional supporting information can be found online in the Supporting Information section. Supporting Information A file containing all the data sheets and analysis outputs generated in the study, including the tests, frequencies, and descriptive statistics. [file JP-2026-5565023-s001.zip › Revised analysis output/Revised copy of quality vs ANC chi.docx]

CROSSTABS

/TABLES=‏‫

*. During your visits to healthcare centers during your most recent pregnancy, were you weighed?

1. During your visits to healthcare centers during your most recent pregnancy, was your blood pressure measured?
2. During your visits to healthcare centers during your most recent pregnancy, was a urine test conducted?
3. During your visits to healthcare centers during your most recent pregnancy, was a blood test performed?
4. During your visits to healthcare centers during your most recent pregnancy, did you understand the purpose of the tests conducted on you?
5. During your visits to healthcare centers during your most recent pregnancy, did you understand the purpose of the medications given to you?
6. During your visits to healthcare centers during your most recent pregnancy, were you informed about the natural changes that occur during pregnancy and childbirth, such as nausea, vomiting, etc.?
7. During your visits to healthcare centers during your most recent pregnancy, were you informed about the signs of pregnancy complications, such as anemia, seizures, etc.?
8. During your visits to healthcare centers during your most recent pregnancy, were you informed about where you should go in case complications arise?
9. During your visits to healthcare centers during your most recent pregnancy, were you given instructions regarding preparation for childbirth?
10. During your visits to healthcare centers during your most recent pregnancy, were you provided with instructions regarding nutrition?
11. During your visits to healthcare centers during your most recent pregnancy, were you given instructions regarding breastfeeding?
12. During your visits to healthcare centers during your most recent pregnancy, were you able to ask any questions?
13. During your visits to healthcare centers during your most recent pregnancy, were you able to discuss your concerns privately?
14. During your visits to healthcare centers during your most recent pregnancy, did you feel that the healthcare provider treated you with respect?
15. During your visits to healthcare centers during your most recent pregnancy, did you feel that the healthcare provider treated you differently for a personal reason?
16. During your visits to healthcare centers during your most recent pregnancy, did you feel that the facility (maternity care center) was clean?

‬

BY WHO_recommendation_for_ANC_visits

/FORMAT=AVALUE TABLES

/STATISTICS=CHISQ CC

/CELLS=COUNT EXPECTED

/COUNT ROUND CELL.

**Crosstabs**

| **Notes** | | |
| --- | --- | --- |
| Output Created | | 05-MAY-2023 21:23:33 |
| Comments | |  |
| Input | Data | C:\Users\hp\Documents\elmnagel medical day\SPSS DATA\ANC CLEANED Eyad.sav |
|  | Active Dataset | DataSet1 |
|  | Filter | <none> |
|  | Weight | <none> |
|  | Split File | <none> |
|  | N of Rows in Working Data File | 251 |
| Missing Value Handling | Definition of Missing | User-defined missing values are treated as missing. |
|  | Cases Used | Statistics for each table are based on all the cases with valid data in the specified range(s) for all variables in each table. |
| Syntax | | CROSSTABS  /TABLES=  During your visits to healthcare centers during your most recent pregnancy, were you weighed?   1. During your visits to healthcare centers during your most recent pregnancy, was your blood pressure measured? 2. During your visits to healthcare centers during your most recent pregnancy, was a urine test conducted? 3. During your visits to healthcare centers during your most recent pregnancy, was a blood test performed? 4. During your visits to healthcare centers during your most recent pregnancy, did you understand the purpose of the tests conducted on you? 5. During your visits to healthcare centers during your most recent pregnancy, did you understand the purpose of the medications given to you? 6. During your visits to healthcare centers during your most recent pregnancy, were you informed about the natural changes that occur during pregnancy and childbirth, such as nausea, vomiting, etc.? 7. During your visits to healthcare centers during your most recent pregnancy, were you informed about the signs of pregnancy complications, such as anemia, seizures, etc.? 8. During your visits to healthcare centers during your most recent pregnancy, were you informed about where you should go in case complications arise? 9. During your visits to healthcare centers during your most recent pregnancy, were you given instructions regarding preparation for childbirth? 10. During your visits to healthcare centers during your most recent pregnancy, were you provided with instructions regarding nutrition? 11. During your visits to healthcare centers during your most recent pregnancy, were you given instructions regarding breastfeeding? 12. During your visits to healthcare centers during your most recent pregnancy, were you able to ask any questions? 13. During your visits to healthcare centers during your most recent pregnancy, were you able to discuss your concerns privately? 14. During your visits to healthcare centers during your most recent pregnancy, did you feel that the healthcare provider treated you with respect? 15. During your visits to healthcare centers during your most recent pregnancy, did you feel that the healthcare provider treated you differently for a personal reason? 16. During your visits to healthcare centers during your most recent pregnancy, did you feel that the facility (maternity care center) was clean?   BY WHO_recommendation_for_ANC_visits  /FORMAT=AVALUE TABLES  /STATISTICS=CHISQ CC  /CELLS=COUNT EXPECTED  /COUNT ROUND CELL. |
| Resources | Processor Time | 00:00:00.06 |
|  | Elapsed Time | 00:00:00.04 |
|  | Dimensions Requested | 2 |
|  | Cells Available | 524245 |

| **Case Processing Summary** | | | | | | |
| --- | --- | --- | --- | --- | --- | --- |
|  | Cases | | | | | |
|  | Valid | | Missing | | Total | |
|  | N | Percent | N | Percent | N | Percent |
| During your visits to healthcare centers during your most recent pregnancy, were you weighed?  * WHO_recommendation_for_ANC_visits | 228 | 90.8% | 23 | 9.2% | 251 | 100.0% |
| During your visits to healthcare centers during your most recent pregnancy, was your blood pressure measured?_A * WHO_recommendation_for_ANC_visits | 228 | 90.8% | 23 | 9.2% | 251 | 100.0% |
| During your visits to healthcare centers during your most recent pregnancy, was a urine test conducted?  _B * WHO_recommendation_for_ANC_visits | 228 | 90.8% | 23 | 9.2% | 251 | 100.0% |
| During your visits to healthcare centers during your most recent pregnancy, was a blood test performed?_C * WHO_recommendation_for_ANC_visits | 227 | 90.4% | 24 | 9.6% | 251 | 100.0% |
| During your visits to healthcare centers during your most recent pregnancy, did you understand the purpose of the tests conducted on you?  _D * WHO_recommendation_for_ANC_visits | 227 | 90.4% | 24 | 9.6% | 251 | 100.0% |
| During your visits to healthcare centers during your most recent pregnancy, did you understand the purpose of the medications given to you?_E * WHO_recommendation_for_ANC_visits | 228 | 90.8% | 23 | 9.2% | 251 | 100.0% |
| During your visits to healthcare centers during your most recent pregnancy, were you informed about the natural changes that occur during pregnancy and childbirth, such as nausea, vomiting, etc.?  _F * WHO_recommendation_for_ANC_visits | 228 | 90.8% | 23 | 9.2% | 251 | 100.0% |
| During your visits to healthcare centers during your most recent pregnancy, were you informed about the signs of pregnancy complications, such as anemia, seizures, etc.?  _G * WHO_recommendation_for_ANC_visits | 227 | 90.4% | 24 | 9.6% | 251 | 100.0% |
| During your visits to healthcare centers during your most recent pregnancy, were you informed about where you should go in case complications arise?_H * WHO_recommendation_for_ANC_visits | 226 | 90.0% | 25 | 10.0% | 251 | 100.0% |
| During your visits to healthcare centers during your most recent pregnancy, were you given instructions regarding preparation for childbirth?_I * WHO_recommendation_for_ANC_visits | 227 | 90.4% | 24 | 9.6% | 251 | 100.0% |
| During your visits to healthcare centers during your most recent pregnancy, were you provided with instructions regarding nutrition?_J * WHO_recommendation_for_ANC_visits | 228 | 90.8% | 23 | 9.2% | 251 | 100.0% |
| During your visits to healthcare centers during your most recent pregnancy, were you given instructions regarding breastfeeding?_K * WHO_recommendation_for_ANC_visits | 228 | 90.8% | 23 | 9.2% | 251 | 100.0% |
| During your visits to healthcare centers during your most recent pregnancy, were you able to ask any questions?_L * WHO_recommendation_for_ANC_visits | 226 | 90.0% | 25 | 10.0% | 251 | 100.0% |
| During your visits to healthcare centers during your most recent pregnancy, were you able to discuss your concerns privately?  _M * WHO_recommendation_for_ANC_visits | 228 | 90.8% | 23 | 9.2% | 251 | 100.0% |
| During your visits to healthcare centers during your most recent pregnancy, did you feel that the healthcare provider treated you with respect?_N * WHO_recommendation_for_ANC_visits | 228 | 90.8% | 23 | 9.2% | 251 | 100.0% |
| During your visits to healthcare centers during your most recent pregnancy, did you feel that the healthcare provider treated you differently for a personal reason?  _O * WHO_recommendation_for_ANC_visits | 228 | 90.8% | 23 | 9.2% | 251 | 100.0% |
| During your visits to healthcare centers during your most recent pregnancy, did you feel that the facility (maternity care center) was clean?  _P * WHO_recommendation_for_ANC_visits | 228 | 90.8% | 23 | 9.2% | 251 | 100.0% |

**During your visits to healthcare centers during your most recent pregnancy, were you weighed?** *** WHO_recommendation_for_ANC_visits**

| **Crosstab** | | | | | |
| --- | --- | --- | --- | --- | --- |
|  | | | WHO_recommendation_for_ANC_visits | |  |
|  | | | no | yes |  |
| During your visits to healthcare centers during your most recent pregnancy, were you weighed? | I do not know/I do not remember | Count | 2 | 2 |  |
|  |  | Expected Count | 2.5 | 1.5 |  |
|  | Never | Count | 119 | 61 |  |
|  |  | Expected Count | 112.1 | 67.9 |  |
|  | few times | Count | 4 | 9 |  |
|  |  | Expected Count | 8.1 | 4.9 |  |
|  | most times | Count | 2 | 2 |  |
|  |  | Expected Count | 2.5 | 1.5 |  |
|  | every times | Count | 15 | 12 |  |
|  |  | Expected Count | 16.8 | 10.2 |  |
| Total | | Count | 142 | 86 |  |
|  | | Expected Count | 142.0 | 86.0 |  |

| **Chi-Square Tests** | | | |
| --- | --- | --- | --- |
|  | Value | df | Asymptotic Significance (2-sided) |
| Pearson Chi-Square | 7.653^a^ | 4 | .105 |
| Likelihood Ratio | 7.438 | 4 | .114 |
| Linear-by-Linear Association | 1.825 | 1 | .177 |
| N of Valid Cases | 228 |  |  |

| a. 5 cells (50.0%) have an expected count less than 5. The minimum expected count is 1.51. |
| --- |

| **Symmetric Measures** | | | |
| --- | --- | --- | --- |
|  | | Value | Approximate Significance |
| Nominal by Nominal | Contingency Coefficient | .180 | .105 |
| N of Valid Cases | | 228 |  |

**During your visits to healthcare centers during your most recent pregnancy, was your blood pressure messured?** **A * WHO_recommendation_for_ANC_visits**

| **Crosstab** | | | | | |
| --- | --- | --- | --- | --- | --- |
|  | | | WHO_recommendation_for_ANC_visits | |  |
|  | | | no | yes |  |
| During your visits to healthcare centers during your most recent pregnancy, was your blood pressure messured?_A | I do not know‎/ I do not remember | Count | 2 | 0 |  |
|  |  | Expected Count | 1.2 | .8 |  |
|  | Never | Count | 6 | 2 |  |
|  |  | Expected Count | 5.0 | 3.0 |  |
|  | few times | Count | 12 | 4 |  |
|  |  | Expected Count | 10.0 | 6.0 |  |
|  | most times | Count | 12 | 5 |  |
|  |  | Expected Count | 10.6 | 6.4 |  |
|  | every times | Count | 110 | 75 |  |
|  |  | Expected Count | 115.2 | 69.8 |  |
| Total | | Count | 142 | 86 |  |
|  | | Expected Count | 142.0 | 86.0 |  |

| **Chi-Square Tests** | | | |
| --- | --- | --- | --- |
|  | Value | df | Asymptotic Significance (2-sided) |
| Pearson Chi-Square | 3.990^a^ | 4 | .407 |
| Likelihood Ratio | 4.787 | 4 | .310 |
| Linear-by-Linear Association | 3.621 | 1 | .057 |
| N of Valid Cases | 228 |  |  |

| a. 4 cells (40.0%) have expected count less than 5. The minimum expected count is .75. |
| --- |

| **Symmetric Measures** | | | |
| --- | --- | --- | --- |
|  | | Value | Approximate Significance |
| Nominal by Nominal | Contingency Coefficient | .131 | .407 |
| N of Valid Cases | | 228 |  |

**During your visits to healthcare centers during your most recent pregnancy, was a urine test conducted?** **_B * WHO_recommendation_for_ANC_visits**

| **Crosstab** | | | | | |
| --- | --- | --- | --- | --- | --- |
|  | | | WHO_recommendation_for_ANC_visits | |  |
|  | | | no | yes |  |
| During your visits to healthcare centers during your most recent pregnancy, was a urine test conducted?_B | I do not know‎/ I do not remember | Count | 2 | 0 |  |
|  |  | Expected Count | 1.2 | .8 |  |
|  | Never | Count | 6 | 2 |  |
|  |  | Expected Count | 5.0 | 3.0 |  |
|  | few times | Count | 11 | 8 |  |
|  |  | Expected Count | 11.8 | 7.2 |  |
|  | most times | Count | 11 | 7 |  |
|  |  | Expected Count | 11.2 | 6.8 |  |
|  | every times | Count | 112 | 69 |  |
|  |  | Expected Count | 112.7 | 68.3 |  |
| Total | | Count | 142 | 86 |  |
|  | | Expected Count | 142.0 | 86.0 |  |

| **Chi-Square Tests** | | | |
| --- | --- | --- | --- |
|  | Value | df | Asymptotic Significance (2-sided) |
| Pearson Chi-Square | 1.941^a^ | 4 | .747 |
| Likelihood Ratio | 2.655 | 4 | .617 |
| Linear-by-Linear Association | .454 | 1 | .500 |
| N of Valid Cases | 228 |  |  |

| a. 4 cells (40.0%) have expected count less than 5. The minimum expected count is .75. |
| --- |

| **Symmetric Measures** | | | |
| --- | --- | --- | --- |
|  | | Value | Approximate Significance |
| Nominal by Nominal | Contingency Coefficient | .092 | .747 |
| N of Valid Cases | | 228 |  |

**During your visits to healthcare centers during your most recent pregnancy, was a blood test performed?_C * WHO_recommendation_for_ANC_visits**

| **Crosstab** | | | | | |
| --- | --- | --- | --- | --- | --- |
|  | | | WHO_recommendation_for_ANC_visits | |  |
|  | | | no | yes |  |
| During your visits to healthcare centers during your most recent pregnancy, was a blood test performed?_C | I do not know‎/ I do not remember | Count | 2 | 0 |  |
|  |  | Expected Count | 1.2 | .8 |  |
|  | Never | Count | 5 | 1 |  |
|  |  | Expected Count | 3.7 | 2.3 |  |
|  | few times | Count | 10 | 7 |  |
|  |  | Expected Count | 10.6 | 6.4 |  |
|  | most times | Count | 10 | 9 |  |
|  |  | Expected Count | 11.8 | 7.2 |  |
|  | every times | Count | 114 | 69 |  |
|  |  | Expected Count | 113.7 | 69.3 |  |
| Total | | Count | 141 | 86 |  |
|  | | Expected Count | 141.0 | 86.0 |  |

| **Chi-Square Tests** | | | |
| --- | --- | --- | --- |
|  | Value | df | Asymptotic Significance (2-sided) |
| Pearson Chi-Square | 3.175^a^ | 4 | .529 |
| Likelihood Ratio | 3.989 | 4 | .408 |
| Linear-by-Linear Association | .449 | 1 | .503 |
| N of Valid Cases | 227 |  |  |

| a. 4 cells (40.0%) have expected count less than 5. The minimum expected count is .76. |
| --- |

| **Symmetric Measures** | | | |
| --- | --- | --- | --- |
|  | | Value | Approximate Significance |
| Nominal by Nominal | Contingency Coefficient | .117 | .529 |
| N of Valid Cases | | 227 |  |

**During your visits to healthcare centers during your most recent pregnancy, did you understand the purpose of the tests conducted on you?_D * WHO_recommendation_for_ANC_visits**

| **Crosstab** | | | | | |
| --- | --- | --- | --- | --- | --- |
|  | | | WHO_recommendation_for_ANC_visits | |  |
|  | | | no | yes |  |
| During your visits to healthcare centers during your most recent pregnancy, did you understand the purpose of the tests conducted on you?_D | I do not know‎/ I do not remember | Count | 3 | 3 |  |
|  |  | Expected Count | 3.7 | 2.3 |  |
|  | Never | Count | 49 | 20 |  |
|  |  | Expected Count | 42.9 | 26.1 |  |
|  | few times | Count | 19 | 10 |  |
|  |  | Expected Count | 18.0 | 11.0 |  |
|  | most times | Count | 10 | 7 |  |
|  |  | Expected Count | 10.6 | 6.4 |  |
|  | every times | Count | 59 | 46 |  |
|  |  | Expected Count | 65.2 | 39.8 |  |
|  | no lab test was performed | Count | 1 | 0 |  |
|  |  | Expected Count | .6 | .4 |  |
| Total | | Count | 141 | 86 |  |
|  | | Expected Count | 141.0 | 86.0 |  |

| **Chi-Square Tests** | | | |
| --- | --- | --- | --- |
|  | Value | df | Asymptotic Significance (2-sided) |
| Pearson Chi-Square | 5.093^a^ | 5 | .405 |
| Likelihood Ratio | 5.488 | 5 | .359 |
| Linear-by-Linear Association | 2.627 | 1 | .105 |
| N of Valid Cases | 227 |  |  |

| a. 4 cells (33.3%) have expected count less than 5. The minimum expected count is .38. |
| --- |

| **Symmetric Measures** | | | |
| --- | --- | --- | --- |
|  | | Value | Approximate Significance |
| Nominal by Nominal | Contingency Coefficient | .148 | .405 |
| N of Valid Cases | | 227 |  |

**During your visits to healthcare centers during your most recent pregnancy, did you understand the purpose of the medications given to you?_E * WHO_recommendation_for_ANC_visits**

| **Crosstab** | | | | | |
| --- | --- | --- | --- | --- | --- |
|  | | | WHO_recommendation_for_ANC_visits | |  |
|  | | | no | yes |  |
| During your visits to healthcare centers during your most recent pregnancy, did you understand the purpose of the medications given to you?_E | I do not know‎/ I do not remember | Count | 2 | 1 |  |
|  |  | Expected Count | 1.9 | 1.1 |  |
|  | Never | Count | 23 | 8 |  |
|  |  | Expected Count | 19.3 | 11.7 |  |
|  | few times | Count | 13 | 6 |  |
|  |  | Expected Count | 11.8 | 7.2 |  |
|  | most times | Count | 15 | 5 |  |
|  |  | Expected Count | 12.5 | 7.5 |  |
|  | every times | Count | 88 | 64 |  |
|  |  | Expected Count | 94.7 | 57.3 |  |
|  | no medications were prescribed | Count | 1 | 2 |  |
|  |  | Expected Count | 1.9 | 1.1 |  |
| Total | | Count | 142 | 86 |  |
|  | | Expected Count | 142.0 | 86.0 |  |

| **Chi-Square Tests** | | | |
| --- | --- | --- | --- |
|  | Value | df | Asymptotic Significance (2-sided) |
| Pearson Chi-Square | 5.894^a^ | 5 | .317 |
| Likelihood Ratio | 6.034 | 5 | .303 |
| Linear-by-Linear Association | 4.067 | 1 | .044 |
| N of Valid Cases | 228 |  |  |

| a. 4 cells (33.3%) have expected count less than 5. The minimum expected count is 1.13. |
| --- |

| **Symmetric Measures** | | | |
| --- | --- | --- | --- |
|  | | Value | Approximate Significance |
| Nominal by Nominal | Contingency Coefficient | .159 | .317 |
| N of Valid Cases | | 228 |  |

**During your visits to healthcare centers during your most recent pregnancy, were you informed about the natural changes that occur during pregnancy and childbirth, such as nausea, vomiting, etc.?**

**_F * WHO_recommendation_for_ANC_visits**

| **Crosstab** | | | | | |
| --- | --- | --- | --- | --- | --- |
|  | | | WHO_recommendation_for_ANC_visits | | Total |
|  | | | no | yes |  |
| فDuring your visits to healthcare centers during your most recent pregnancy, were you informed about the natural changes that occur during pregnancy and childbirth, such as nausea, vomiting, etc.?_F | I do not know‎ | Count | 2 | 1 | 3 |
|  |  | Expected Count | 1.9 | 1.1 | 3.0 |
|  | NO | Count | 54 | 23 | 77 |
|  |  | Expected Count | 48.0 | 29.0 | 77.0 |
|  | YES | Count | 86 | 62 | 148 |
|  |  | Expected Count | 92.2 | 55.8 | 148.0 |
| Total | | Count | 142 | 86 | 228 |
|  | | Expected Count | 142.0 | 86.0 | 228.0 |

| **Chi-Square Tests** | | | |
| --- | --- | --- | --- |
|  | Value | df | Asymptotic Significance (2-sided) |
| Pearson Chi-Square | 3.141^a^ | 2 | .208 |
| Likelihood Ratio | 3.194 | 2 | .202 |
| Linear-by-Linear Association | 2.868 | 1 | .090 |
| N of Valid Cases | 228 |  |  |

| a. 2 cells (33.3%) have expected count less than 5. The minimum expected count is 1.13. |
| --- |

| **Symmetric Measures** | | | |
| --- | --- | --- | --- |
|  | | Value | Approximate Significance |
| Nominal by Nominal | Contingency Coefficient | .117 | .208 |
| N of Valid Cases | | 228 |  |

**During your visits to healthcare centers during your most recent pregnancy, were you informed about the signs of pregnancy complications, such as anemia, seizures, etc.?_G * WHO_recommendation_for_ANC_visits**

| **Crosstab** | | | | | |
| --- | --- | --- | --- | --- | --- |
|  | | | WHO_recommendation_for_ANC_visits | | Total |
|  | | | no | yes |  |
| During your visits to healthcare centers during your most recent pregnancy, were you informed about the signs of pregnancy complications, such as anemia, seizures, etc.?_G | I do not know‎ | Count | 2 | 0 | 2 |
|  |  | Expected Count | 1.2 | .8 | 2.0 |
|  | NO | Count | 81 | 51 | 132 |
|  |  | Expected Count | 82.0 | 50.0 | 132.0 |
|  | YES | Count | 58 | 35 | 93 |
|  |  | Expected Count | 57.8 | 35.2 | 93.0 |
| Total | | Count | 141 | 86 | 227 |
|  | | Expected Count | 141.0 | 86.0 | 227.0 |

| **Chi-Square Tests** | | | |
| --- | --- | --- | --- |
|  | Value | df | Asymptotic Significance (2-sided) |
| Pearson Chi-Square | 1.254^a^ | 2 | .534 |
| Likelihood Ratio | 1.939 | 2 | .379 |
| Linear-by-Linear Association | .020 | 1 | .888 |
| N of Valid Cases | 227 |  |  |

| a. 2 cells (33.3%) have expected count less than 5. The minimum expected count is .76. |
| --- |

| **Symmetric Measures** | | | |
| --- | --- | --- | --- |
|  | | Value | Approximate Significance |
| Nominal by Nominal | Contingency Coefficient | .074 | .534 |
| N of Valid Cases | | 227 |  |

**During your visits to healthcare centers during your most recent pregnancy, were you informed about where you should go in case complications arise?_H * WHO_recommendation_for_ANC_visits**

| **Crosstab** | | | | | |
| --- | --- | --- | --- | --- | --- |
|  | | | WHO_recommendation_for_ANC_visits | | Total |
|  | | | no | yes |  |
| During your visits to healthcare centers during your most recent pregnancy, were you informed about where you should go in case complications arise?_H | I do not know‎ | Count | 5 | 3 | 8 |
|  |  | Expected Count | 5.0 | 3.0 | 8.0 |
|  | NO | Count | 78 | 46 | 124 |
|  |  | Expected Count | 76.8 | 47.2 | 124.0 |
|  | YES | Count | 57 | 37 | 94 |
|  |  | Expected Count | 58.2 | 35.8 | 94.0 |
| Total | | Count | 140 | 86 | 226 |
|  | | Expected Count | 140.0 | 86.0 | 226.0 |

| **Chi-Square Tests** | | | |
| --- | --- | --- | --- |
|  | Value | df | Asymptotic Significance (2-sided) |
| Pearson Chi-Square | .117^a^ | 2 | .943 |
| Likelihood Ratio | .117 | 2 | .943 |
| Linear-by-Linear Association | .099 | 1 | .753 |
| N of Valid Cases | 226 |  |  |

| a. 2 cells (33.3%) have expected count less than 5. The minimum expected count is 3.04. |
| --- |

| **Symmetric Measures** | | | |
| --- | --- | --- | --- |
|  | | Value | Approximate Significance |
| Nominal by Nominal | Contingency Coefficient | .023 | .943 |
| N of Valid Cases | | 226 |  |

**During your visits to healthcare centers during your most recent pregnancy, were you given instructions regarding preparation for childbirth?_I * WHO_recommendation_for_ANC_visits**

| **Crosstab** | | | | | |
| --- | --- | --- | --- | --- | --- |
|  | | | WHO_recommendation_for_ANC_visits | | Total |
|  | | | no | yes |  |
| During your visits to healthcare centers during your most recent pregnancy, were you given instructions regarding preparation for childbirth?_I | I do not know‎ | Count | 2 | 0 | 2 |
|  |  | Expected Count | 1.2 | .8 | 2.0 |
|  | NO | Count | 90 | 48 | 138 |
|  |  | Expected Count | 85.7 | 52.3 | 138.0 |
|  | YES | Count | 49 | 38 | 87 |
|  |  | Expected Count | 54.0 | 33.0 | 87.0 |
| Total | | Count | 141 | 86 | 227 |
|  | | Expected Count | 141.0 | 86.0 | 227.0 |

| **Chi-Square Tests** | | | |
| --- | --- | --- | --- |
|  | Value | df | Asymptotic Significance (2-sided) |
| Pearson Chi-Square | 3.025^a^ | 2 | .220 |
| Likelihood Ratio | 3.695 | 2 | .158 |
| Linear-by-Linear Association | 2.487 | 1 | .115 |
| N of Valid Cases | 227 |  |  |

| a. 2 cells (33.3%) have expected count less than 5. The minimum expected count is .76. |
| --- |

| **Symmetric Measures** | | | |
| --- | --- | --- | --- |
|  | | Value | Approximate Significance |
| Nominal by Nominal | Contingency Coefficient | .115 | .220 |
| N of Valid Cases | | 227 |  |

**During your visits to healthcare centers during your most recent pregnancy, were you provided with instructions regarding nutrition?_J * WHO_recommendation_for_ANC_visits**

| **Crosstab** | | | | | |
| --- | --- | --- | --- | --- | --- |
|  | | | WHO_recommendation_for_ANC_visits | | Total |
|  | | | no | yes |  |
| During your visits to healthcare centers during your most recent pregnancy, were you provided with instructions regarding nutrition?_J | I do not know‎ | Count | 3 | 0 | 3 |
|  |  | Expected Count | 1.9 | 1.1 | 3.0 |
|  | NO | Count | 60 | 23 | 83 |
|  |  | Expected Count | 51.7 | 31.3 | 83.0 |
|  | YES | Count | 79 | 63 | 142 |
|  |  | Expected Count | 88.4 | 53.6 | 142.0 |
| Total | | Count | 142 | 86 | 228 |
|  | | Expected Count | 142.0 | 86.0 | 228.0 |

| **Chi-Square Tests** | | | |
| --- | --- | --- | --- |
|  | Value | df | Asymptotic Significance (2-sided) |
| Pearson Chi-Square | 8.027^a^ | 2 | .018 |
| Likelihood Ratio | 9.158 | 2 | .010 |
| Linear-by-Linear Association | 7.858 | 1 | .005 |
| N of Valid Cases | 228 |  |  |

| a. 2 cells (33.3%) have expected count less than 5. The minimum expected count is 1.13. |
| --- |

| **Symmetric Measures** | | | |
| --- | --- | --- | --- |
|  | | Value | Approximate Significance |
| Nominal by Nominal | Contingency Coefficient | .184 | .018 |
| N of Valid Cases | | 228 |  |

**During your visits to healthcare centers during your most recent pregnancy, were you given instructions regarding breastfeeding?_K * WHO_recommendation_for_ANC_visits**

| **Crosstab** | | | | | |
| --- | --- | --- | --- | --- | --- |
|  | | | WHO_recommendation_for_ANC_visits | | Total |
|  | | | no | yes |  |
| During your visits to healthcare centers during your most recent pregnancy, were you given instructions regarding breastfeeding?_K | I do not know‎ | Count | 1 | 0 | 1 |
|  |  | Expected Count | .6 | .4 | 1.0 |
|  | NO | Count | 100 | 58 | 158 |
|  |  | Expected Count | 98.4 | 59.6 | 158.0 |
|  | YES | Count | 41 | 28 | 69 |
|  |  | Expected Count | 43.0 | 26.0 | 69.0 |
| Total | | Count | 142 | 86 | 228 |
|  | | Expected Count | 142.0 | 86.0 | 228.0 |

| **Chi-Square Tests** | | | |
| --- | --- | --- | --- |
|  | Value | df | Asymptotic Significance (2-sided) |
| Pearson Chi-Square | .915^a^ | 2 | .633 |
| Likelihood Ratio | 1.254 | 2 | .534 |
| Linear-by-Linear Association | .471 | 1 | .492 |
| N of Valid Cases | 228 |  |  |

| a. 2 cells (33.3%) have expected count less than 5. The minimum expected count is .38. |
| --- |

| **Symmetric Measures** | | | |
| --- | --- | --- | --- |
|  | | Value | Approximate Significance |
| Nominal by Nominal | Contingency Coefficient | .063 | .633 |
| N of Valid Cases | | 228 |  |

**During your visits to healthcare centers during your most recent pregnancy, were you able to ask any questions?_L * WHO_recommendation_for_ANC_visits**

| **Crosstab** | | | | | |
| --- | --- | --- | --- | --- | --- |
|  | | | WHO_recommendation_for_ANC_visits | |  |
|  | | | no | yes |  |
| During your visits to healthcare centers during your most recent pregnancy, were you able to ask any questions?_L | I do not know‎/ I do not remember | Count | 1 | 0 |  |
|  |  | Expected Count | .6 | .4 |  |
|  | Never | Count | 11 | 2 |  |
|  |  | Expected Count | 8.1 | 4.9 |  |
|  | few times | Count | 11 | 12 |  |
|  |  | Expected Count | 14.3 | 8.7 |  |
|  | most times | Count | 12 | 10 |  |
|  |  | Expected Count | 13.7 | 8.3 |  |
|  | every times | Count | 106 | 61 |  |
|  |  | Expected Count | 104.2 | 62.8 |  |
| Total | | Count | 141 | 85 |  |
|  | | Expected Count | 141.0 | 85.0 |  |

| **Chi-Square Tests** | | | |
| --- | --- | --- | --- |
|  | Value | df | Asymptotic Significance (2-sided) |
| Pearson Chi-Square | 6.079^a^ | 4 | .193 |
| Likelihood Ratio | 6.726 | 4 | .151 |
| Linear-by-Linear Association | .067 | 1 | .795 |
| N of Valid Cases | 226 |  |  |

| a. 3 cells (30.0%) have expected count less than 5. The minimum expected count is .38. |
| --- |

| **Symmetric Measures** | | | |
| --- | --- | --- | --- |
|  | | Value | Approximate Significance |
| Nominal by Nominal | Contingency Coefficient | .162 | .193 |
| N of Valid Cases | | 226 |  |

During your visits to healthcare centers during your most recent pregnancy, **were you able to discuss your concerns privately?_M * WHO_recommendation_for_ANC_visits**

| **Crosstab** | | | | | |
| --- | --- | --- | --- | --- | --- |
|  | | | WHO_recommendation_for_ANC_visits | |  |
|  | | | no | yes |  |
| During your visits to healthcare centers during your most recent pregnancy, were you able to discuss your concerns privately?**_**_M | I do not know‎/ I do not remember | Count | 1 | 0 |  |
|  |  | Expected Count | .6 | .4 |  |
|  | Never | Count | 15 | 8 |  |
|  |  | Expected Count | 14.3 | 8.7 |  |
|  | few times | Count | 13 | 8 |  |
|  |  | Expected Count | 13.1 | 7.9 |  |
|  | most times | Count | 13 | 11 |  |
|  |  | Expected Count | 14.9 | 9.1 |  |
|  | every times | Count | 100 | 59 |  |
|  |  | Expected Count | 99.0 | 60.0 |  |
| Total | | Count | 142 | 86 |  |
|  | | Expected Count | 142.0 | 86.0 |  |

| **Chi-Square Tests** | | | |
| --- | --- | --- | --- |
|  | Value | df | Asymptotic Significance (2-sided) |
| Pearson Chi-Square | 1.389^a^ | 4 | .846 |
| Likelihood Ratio | 1.716 | 4 | .788 |
| Linear-by-Linear Association | .035 | 1 | .851 |
| N of Valid Cases | 228 |  |  |

| a. 2 cells (20.0%) have expected count less than 5. The minimum expected count is .38. |
| --- |

| **Symmetric Measures** | | | |
| --- | --- | --- | --- |
|  | | Value | Approximate Significance |
| Nominal by Nominal | Contingency Coefficient | .078 | .846 |
| N of Valid Cases | | 228 |  |

**During your visits to healthcare centers during your most recent pregnancy, did you feel that the healthcare provider treated you with respect?_N * WHO_recommendation_for_ANC_visits**

| **Crosstab** | | | | | |
| --- | --- | --- | --- | --- | --- |
|  | | | WHO_recommendation_for_ANC_visits | |  |
|  | | | no | yes |  |
| During your visits to healthcare centers during your most recent pregnancy, did you feel that the healthcare provider treated you with respect?_N | I do not know‎/ I do not remember | Count | 1 | 0 |  |
|  |  | Expected Count | .6 | .4 |  |
|  | Never | Count | 10 | 2 |  |
|  |  | Expected Count | 7.5 | 4.5 |  |
|  | few times | Count | 5 | 0 |  |
|  |  | Expected Count | 3.1 | 1.9 |  |
|  | most times | Count | 6 | 6 |  |
|  |  | Expected Count | 7.5 | 4.5 |  |
|  | every times | Count | 120 | 78 |  |
|  |  | Expected Count | 123.3 | 74.7 |  |
| Total | | Count | 142 | 86 |  |
|  | | Expected Count | 142.0 | 86.0 |  |

| **Chi-Square Tests** | | | |
| --- | --- | --- | --- |
|  | Value | df | Asymptotic Significance (2-sided) |
| Pearson Chi-Square | 6.905^a^ | 4 | .141 |
| Likelihood Ratio | 9.221 | 4 | .056 |
| Linear-by-Linear Association | 3.949 | 1 | .047 |
| N of Valid Cases | 228 |  |  |

| a. 6 cells (60.0%) have an expected count less than 5. The minimum expected count is.38. |
| --- |

| **Symmetric Measures** | | | |
| --- | --- | --- | --- |
|  | | Value | Approximate Significance |
| Nominal by Nominal | Contingency Coefficient | .171 | .141 |
| N of Valid Cases | | 228 |  |

**During your visits to healthcare centers during your most recent pregnancy, did you feel that the healthcare provider treated you differently for a personal reason?_O * WHO_recommendation_for_ANC_visits**

| **Crosstab** | | | | | |
| --- | --- | --- | --- | --- | --- |
|  | | | WHO_recommendation_for_ANC_visits | |  |
|  | | | no | yes |  |
| During your visits to healthcare centers during your most recent pregnancy, did you feel that the healthcare provider treated you differently for a personal reason?_O | I do not know‎/ I do not remember | Count | 3 | 1 |  |
|  |  | Expected Count | 2.5 | 1.5 |  |
|  | Never | Count | 120 | 69 |  |
|  |  | Expected Count | 117.7 | 71.3 |  |
|  | few times | Count | 1 | 1 |  |
|  |  | Expected Count | 1.2 | .8 |  |
|  | most times | Count | 2 | 1 |  |
|  |  | Expected Count | 1.9 | 1.1 |  |
|  | every times | Count | 16 | 14 |  |
|  |  | Expected Count | 18.7 | 11.3 |  |
| Total | | Count | 142 | 86 |  |
|  | | Expected Count | 142.0 | 86.0 |  |

| **Chi-Square Tests** | | | |
| --- | --- | --- | --- |
|  | Value | df | Asymptotic Significance (2-sided) |
| Pearson Chi-Square | 1.569^a^ | 4 | .814 |
| Likelihood Ratio | 1.558 | 4 | .816 |
| Linear-by-Linear Association | 1.241 | 1 | .265 |
| N of Valid Cases | 228 |  |  |

| a. 6 cells (60.0%) have expected count less than 5. The minimum expected count is .75. |
| --- |

| **Symmetric Measures** | | | |
| --- | --- | --- | --- |
|  | | Value | Approximate Significance |
| Nominal by Nominal | Contingency Coefficient | .083 | .814 |
| N of Valid Cases | | 228 |  |

**During your visits to healthcare centers during your most recent pregnancy, did you feel that the facility (maternity care center) was clean?_P * WHO_recommendation_for_ANC_visits**

| **Crosstab** | | | | | |
| --- | --- | --- | --- | --- | --- |
|  | | | WHO_recommendation_for_ANC_visits | |  |
|  | | | no | yes |  |
| During your visits to healthcare centers during your most recent pregnancy, did you feel that the facility (maternity care center) was clean?_P | I do not know‎/ I do not remember | Count | 1 | 2 |  |
|  |  | Expected Count | 1.9 | 1.1 |  |
|  | Never | Count | 11 | 6 |  |
|  |  | Expected Count | 10.6 | 6.4 |  |
|  | few times | Count | 5 | 2 |  |
|  |  | Expected Count | 4.4 | 2.6 |  |
|  | most times | Count | 12 | 11 |  |
|  |  | Expected Count | 14.3 | 8.7 |  |
|  | every times | Count | 113 | 65 |  |
|  |  | Expected Count | 110.9 | 67.1 |  |
| Total | | Count | 142 | 86 |  |
|  | | Expected Count | 142.0 | 86.0 |  |

| **Chi-Square Tests** | | | |
| --- | --- | --- | --- |
|  | Value | df | Asymptotic Significance (2-sided) |
| Pearson Chi-Square | 2.472^a^ | 4 | .650 |
| Likelihood Ratio | 2.413 | 4 | .660 |
| Linear-by-Linear Association | .219 | 1 | .640 |
| N of Valid Cases | 228 |  |  |

| a. 4 cells (40.0%) have expected count less than 5. The minimum expected count is 1.13. |
| --- |

| **Symmetric Measures** | | | |
| --- | --- | --- | --- |
|  | | Value | Approximate Significance |
| Nominal by Nominal | Contingency Coefficient | .104 | .650 |
| N of Valid Cases | | 228 |  |
