# Supplementary material for: Antenatal Care Service Utilization Among Childbearing Women at El‐Digysab Village, El‐Jazeera State, Sudan, 2023
Source: J Pregnancy. 2026 Mar 27;2026:5565023. doi: 10.1155/jp/5565023 (PMC13140838; doi:10.1155/jp/5565023)
Supplement: Supplementary file 1 — Supporting Information 1 Additional supporting information can be found online in the Supporting Information section. Supporting Information A file containing all the data sheets and analysis outputs generated in the study, including the tests, frequencies, and descriptive statistics. [file JP-2026-5565023-s001.zip › Revised analysis output/[Final] descriptives.docx]

GET

FILE='C:\Users\hp\Documents\elmnagel medical day\SPSS DATA\ANC CLEANED Eyad.sav'.

DATASET NAME DataSet1 WINDOW=FRONT.

DESCRIPTIVES VARIABLES= ‏‫ How many weeks did your last pregnancy last? How old are you? How many pregnancies did you have? How many miscarriages did you have? How many live births did you have? How many ANC visits did you have during your last pregnancy? When was your first ANC visit during your last pregnancy? If the answer is other kindly mention it ‬ ‏‫_A‬

/STATISTICS=MEAN STDDEV MIN MAX.

**Descriptives**

| **Notes** | | |
| --- | --- | --- |
| Output Created | | 01-MAY-2023 13:57:44 |
| Comments | |  |
| Input | Data | C:\Users\hp\Documents\elmnagel medical day\SPSS DATA\ANC CLEANED Eyad.sav |
|  | Active Dataset | DataSet1 |
|  | Filter | <none> |
|  | Weight | <none> |
|  | Split File | <none> |
|  | N of Rows in Working Data File | 251 |
| Missing Value Handling | Definition of Missing | User defined missing values are treated as missing. |
|  | Cases Used | All non-missing data are used. |
| Syntax | | DESCRIPTIVES VARIABLES= How many weeks did your last pregnancy last? How old are you? How many pregnancies did you have? How many miscarriages did you have? How many live births did you have? How many ANC visits did you have during your last pregnancy? When was your first ANC visit during your last pregnancy? If the answer is other kindly mention it  /STATISTICS=MEAN STDDEV MIN MAX. |
| Resources | Processor Time | 00:00:00.00 |
|  | Elapsed Time | 00:00:00.00 |

[DataSet1] C:\Users\hp\Documents\elmnagel medical day\SPSS DATA\ANC CLEANED Eyad.sav

| **Descriptive Statistics** | | | | | |
| --- | --- | --- | --- | --- | --- |
|  | N | Minimum | Maximum | Mean | Std. Deviation |
| How many weeks did your last pregnancy last? | 239 | 13 | 46 | 35.58 | 7.295 |
| How old are you? | 248 | 18 | 45 | 26.01 | 5.475 |
| How many pregnancies did you have? | 251 | 1 | 13 | 4.82 | 2.623 |
| How many miscarriages did you have? | 251 | 0 | 4 | .60 | .917 |
| How many live births did you have? | 247 | 0 | 11 | 4.08 | 2.427 |
| How many ANC visits did you have during your last pregnancy? | 229 | 0 | 1 | .38 | .485 |
| When was your first ANC visit during your last pregnancy? | 225 | 0 | 69 | 15.38 | 9.611 |
| If the answer is other kindly mention it | 0 |  |  |  |  |
| Valid N (listwise) | 0 |  |  |  |  |

FILE='C:\Users\hp\Documents\elmnagel medical day\SPSS DATA\ANC cleaned.sav'.

DATASET NAME DataSet1 WINDOW=FRONT.

SAVE OUTFILE='C:\Users\hp\Documents\elmnagel medical day\SPSS DATA\ANC CLEANED Eyad.sav'

/VERSION=2

/COMPRESSED.

FREQUENCIES VARIABLES=When was the last pregnancy? How many weeks did the last pregnancy last?How old are you?Can you obtain the information you need about pregnancy from family or friends?

Are antenatal care appointments too short and rushed?Are the operating hours of the maternity care center convenient for you? Are there long waiting times at the maternity care center? Do you have confidence in your healthcare provider?Do you think that the people who provide maternity care understand your health issues?

‏Do you have someone to take care of your children while you go to the maternity care centre? ‫Do you have other work commitments that take priority over Antenatal care? Does your husband have a negative view of Antenatal care services? Does your mother have a negative view of Antenatal care services? Do your peers have a negative view of Antenatal care services? Does attending the maternity care centre take up a lot of your time? Does attending the maternity care centre cost a lot of money? Is attending the maternity care centre physically exhausting?‬?

During the visits to care centers during your last pregnancy, was your weight measured? During the visits to care centers during your last pregnancy, was your blood pressure measured?_A‬

‏‫During the visits to care centers during your last pregnancy, was a urine test conducted?_B‬

During the visits to care centers during your last pregnancy, was a blood test conducted?_C‬

‏‫During the visits to care centers during your last pregnancy, did you understand the purpose of the tests being conducted on you? During your last pregnancy, did you understand the purpose of the medications given to you?_E‬

‏‫During the visits to care centers during your last pregnancy, were you informed about the natural changes during pregnancy and childbirth, such as nausea, vomiting, etc.?_F

‬ During the visits to care centers during your last pregnancy, were you informed about the signs of pregnancy complications, such as anemia, seizures, etc.?_G‬

During the visits to care centers during your last pregnancy, were you informed about where you should go in case of complications?_H

‏‫During the visits to care centers during your last pregnancy, were you provided with instructions on preparing for childbirth?_I‬

‏‫During the visits to care centers during your last pregnancy, were you provided with instructions on nutrition?_J

‬ ‏‫During the visits to care centers during your last pregnancy, were you provided with instructions on breastfeeding?_K‬

‏‫During the visits to care centers during your last pregnancy, were you able to ask any questions?_L

‬ ‏‫During the visits to care centers during your last pregnancy, were you able to discuss your concerns privately?_M‬

‏‫During the visits to care centers during your last pregnancy, did you feel that the healthcare provider treated you with respect?_N‬

‏‫During the visits to care centers during your last pregnancy, did you feel that the healthcare provider treated you differently for a personal reason?_O‬

‏‫During the visits to care centers during your last pregnancy, did you feel that the facility (the maternity care center) was clean?_P

‬ Have you been circumcised (FGM)?

WHO_recommendation_for_ANC_visits **What is your marital status?**

What is your educational level?What is your husband's educational level? What is your occupation?

What is your husband's occupation?How many times have you been pregnant? How many times have you had a miscarriage?How many children have you given birth to alive? ‏During your last pregnancy, did you experience anemia?

During your last pregnancy, did you experience cramps?

During your last pregnancy, did you experience thyroid diseases or their complications?During your last pregnancy, did you experience preterm labor or incomplete delivery?During your last pregnancy, did you experience obstructed or difficult labor?During your last pregnancy, did you experience shoulder dystocia?During your last pregnancy, did you experience a large baby weighing ≥ 4.5 kg?During your last pregnancy, did you experience postpartum hemorrhage?

During your last pregnancy, did you experience intrauterine growth restriction (IUGR) or halted fetal growth?During your last pregnancy, did you experience intrauterine fetal death?Did you experience any other complications? If so, what were they?‬ Did you receive antenatal care during your last pregnancy?Who was the healthcare provider during your pregnancy?If the answer is "Other," what is it?What type of healthcare services were provided?

Do you have health insurance?‬What mode of transportation do you use to reach the maternity healthcare center?‬A_ If the answer is "other," what is it? How does Antenatal care affect the health of the mother and the baby? Do you often forget your appointment dates?‬

/FORMAT=NOTABLE

/ORDER=ANALYSIS.

**Frequencies**

| **Notes** | | |
| --- | --- | --- |
| Output Created | | 14-APR-2023 01:28:03 |
| Comments | |  |
| Input | Data | C:\Users\hp\Documents\elmnagel medical day\SPSS DATA\ANC CLEANED Eyad.sav |
|  | Active Dataset | DataSet1 |
|  | Filter | <none> |
|  | Weight | <none> |
|  | Split File | <none> |
|  | N of Rows in Working Data File | 251 |
| Missing Value Handling | Definition of Missing | User-defined missing values are treated as missing. |
|  | Cases Used | Statistics are based on all cases with valid data. |
| Syntax | | FREQUENCIES VARIABLES=How many weeks did your last pregnancy last? How old are you? How many pregnancies did you have? How many miscarriages did you have? How many live births did you have? How many ANC visits did you have during your last pregnancy? When was your first ANC visit during your last pregnancy? If the answer is other kindly mention it Did you receive antenatal care during your last pregnancy?  Who was the healthcare provider during your pregnancy? If the answer is "Other," what is it? What type of healthcare services were provided?  Do you have health insurance?  What mode of transportation do you use to reach the maternity healthcare center? If the answer is "other," what is it?_A  How many visits did you make during your last pregnancy?  When was your first visit (in terms of weeks of pregnancy)  How does Antenatal care affect the health of the mother and the baby? Do you usually forget your appointment dates? Can you get the information you need about pregnancy from family or friends? Are maternity healthcare appointments usually too short and rushed? Are the working hours of the maternity care centre convenient for you? Are there long waiting times at the maternity care centre? Do you trust your healthcare provider? Do you think the people who monitor your pregnancy understand your health issues? Do you have someone to take care of your children while you go to the maternity care centre? Do you have other work commitments that take priority over Antenatal care? Does your husband have a negative view of Antenatal care services? Does your mother have a negative view of Antenatal care services? Do your peers have a negative view of Antenatal care services? Does attending the maternity care centre take up a lot of your time? Does attending the maternity care centre cost a lot of money? Is attending the maternity care centre physically exhausting? During visits to the care centres during your last pregnancy did they take your weight?  During visits to the care centres during your last pregnancy did they take your blood pressure?  During visits to the care centres during your last pregnancy did they make any urine tests?  During visits to the care centres during your last pregnancy did they make any blood test?  During visits to the care centres during your last pregnancy were you informed of the purpose of the tests being done?  During visits to the care centres during your last pregnancy did you understand the purpose of the drugs being prescribed to you?  During visits to the care centres during your last pregnancy did they inform you about the natural changes to expect during pregnancy (nausea, vomiting)?  During visits to the care centres during your last pregnancy did they inform you about the signs of complications in pregnancy (anaemia, seizures)?  During the visits to the care centres during your last pregnancy, were you informed where you should go in case of complications?  During the visits to the care centres during your last pregnancy, were you given instructions regarding preparation for labour?  During the visits to the care centres during your last pregnancy, were you given instructions about nutrition?  During the visits to the care centres during your last pregnancy, were you given instructions regarding breastfeeding?  During the visits to the care centres during your last pregnancy, did you feel comfortable asking all the questions you wanted?  During the visits to the care centres during your last pregnancy, were you able to discuss your concerns privately?  During the visits to the care centres during your last pregnancy, did you feel that the healthcare provider treated you with respect?  During the visits to the care centres during your last pregnancy, did you feel that the healthcare provider treated you differently for a personal reason? Did you feel that you were treated differently from others?  During the visits to the care centres during your last pregnancy, did you feel that the facility (maternity care centre) was clean?  Have you been circumcised (FGM)? WHO_recommendation_for_ANC_visits  /ORDER=ANALYSIS. |
| Resources | Processor Time | 00:00:00.05 |
|  | Elapsed Time | 00:00:00.05 |

| **Warnings** |
| --- |
| One or more values of variable Is there any other complications (mention them) contained a non-printing character. Each such character was replaced by a space. The data file itself was not modified. |

**Frequency Table**

| **How many weeks did your last pregnancy last?** | | | | | |
| --- | --- | --- | --- | --- | --- |
|  | | Frequency | Percent | Valid Percent | Cumulative Percent |
| Valid | 13 | 7 | 2.8 | 2.9 | 2.9 |
|  | 14 | 1 | .4 | .4 | 3.3 |
|  | 15 | 1 | .4 | .4 | 3.8 |
|  | 17 | 9 | 3.6 | 3.8 | 7.5 |
|  | 20 | 3 | 1.2 | 1.3 | 8.8 |
|  | 21 | 3 | 1.2 | 1.3 | 10.0 |
|  | 22 | 1 | .4 | .4 | 10.5 |
|  | 23 | 1 | .4 | .4 | 10.9 |
|  | 24 | 2 | .8 | .8 | 11.7 |
|  | 26 | 3 | 1.2 | 1.3 | 13.0 |
|  | 27 | 1 | .4 | .4 | 13.4 |
|  | 30 | 4 | 1.6 | 1.7 | 15.1 |
|  | 31 | 1 | .4 | .4 | 15.5 |
|  | 32 | 6 | 2.4 | 2.5 | 18.0 |
|  | 33 | 2 | .8 | .8 | 18.8 |
|  | 34 | 2 | .8 | .8 | 19.7 |
|  | 35 | 16 | 6.4 | 6.7 | 26.4 |
|  | 36 | 22 | 8.8 | 9.2 | 35.6 |
|  | 37 | 7 | 2.8 | 2.9 | 38.5 |
|  | 38 | 3 | 1.2 | 1.3 | 39.7 |
|  | 39 | 74 | 29.5 | 31.0 | 70.7 |
|  | 40 | 58 | 23.1 | 24.3 | 95.0 |
|  | 41 | 7 | 2.8 | 2.9 | 97.9 |
|  | 42 | 3 | 1.2 | 1.3 | 99.2 |
|  | 43 | 1 | .4 | .4 | 99.6 |
|  | 46 | 1 | .4 | .4 | 100.0 |
|  | Total | 239 | 95.2 | 100.0 |  |
| Missing | 99 | 12 | 4.8 |  |  |
| Total | | 251 | 100.0 |  |  |

| **When was the last pregnancy?** | | | | | |
| --- | --- | --- | --- | --- | --- |
|  | | Frequency | Percent | Valid Percent | Cumulative Percent |
| Valid | Less than an year | 144 | 57.4 | 58.1 | 58.1 |
|  | Less than two years | 104 | 41.4 | 41.9 | 100.0 |
|  | Total | 248 | 98.8 | 100.0 |  |
| Missing | 99 | 3 | 1.2 |  |  |
| Total | | 251 | 100.0 |  |  |

| **How old are you?** | | | | | |
| --- | --- | --- | --- | --- | --- |
|  | | Frequency | Percent | Valid Percent | Cumulative Percent |
| Valid | 18 | 8 | 3.2 | 3.2 | 3.2 |
|  | 19 | 11 | 4.4 | 4.4 | 7.7 |
|  | 20 | 19 | 7.6 | 7.7 | 15.3 |
|  | 21 | 17 | 6.8 | 6.9 | 22.2 |
|  | 22 | 21 | 8.4 | 8.5 | 30.6 |
|  | 23 | 24 | 9.6 | 9.7 | 40.3 |
|  | 24 | 11 | 4.4 | 4.4 | 44.8 |
|  | 25 | 24 | 9.6 | 9.7 | 54.4 |
|  | 26 | 14 | 5.6 | 5.6 | 60.1 |
|  | 27 | 20 | 8.0 | 8.1 | 68.1 |
|  | 28 | 12 | 4.8 | 4.8 | 73.0 |
|  | 29 | 4 | 1.6 | 1.6 | 74.6 |
|  | 30 | 19 | 7.6 | 7.7 | 82.3 |
|  | 31 | 3 | 1.2 | 1.2 | 83.5 |
|  | 32 | 4 | 1.6 | 1.6 | 85.1 |
|  | 33 | 3 | 1.2 | 1.2 | 86.3 |
|  | 34 | 3 | 1.2 | 1.2 | 87.5 |
|  | 35 | 17 | 6.8 | 6.9 | 94.4 |
|  | 36 | 2 | .8 | .8 | 95.2 |
|  | 37 | 2 | .8 | .8 | 96.0 |
|  | 38 | 5 | 2.0 | 2.0 | 98.0 |
|  | 39 | 1 | .4 | .4 | 98.4 |
|  | 40 | 3 | 1.2 | 1.2 | 99.6 |
|  | 45 | 1 | .4 | .4 | 100.0 |
|  | Total | 248 | 98.8 | 100.0 |  |
| Missing | 99 | 3 | 1.2 |  |  |
| Total | | 251 | 100.0 |  |  |

| **What is your marital status?** | | | | | |
| --- | --- | --- | --- | --- | --- |
|  | | Frequency | Percent | Valid Percent | Cumulative Percent |
| Valid | separated | 2 | .8 | .8 | .8 |
|  | Married | 249 | 99.2 | 99.2 | 100.0 |
|  | Total | 251 | 100.0 | 100.0 |  |

| **What is your educational level?** | | | | | |
| --- | --- | --- | --- | --- | --- |
|  | | Frequency | Percent | Valid Percent | Cumulative Percent |
| Valid | illiterate | 4 | 1.6 | 1.6 | 1.6 |
|  | primary school | 99 | 39.4 | 39.6 | 41.2 |
|  | middle school | 60 | 23.9 | 24.0 | 65.2 |
|  | secondary school | 82 | 32.7 | 32.8 | 98.0 |
|  | bachelor degree | 5 | 2.0 | 2.0 | 100.0 |
|  | Total | 250 | 99.6 | 100.0 |  |
| Missing | 99 | 1 | .4 |  |  |
| Total | | 251 | 100.0 |  |  |

| **What is your husband's educational level?** | | | | | |
| --- | --- | --- | --- | --- | --- |
|  | | Frequency | Percent | Valid Percent | Cumulative Percent |
| Valid | illiterate | 7 | 2.8 | 2.8 | 2.8 |
|  | primary school | 94 | 37.5 | 37.6 | 40.4 |
|  | middle school | 56 | 22.3 | 22.4 | 62.8 |
|  | secondary school | 66 | 26.3 | 26.4 | 89.2 |
|  | bachelor | 14 | 5.6 | 5.6 | 94.8 |
|  | diploma | 6 | 2.4 | 2.4 | 97.2 |
|  | (Khalwa) | 6 | 2.4 | 2.4 | 99.6 |
|  | others | 1 | .4 | .4 | 100.0 |
|  | Total | 250 | 99.6 | 100.0 |  |
| Missing | 99 | 1 | .4 |  |  |
| Total | | 251 | 100.0 |  |  |

| **What is your occupation?** | | | | | |
| --- | --- | --- | --- | --- | --- |
|  | | Frequency | Percent | Valid Percent | Cumulative Percent |
| Valid | housewife | 229 | 91.2 | 91.2 | 91.2 |
|  | Free work | 14 | 5.6 | 5.6 | 96.8 |
|  | other | 8 | 3.2 | 3.2 | 100.0 |
|  | Total | 251 | 100.0 | 100.0 |  |

| **What is your husband's occupation?** | | | | | |
| --- | --- | --- | --- | --- | --- |
|  | | Frequency | Percent | Valid Percent | Cumulative Percent |
| Valid | I do not work | 5 | 2.0 | 2.0 | 2.0 |
|  | free work | 235 | 93.6 | 93.6 | 95.6 |
|  | other | 4 | 1.6 | 1.6 | 97.2 |
|  | student | 1 | .4 | .4 | 97.6 |
|  | governmental employee | 3 | 1.2 | 1.2 | 98.8 |
|  | privite sector employee | 3 | 1.2 | 1.2 | 100.0 |
|  | Total | 251 | 100.0 | 100.0 |  |

| **How many times have you been pregnant?** | | | | | |
| --- | --- | --- | --- | --- | --- |
|  | | Frequency | Percent | Valid Percent | Cumulative Percent |
| Valid | 1 | 24 | 9.6 | 9.6 | 9.6 |
|  | 2 | 29 | 11.6 | 11.6 | 21.1 |
|  | 3 | 39 | 15.5 | 15.5 | 36.7 |
|  | 4 | 30 | 12.0 | 12.0 | 48.6 |
|  | 5 | 32 | 12.7 | 12.7 | 61.4 |
|  | 6 | 34 | 13.5 | 13.5 | 74.9 |
|  | 7 | 21 | 8.4 | 8.4 | 83.3 |
|  | 8 | 21 | 8.4 | 8.4 | 91.6 |
|  | 9 | 9 | 3.6 | 3.6 | 95.2 |
|  | 10 | 5 | 2.0 | 2.0 | 97.2 |
|  | 11 | 4 | 1.6 | 1.6 | 98.8 |
|  | 12 | 1 | .4 | .4 | 99.2 |
|  | 13 | 2 | .8 | .8 | 100.0 |
|  | Total | 251 | 100.0 | 100.0 |  |

| **How many times have you had a miscarriage?** | | | | | |
| --- | --- | --- | --- | --- | --- |
|  | | Frequency | Percent | Valid Percent | Cumulative Percent |
| Valid | 0 | 156 | 62.2 | 62.2 | 62.2 |
|  | 1 | 54 | 21.5 | 21.5 | 83.7 |
|  | 2 | 30 | 12.0 | 12.0 | 95.6 |
|  | 3 | 7 | 2.8 | 2.8 | 98.4 |
|  | 4 | 4 | 1.6 | 1.6 | 100.0 |
|  | Total | 251 | 100.0 | 100.0 |  |

| **How many children have you given birth to alive?** | | | | | |
| --- | --- | --- | --- | --- | --- |
|  | | Frequency | Percent | Valid Percent | Cumulative Percent |
| Valid | 0 | 9 | 3.6 | 3.6 | 3.6 |
|  | 1 | 29 | 11.6 | 11.7 | 15.4 |
|  | 2 | 41 | 16.3 | 16.6 | 32.0 |
|  | 3 | 32 | 12.7 | 13.0 | 44.9 |
|  | 4 | 31 | 12.4 | 12.6 | 57.5 |
|  | 5 | 30 | 12.0 | 12.1 | 69.6 |
|  | 6 | 39 | 15.5 | 15.8 | 85.4 |
|  | 7 | 15 | 6.0 | 6.1 | 91.5 |
|  | 8 | 10 | 4.0 | 4.0 | 95.5 |
|  | 9 | 6 | 2.4 | 2.4 | 98.0 |
|  | 10 | 2 | .8 | .8 | 98.8 |
|  | 11 | 3 | 1.2 | 1.2 | 100.0 |
|  | Total | 247 | 98.4 | 100.0 |  |
| Missing | 99 | 4 | 1.6 |  |  |
| Total | | 251 | 100.0 |  |  |

| **During your last pregnancy, did you experience anemia?** | | | | | |
| --- | --- | --- | --- | --- | --- |
|  | | Frequency | Percent | Valid Percent | Cumulative Percent |
| Valid | no | 228 | 90.8 | 90.8 | 90.8 |
|  | yes | 23 | 9.2 | 9.2 | 100.0 |
|  | Total | 251 | 100.0 | 100.0 |  |

| **During your last pregnancy, did you experience cramps?** | | | | | |
| --- | --- | --- | --- | --- | --- |
|  | | Frequency | Percent | Valid Percent | Cumulative Percent |
| Valid | no | 243 | 96.8 | 97.2 | 97.2 |
|  | yes | 7 | 2.8 | 2.8 | 100.0 |
|  | Total | 250 | 99.6 | 100.0 |  |
| Missing | 99 | 1 | .4 |  |  |
| Total | | 251 | 100.0 |  |  |

| **During your last pregnancy, did you experience thyroid diseases or their complications?** | | | | | |
| --- | --- | --- | --- | --- | --- |
|  | | Frequency | Percent | Valid Percent | Cumulative Percent |
| Valid | no | 240 | 95.6 | 96.0 | 96.0 |
|  | yes | 10 | 4.0 | 4.0 | 100.0 |
|  | Total | 250 | 99.6 | 100.0 |  |
| Missing | 99 | 1 | .4 |  |  |
| Total | | 251 | 100.0 |  |  |

| **During your last pregnancy, did you experience preterm labor or incomplete delivery?** | | | | | |
| --- | --- | --- | --- | --- | --- |
|  | | Frequency | Percent | Valid Percent | Cumulative Percent |
| Valid | no | 228 | 90.8 | 90.8 | 90.8 |
|  | yes | 23 | 9.2 | 9.2 | 100.0 |
|  | Total | 251 | 100.0 | 100.0 |  |

| **During your last pregnancy, did you experience obstructed or difficult labor?** | | | | | |
| --- | --- | --- | --- | --- | --- |
|  | | Frequency | Percent | Valid Percent | Cumulative Percent |
| Valid | no | 194 | 77.3 | 77.6 | 77.6 |
|  | yes | 56 | 22.3 | 22.4 | 100.0 |
|  | Total | 250 | 99.6 | 100.0 |  |
| Missing | 99 | 1 | .4 |  |  |
| Total | | 251 | 100.0 |  |  |

| **During your last pregnancy, did you experience shoulder dystocia?** | | | | | |
| --- | --- | --- | --- | --- | --- |
|  | | Frequency | Percent | Valid Percent | Cumulative Percent |
| Valid | no | 230 | 91.6 | 92.0 | 92.0 |
|  | yes | 20 | 8.0 | 8.0 | 100.0 |
|  | Total | 250 | 99.6 | 100.0 |  |
| Missing | 99 | 1 | .4 |  |  |
| Total | | 251 | 100.0 |  |  |

| **During your last pregnancy, did you experience a large baby weighing ≥ 4.5 kg?** | | | | | |
| --- | --- | --- | --- | --- | --- |
|  | | Frequency | Percent | Valid Percent | Cumulative Percent |
| Valid | no | 228 | 90.8 | 91.6 | 91.6 |
|  | yes | 21 | 8.4 | 8.4 | 100.0 |
|  | Total | 249 | 99.2 | 100.0 |  |
| Missing | 99 | 2 | .8 |  |  |
| Total | | 251 | 100.0 |  |  |

| **During your last pregnancy, did you experience postpartum hemorrhage?** | | | | | |
| --- | --- | --- | --- | --- | --- |
|  | | Frequency | Percent | Valid Percent | Cumulative Percent |
| Valid | no | 238 | 94.8 | 95.6 | 95.6 |
|  | yes | 11 | 4.4 | 4.4 | 100.0 |
|  | Total | 249 | 99.2 | 100.0 |  |
| Missing | 99 | 2 | .8 |  |  |
| Total | | 251 | 100.0 |  |  |

| **During your last pregnancy, did you experience intrauterine growth restriction (IUGR) or halted fetal growth?** | | | | | |
| --- | --- | --- | --- | --- | --- |
|  | | Frequency | Percent | Valid Percent | Cumulative Percent |
| Valid | no | 243 | 96.8 | 97.2 | 97.2 |
|  | yes | 7 | 2.8 | 2.8 | 100.0 |
|  | Total | 250 | 99.6 | 100.0 |  |
| Missing | 99 | 1 | .4 |  |  |
| Total | | 251 | 100.0 |  |  |

| **During your last pregnancy, did you experience intrauterine fetal death?** | | | | | |
| --- | --- | --- | --- | --- | --- |
|  | | Frequency | Percent | Valid Percent | Cumulative Percent |
| Valid | no | 232 | 92.4 | 92.8 | 92.8 |
|  | yes | 18 | 7.2 | 7.2 | 100.0 |
|  | Total | 250 | 99.6 | 100.0 |  |
| Missing | 99 | 1 | .4 |  |  |
| Total | | 251 | 100.0 |  |  |

| **Did you experience any other complications?** | | | | | |
| --- | --- | --- | --- | --- | --- |
|  | | Frequency | Percent | Valid Percent | Cumulative Percent |
| Valid | back pain | 1 | .4 | .4 | .4 |
|  | Destrup رعشه في اليد Cheast pain | 1 | .4 | .4 | .8 |
|  | Dfustion after birth form uters | 1 | .4 | .4 | 1.2 |
|  | URTI | 1 | .4 | .4 | 1.6 |
|  | High heart beat, Fever, and headache | 1 | .4 | .4 | 2.0 |
|  | Tiredness | 1 | .4 | .4 | 2.4 |
|  | Tiredness during pregnancy | 1 | .4 | .4 | 2.8 |
|  | Vomiting and Malaria | 1 | .4 | .4 | 3.2 |
|  | Depression after pregnancy | 1 | .4 | .4 | 3.6 |
|  | Tiredness | 1 | .4 | .4 | 4.0 |
|  | Urethrities | 1 | .4 | .4 | 4.4 |
|  | inflammations | 1 | .4 | .4 | 4.8 |
|  | Yellowish baby | 1 | .4 | .4 | 5.2 |
|  | Seperated placenta | 1 | .4 | .4 | 5.6 |
|  | White foot | 1 | .4 | .4 | 6.0 |
|  | Pain in Vagina | 1 | .4 | .4 | 6.4 |
|  | Bleeding after delivery | 1 | .4 | .4 | 6.8 |
|  | Unnormal delivery due to narrow pelvic | 1 | .4 | .4 | 7.2 |
|  | Menopause After delivery | 1 | .4 | .4 | 7.6 |
|  | Blood clot | 1 | .4 | .4 | 8.0 |
|  | Tiredness | 1 | .4 | .4 | 8.4 |
|  | Tiredness and pain | 1 | .4 | .4 | 8.8 |
|  | Envoleping | 1 | .4 | .4 | 9.2 |
|  | gastritis | 1 | .4 | .4 | 9.6 |
|  | allergy | 1 | .4 | .4 | 10.0 |
|  | Flow in urine | 1 | .4 | .4 | 10.4 |
|  | headache | 1 | .4 | .4 | 10.8 |
|  | Difficulty in breathing and convulsions | 1 | .4 | .4 | 11.2 |
|  | Can not see in night | 1 | .4 | .4 | 11.6 |
|  | Tiredness | 1 | .4 | .4 | 12.0 |
|  | In the late month high heartbeats and Tiredness. Back pain and foot pain and continued headache even after delivery, there were many secretions | 1 | .4 | .4 | 12.4 |
|  | Pre-delivery in all children | 1 | .4 | .4 | 12.7 |
|  | In the third month bleeding and bagging | 1 | .4 | .4 | 13.1 |
|  | No | 204 | 81.3 | 81.3 | 94.4 |
|  | Spinning and headache | 1 | .4 | .4 | 94.8 |
|  | malaria | 4 | 1.6 | 1.6 | 96.4 |
|  | Excess water and miscarriage | 1 | .4 | .4 | 96.8 |
|  | Water in the uterus | 1 | .4 | .4 | 97.2 |
|  | bleeding | 1 | .4 | .4 | 97.6 |
|  | Weight loss | 1 | .4 | .4 | 98.0 |
|  | drop | 3 | 1.2 | 1.2 | 99.2 |
|  | Drop and tiredness during breastfeeding | 1 | .4 | .4 | 99.6 |
|  | Jaundice and pressure problem | 1 | .4 | .4 | 100.0 |
|  | Total | 251 | 100.0 | 100.0 |  |

| **Did you receive antenatal care during your last pregnancy?** | | | | | |
| --- | --- | --- | --- | --- | --- |
|  | | Frequency | Percent | Valid Percent | Cumulative Percent |
| Valid | no | 22 | 8.8 | 8.8 | 8.8 |
|  | yes | 229 | 91.2 | 91.2 | 100.0 |
|  | Total | 251 | 100.0 | 100.0 |  |

| **If the answer is "Other," what is it?** | | | | | |
| --- | --- | --- | --- | --- | --- |
|  | | Frequency | Percent | Valid Percent | Cumulative Percent |
| Valid | 5 | 25 | 10.0 | 75.8 | 75.8 |
|  | specialist | 1 | .4 | 3.0 | 78.8 |
|  | Elmanagel locality | 4 | 1.6 | 12.1 | 90.9 |
|  | center | 3 | 1.2 | 9.1 | 100.0 |
|  | Total | 33 | 13.1 | 100.0 |  |
| Missing | 99 | 196 | 78.1 |  |  |
|  | 999 | 22 | 8.8 |  |  |
|  | Total | 218 | 86.9 |  |  |
| Total | | 251 | 100.0 |  |  |

| **What type of healthcare services were provided?** | | | | | |
| --- | --- | --- | --- | --- | --- |
|  | | Frequency | Percent | Valid Percent | Cumulative Percent |
| Valid | private | 156 | 62.2 | 68.4 | 68.4 |
|  | public | 72 | 28.7 | 31.6 | 100.0 |
|  | Total | 228 | 90.8 | 100.0 |  |
| Missing | 99 | 1 | .4 |  |  |
|  | 999 | 22 | 8.8 |  |  |
|  | Total | 23 | 9.2 |  |  |
| Total | | 251 | 100.0 |  |  |

| **Do you have health insurance?** | | | | | |
| --- | --- | --- | --- | --- | --- |
|  | | Frequency | Percent | Valid Percent | Cumulative Percent |
| Valid | no | 213 | 84.9 | 84.9 | 84.9 |
|  | yes | 37 | 14.7 | 14.7 | 99.6 |
|  | I do not know | 1 | .4 | .4 | 100.0 |
|  | Total | 251 | 100.0 | 100.0 |  |

| **If the answer is "other," what is it?** | | | |
| --- | --- | --- | --- |
|  | | Frequency | Percent |
| Missing | 99 | 229 | 91.2 |
|  | 999 | 22 | 8.8 |
|  | Total | 251 | 100.0 |

| **How many ANC visits did you have during your last pregnancy?** | | | | | |
| --- | --- | --- | --- | --- | --- |
|  | | Frequency | Percent | Valid Percent | Cumulative Percent |
| Valid | 0 | 143 | 57.0 | 62.4 | 62.4 |
|  | 1 | 86 | 34.3 | 37.6 | 100.0 |
|  | Total | 229 | 91.2 | 100.0 |  |
| Missing | 999 | 22 | 8.8 |  |  |
| Total | | 251 | 100.0 |  |  |

| **When was your first ANC visit during your last pregnancy?** | | | | | |
| --- | --- | --- | --- | --- | --- |
|  | | Frequency | Percent | Valid Percent | Cumulative Percent |
| Valid | 0 | 3 | 1.2 | 1.3 | 1.3 |
|  | 2 | 2 | .8 | .9 | 2.2 |
|  | 3 | 3 | 1.2 | 1.3 | 3.6 |
|  | 4 | 10 | 4.0 | 4.4 | 8.0 |
|  | 5 | 11 | 4.4 | 4.9 | 12.9 |
|  | 6 | 8 | 3.2 | 3.6 | 16.4 |
|  | 7 | 6 | 2.4 | 2.7 | 19.1 |
|  | 8 | 31 | 12.4 | 13.8 | 32.9 |
|  | 9 | 7 | 2.8 | 3.1 | 36.0 |
|  | 10 | 1 | .4 | .4 | 36.4 |
|  | 12 | 8 | 3.2 | 3.6 | 40.0 |
|  | 13 | 28 | 11.2 | 12.4 | 52.4 |
|  | 14 | 2 | .8 | .9 | 53.3 |
|  | 15 | 2 | .8 | .9 | 54.2 |
|  | 16 | 4 | 1.6 | 1.8 | 56.0 |
|  | 17 | 33 | 13.1 | 14.7 | 70.7 |
|  | 18 | 3 | 1.2 | 1.3 | 72.0 |
|  | 19 | 1 | .4 | .4 | 72.4 |
|  | 20 | 5 | 2.0 | 2.2 | 74.7 |
|  | 21 | 3 | 1.2 | 1.3 | 76.0 |
|  | 22 | 11 | 4.4 | 4.9 | 80.9 |
|  | 24 | 2 | .8 | .9 | 81.8 |
|  | 25 | 2 | .8 | .9 | 82.7 |
|  | 26 | 10 | 4.0 | 4.4 | 87.1 |
|  | 27 | 1 | .4 | .4 | 87.6 |
|  | 28 | 4 | 1.6 | 1.8 | 89.3 |
|  | 29 | 1 | .4 | .4 | 89.8 |
|  | 30 | 12 | 4.8 | 5.3 | 95.1 |
|  | 32 | 1 | .4 | .4 | 95.6 |
|  | 33 | 1 | .4 | .4 | 96.0 |
|  | 34 | 1 | .4 | .4 | 96.4 |
|  | 35 | 3 | 1.2 | 1.3 | 97.8 |
|  | 37 | 1 | .4 | .4 | 98.2 |
|  | 39 | 1 | .4 | .4 | 98.7 |
|  | 43 | 2 | .8 | .9 | 99.6 |
|  | 69 | 1 | .4 | .4 | 100.0 |
|  | Total | 225 | 89.6 | 100.0 |  |
| Missing | 99 | 4 | 1.6 |  |  |
|  | 999 | 22 | 8.8 |  |  |
|  | Total | 26 | 10.4 |  |  |
| Total | | 251 | 100.0 |  |  |

| **How does Antenatal care affect the health of the mother and the baby?** | | | | | |
| --- | --- | --- | --- | --- | --- |
|  | | Frequency | Percent | Valid Percent | Cumulative Percent |
| Valid | does not affect | 9 | 3.6 | 3.6 | 3.6 |
|  | positivly affect | 236 | 94.0 | 94.4 | 98.0 |
|  | negetivly affect | 3 | 1.2 | 1.2 | 99.2 |
|  | I do not know | 2 | .8 | .8 | 100.0 |
|  | Total | 250 | 99.6 | 100.0 |  |
| Missing | 99 | 1 | .4 |  |  |
| Total | | 251 | 100.0 |  |  |

| **How does Antenatal care affect the health of the mother and the baby?** | | | | | |
| --- | --- | --- | --- | --- | --- |
|  | | Frequency | Percent | Valid Percent | Cumulative Percent |
| Valid | I do not know‎/ i do not remember | 6 | 2.4 | 2.4 | 2.4 |
|  | NO | 175 | 69.7 | 70.0 | 72.4 |
|  | YES | 52 | 20.7 | 20.8 | 93.2 |
|  | neutral | 11 | 4.4 | 4.4 | 97.6 |
|  | I do not know‎ there is a fixed visits schedule | 6 | 2.4 | 2.4 | 100.0 |
|  | Total | 250 | 99.6 | 100.0 |  |
| Missing | 99 | 1 | .4 |  |  |
| Total | | 251 | 100.0 |  |  |

| **Can you get the information you need about pregnancy from family or friends?** | | | | | |
| --- | --- | --- | --- | --- | --- |
|  | | Frequency | Percent | Valid Percent | Cumulative Percent |
| Valid | I do not know‎ | 2 | .8 | .8 | .8 |
|  | NO | 110 | 43.8 | 43.8 | 44.6 |
|  | YES | 113 | 45.0 | 45.0 | 89.6 |
|  | neutral | 26 | 10.4 | 10.4 | 100.0 |
|  | Total | 251 | 100.0 | 100.0 |  |

| **Are maternity healthcare appointments usually too short and rushed?** | | | | | |
| --- | --- | --- | --- | --- | --- |
|  | | Frequency | Percent | Valid Percent | Cumulative Percent |
| Valid | I do not know‎ | 6 | 2.4 | 2.4 | 2.4 |
|  | NO | 90 | 35.9 | 35.9 | 38.2 |
|  | YES | 106 | 42.2 | 42.2 | 80.5 |
|  | neutral | 49 | 19.5 | 19.5 | 100.0 |
|  | Total | 251 | 100.0 | 100.0 |  |

| **Are the working hours of the maternity care centre convenient for you?** | | | | | |
| --- | --- | --- | --- | --- | --- |
|  | | Frequency | Percent | Valid Percent | Cumulative Percent |
| Valid | I do not know‎ | 9 | 3.6 | 3.6 | 3.6 |
|  | NO | 21 | 8.4 | 8.4 | 12.0 |
|  | YES | 208 | 82.9 | 83.5 | 95.6 |
|  | neutral | 11 | 4.4 | 4.4 | 100.0 |
|  | Total | 249 | 99.2 | 100.0 |  |
| Missing | 99 | 2 | .8 |  |  |
| Total | | 251 | 100.0 |  |  |

| **Are there long waiting times at the maternity care centre?** | | | | | |
| --- | --- | --- | --- | --- | --- |
|  | | Frequency | Percent | Valid Percent | Cumulative Percent |
| Valid | I do not know‎ | 7 | 2.8 | 2.8 | 2.8 |
|  | NO | 60 | 23.9 | 24.0 | 26.8 |
|  | YES | 144 | 57.4 | 57.6 | 84.4 |
|  | neutral | 39 | 15.5 | 15.6 | 100.0 |
|  | Total | 250 | 99.6 | 100.0 |  |
| Missing | 99 | 1 | .4 |  |  |
| Total | | 251 | 100.0 |  |  |

| **Do you trust your healthcare provider?** | | | | | |
| --- | --- | --- | --- | --- | --- |
|  | | Frequency | Percent | Valid Percent | Cumulative Percent |
| Valid | I do not know‎ | 6 | 2.4 | 2.4 | 2.4 |
|  | NO | 7 | 2.8 | 2.8 | 5.2 |
|  | YES | 229 | 91.2 | 92.0 | 97.2 |
|  | neutral | 7 | 2.8 | 2.8 | 100.0 |
|  | Total | 249 | 99.2 | 100.0 |  |
| Missing | 99 | 2 | .8 |  |  |
| Total | | 251 | 100.0 |  |  |

| **Do you think that the people who provide maternity care understand your health issues?** | | | | | |
| --- | --- | --- | --- | --- | --- |
|  | | Frequency | Percent | Valid Percent | Cumulative Percent |
| Valid | I do not know‎ | 7 | 2.8 | 2.8 | 2.8 |
|  | NO | 6 | 2.4 | 2.4 | 5.2 |
|  | YES | 227 | 90.4 | 91.2 | 96.4 |
|  | neutral | 9 | 3.6 | 3.6 | 100.0 |
|  | Total | 249 | 99.2 | 100.0 |  |
| Missing | 99 | 2 | .8 |  |  |
| Total | | 251 | 100.0 |  |  |

| **Do you have someone to take care of your children while you go to the maternity care centre?** | | | | | |
| --- | --- | --- | --- | --- | --- |
|  | | Frequency | Percent | Valid Percent | Cumulative Percent |
| Valid | I do not have kids | 20 | 8.0 | 8.0 | 8.0 |
|  | NO | 11 | 4.4 | 4.4 | 12.4 |
|  | YES | 211 | 84.1 | 84.7 | 97.2 |
|  | neutral | 7 | 2.8 | 2.8 | 100.0 |
|  | Total | 249 | 99.2 | 100.0 |  |
| Missing | 99 | 2 | .8 |  |  |
| Total | | 251 | 100.0 |  |  |

| ‫**Do you have other work commitments that take priority over Antenatal care?** | | | | | |
| --- | --- | --- | --- | --- | --- |
|  | | Frequency | Percent | Valid Percent | Cumulative Percent |
| Valid | I do not know‎ | 4 | 1.6 | 1.6 | 1.6 |
|  | NO | 198 | 78.9 | 79.5 | 81.1 |
|  | YES | 37 | 14.7 | 14.9 | 96.0 |
|  | neutral | 10 | 4.0 | 4.0 | 100.0 |
|  | Total | 249 | 99.2 | 100.0 |  |
| Missing | 99 | 2 | .8 |  |  |
| Total | | 251 | 100.0 |  |  |

| **Does your husband have a negative view of Antenatal care services?** | | | | | |
| --- | --- | --- | --- | --- | --- |
|  | | Frequency | Percent | Valid Percent | Cumulative Percent |
| Valid | I do not know‎ | 1 | .4 | .4 | .4 |
|  | NO | 225 | 89.6 | 89.6 | 90.0 |
|  | YES | 21 | 8.4 | 8.4 | 98.4 |
|  | neutral | 4 | 1.6 | 1.6 | 100.0 |
|  | Total | 251 | 100.0 | 100.0 |  |

| **Does your mother have a negative view of Antenatal care services?** | | | | | |
| --- | --- | --- | --- | --- | --- |
|  | | Frequency | Percent | Valid Percent | Cumulative Percent |
| Valid | I do not know‎ | 4 | 1.6 | 1.6 | 1.6 |
|  | NO | 226 | 90.0 | 90.4 | 92.0 |
|  | YES | 17 | 6.8 | 6.8 | 98.8 |
|  | neutral | 3 | 1.2 | 1.2 | 100.0 |
|  | Total | 250 | 99.6 | 100.0 |  |
| Missing | 99 | 1 | .4 |  |  |
| Total | | 251 | 100.0 |  |  |

| **Do your peers have a negative view of Antenatal care services?** | | | | | |
| --- | --- | --- | --- | --- | --- |
|  | | Frequency | Percent | Valid Percent | Cumulative Percent |
| Valid | I do not know‎ | 1 | .4 | .4 | .4 |
|  | NO | 213 | 84.9 | 85.2 | 85.6 |
|  | YES | 32 | 12.7 | 12.8 | 98.4 |
|  | neutral | 4 | 1.6 | 1.6 | 100.0 |
|  | Total | 250 | 99.6 | 100.0 |  |
| Missing | 99 | 1 | .4 |  |  |
| Total | | 251 | 100.0 |  |  |

| **Does attending the maternity care centre take up a lot of your time?** | | | | | |
| --- | --- | --- | --- | --- | --- |
|  | | Frequency | Percent | Valid Percent | Cumulative Percent |
| Valid | I do not know‎ | 6 | 2.4 | 2.4 | 2.4 |
|  | NO | 128 | 51.0 | 51.2 | 53.6 |
|  | YES | 98 | 39.0 | 39.2 | 92.8 |
|  | neutral | 18 | 7.2 | 7.2 | 100.0 |
|  | Total | 250 | 99.6 | 100.0 |  |
| Missing | 99 | 1 | .4 |  |  |
| Total | | 251 | 100.0 |  |  |

| **Does attending the maternity care centre cost a lot of money?** | | | | | |
| --- | --- | --- | --- | --- | --- |
|  | | Frequency | Percent | Valid Percent | Cumulative Percent |
| Valid | I do not know‎ | 6 | 2.4 | 2.4 | 2.4 |
|  | NO | 108 | 43.0 | 43.2 | 45.6 |
|  | YES | 99 | 39.4 | 39.6 | 85.2 |
|  | neutral | 37 | 14.7 | 14.8 | 100.0 |
|  | Total | 250 | 99.6 | 100.0 |  |
| Missing | 99 | 1 | .4 |  |  |
| Total | | 251 | 100.0 |  |  |

| **Is attending the maternity care centre physically exhausting?‬** | | | | | |
| --- | --- | --- | --- | --- | --- |
|  | | Frequency | Percent | Valid Percent | Cumulative Percent |
| Valid | I do not know‎ | 7 | 2.8 | 2.8 | 2.8 |
|  | NO | 140 | 55.8 | 56.0 | 58.8 |
|  | YES | 93 | 37.1 | 37.2 | 96.0 |
|  | neutral | 10 | 4.0 | 4.0 | 100.0 |
|  | Total | 250 | 99.6 | 100.0 |  |
| Missing | 99 | 1 | .4 |  |  |
| Total | | 251 | 100.0 |  |  |

| **During the visits to care centers during your last pregnancy, was your weight measured?** | | | | | |
| --- | --- | --- | --- | --- | --- |
|  | | Frequency | Percent | Valid Percent | Cumulative Percent |
| Valid | I do not know‎/ I do not remember | 4 | 1.6 | 1.8 | 1.8 |
|  | Never | 180 | 71.7 | 78.9 | 80.7 |
|  | few times | 13 | 5.2 | 5.7 | 86.4 |
|  | most times | 4 | 1.6 | 1.8 | 88.2 |
|  | every times | 27 | 10.8 | 11.8 | 100.0 |
|  | Total | 228 | 90.8 | 100.0 |  |
| Missing | 99 | 1 | .4 |  |  |
|  | 999 | 22 | 8.8 |  |  |
|  | Total | 23 | 9.2 |  |  |
| Total | | 251 | 100.0 |  |  |

| **During the visits to care centers during your last pregnancy, was your blood pressure measured?** | | | | | |
| --- | --- | --- | --- | --- | --- |
|  | | Frequency | Percent | Valid Percent | Cumulative Percent |
| Valid | I do not know‎/ I do not remember | 2 | .8 | .9 | .9 |
|  | Never | 8 | 3.2 | 3.5 | 4.4 |
|  | few times | 16 | 6.4 | 7.0 | 11.4 |
|  | most times | 17 | 6.8 | 7.5 | 18.9 |
|  | every times | 185 | 73.7 | 81.1 | 100.0 |
|  | Total | 228 | 90.8 | 100.0 |  |
| Missing | 99 | 1 | .4 |  |  |
|  | 999 | 22 | 8.8 |  |  |
|  | Total | 23 | 9.2 |  |  |
| Total | | 251 | 100.0 |  |  |

| **During the visits to care centers during your last pregnancy, was a urine test conducted?** | | | | | |
| --- | --- | --- | --- | --- | --- |
|  | | Frequency | Percent | Valid Percent | Cumulative Percent |
| Valid | I do not know‎/ I do not remember | 2 | .8 | .9 | .9 |
|  | Never | 8 | 3.2 | 3.5 | 4.4 |
|  | few times | 19 | 7.6 | 8.3 | 12.7 |
|  | most times | 18 | 7.2 | 7.9 | 20.6 |
|  | every times | 181 | 72.1 | 79.4 | 100.0 |
|  | Total | 228 | 90.8 | 100.0 |  |
| Missing | 99 | 1 | .4 |  |  |
|  | 999 | 22 | 8.8 |  |  |
|  | Total | 23 | 9.2 |  |  |
| Total | | 251 | 100.0 |  |  |

| **During the visits to care centers during your last pregnancy, was a blood test conducted?** | | | | | |
| --- | --- | --- | --- | --- | --- |
|  | | Frequency | Percent | Valid Percent | Cumulative Percent |
| Valid | I do not know‎/ I do not remember | 2 | .8 | .9 | .9 |
|  | Never | 6 | 2.4 | 2.6 | 3.5 |
|  | few times | 17 | 6.8 | 7.5 | 11.0 |
|  | most times | 19 | 7.6 | 8.4 | 19.4 |
|  | every times | 183 | 72.9 | 80.6 | 100.0 |
|  | Total | 227 | 90.4 | 100.0 |  |
| Missing | 99 | 2 | .8 |  |  |
|  | 999 | 22 | 8.8 |  |  |
|  | Total | 24 | 9.6 |  |  |
| Total | | 251 | 100.0 |  |  |

| **During the visits to care centers during your last pregnancy, did you understand the purpose of the tests being conducted on you?‏‫** | | | | | |
| --- | --- | --- | --- | --- | --- |
|  | | Frequency | Percent | Valid Percent | Cumulative Percent |
| Valid | I do not know‎/ I do not remember | 6 | 2.4 | 2.6 | 2.6 |
|  | Never | 69 | 27.5 | 30.4 | 33.0 |
|  | few times | 29 | 11.6 | 12.8 | 45.8 |
|  | most times | 17 | 6.8 | 7.5 | 53.3 |
|  | every times | 105 | 41.8 | 46.3 | 99.6 |
|  | no lab test was performed | 1 | .4 | .4 | 100.0 |
|  | Total | 227 | 90.4 | 100.0 |  |
| Missing | 99 | 2 | .8 |  |  |
|  | 999 | 22 | 8.8 |  |  |
|  | Total | 24 | 9.6 |  |  |
| Total | | 251 | 100.0 |  |  |

| **During your last pregnancy, did you understand the purpose of the medications given to you?** | | | | | |
| --- | --- | --- | --- | --- | --- |
|  | | Frequency | Percent | Valid Percent | Cumulative Percent |
| Valid | I do not know‎/ I do not remember | 3 | 1.2 | 1.3 | 1.3 |
|  | Never | 31 | 12.4 | 13.6 | 14.9 |
|  | few times | 19 | 7.6 | 8.3 | 23.2 |
|  | most times | 20 | 8.0 | 8.8 | 32.0 |
|  | every times | 152 | 60.6 | 66.7 | 98.7 |
|  | no medications were prescribed | 3 | 1.2 | 1.3 | 100.0 |
|  | Total | 228 | 90.8 | 100.0 |  |
| Missing | 99 | 1 | .4 |  |  |
|  | 999 | 22 | 8.8 |  |  |
|  | Total | 23 | 9.2 |  |  |
| Total | | 251 | 100.0 |  |  |

| **During the visits to care centers during your last pregnancy, were you informed about the natural changes during pregnancy and childbirth, such as nausea, vomiting, etc.?** | | | | | |
| --- | --- | --- | --- | --- | --- |
|  | | Frequency | Percent | Valid Percent | Cumulative Percent |
| Valid | I do not know‎ | 3 | 1.2 | 1.3 | 1.3 |
|  | NO | 77 | 30.7 | 33.8 | 35.1 |
|  | YES | 148 | 59.0 | 64.9 | 100.0 |
|  | Total | 228 | 90.8 | 100.0 |  |
| Missing | 99 | 1 | .4 |  |  |
|  | 999 | 22 | 8.8 |  |  |
|  | Total | 23 | 9.2 |  |  |
| Total | | 251 | 100.0 |  |  |

| **During the visits to care centers during your last pregnancy, were you informed about the signs of pregnancy complications, such as anemia, seizures, etc.?** | | | | | |
| --- | --- | --- | --- | --- | --- |
|  | | Frequency | Percent | Valid Percent | Cumulative Percent |
| Valid | I do not know‎ | 2 | .8 | .9 | .9 |
|  | NO | 132 | 52.6 | 58.1 | 59.0 |
|  | YES | 93 | 37.1 | 41.0 | 100.0 |
|  | Total | 227 | 90.4 | 100.0 |  |
| Missing | 99 | 2 | .8 |  |  |
|  | 999 | 22 | 8.8 |  |  |
|  | Total | 24 | 9.6 |  |  |
| Total | | 251 | 100.0 |  |  |

| **During the visits to care centers during your last pregnancy, were you informed about where you should go in case of complications?** | | | | | |
| --- | --- | --- | --- | --- | --- |
|  | | Frequency | Percent | Valid Percent | Cumulative Percent |
| Valid | I do not know‎ | 8 | 3.2 | 3.5 | 3.5 |
|  | NO | 124 | 49.4 | 54.9 | 58.4 |
|  | YES | 94 | 37.5 | 41.6 | 100.0 |
|  | Total | 226 | 90.0 | 100.0 |  |
| Missing | 99 | 3 | 1.2 |  |  |
|  | 999 | 22 | 8.8 |  |  |
|  | Total | 25 | 10.0 |  |  |
| Total | | 251 | 100.0 |  |  |

| **During the visits to care centers during your last pregnancy, were you provided with instructions on preparing for childbirth** | | | | | |
| --- | --- | --- | --- | --- | --- |
|  | | Frequency | Percent | Valid Percent | Cumulative Percent |
| Valid | I do not know‎ | 2 | .8 | .9 | .9 |
|  | NO | 138 | 55.0 | 60.8 | 61.7 |
|  | YES | 87 | 34.7 | 38.3 | 100.0 |
|  | Total | 227 | 90.4 | 100.0 |  |
| Missing | 99 | 2 | .8 |  |  |
|  | 999 | 22 | 8.8 |  |  |
|  | Total | 24 | 9.6 |  |  |
| Total | | 251 | 100.0 |  |  |

| ‏‫**During visits to care centers during your last pregnancy, were you provided with instructions on nutrition?** | | | | | |
| --- | --- | --- | --- | --- | --- |
|  | | Frequency | Percent | Valid Percent | Cumulative Percent |
| Valid | I do not know‎ | 3 | 1.2 | 1.3 | 1.3 |
|  | NO | 83 | 33.1 | 36.4 | 37.7 |
|  | YES | 142 | 56.6 | 62.3 | 100.0 |
|  | Total | 228 | 90.8 | 100.0 |  |
| Missing | 99 | 1 | .4 |  |  |
|  | 999 | 22 | 8.8 |  |  |
|  | Total | 23 | 9.2 |  |  |
| Total | | 251 | 100.0 |  |  |

| **During the visits to care centers during your last pregnancy, were you provided with instructions on breastfeeding?** | | | | | |
| --- | --- | --- | --- | --- | --- |
|  | | Frequency | Percent | Valid Percent | Cumulative Percent |
| Valid | I do not know‎ | 1 | .4 | .4 | .4 |
|  | NO | 158 | 62.9 | 69.3 | 69.7 |
|  | YES | 69 | 27.5 | 30.3 | 100.0 |
|  | Total | 228 | 90.8 | 100.0 |  |
| Missing | 99 | 1 | .4 |  |  |
|  | 999 | 22 | 8.8 |  |  |
|  | Total | 23 | 9.2 |  |  |
| Total | | 251 | 100.0 |  |  |

| ‏‫**During the visits to care centers during your last pregnancy, were you able to ask any questions?** | | | | | |
| --- | --- | --- | --- | --- | --- |
|  | | Frequency | Percent | Valid Percent | Cumulative Percent |
| Valid | I do not know‎/ I do not remember | 1 | .4 | .4 | .4 |
|  | Never | 13 | 5.2 | 5.8 | 6.2 |
|  | few times | 23 | 9.2 | 10.2 | 16.4 |
|  | most times | 22 | 8.8 | 9.7 | 26.1 |
|  | every times | 167 | 66.5 | 73.9 | 100.0 |
|  | Total | 226 | 90.0 | 100.0 |  |
| Missing | 99 | 3 | 1.2 |  |  |
|  | 999 | 22 | 8.8 |  |  |
|  | Total | 25 | 10.0 |  |  |
| Total | | 251 | 100.0 |  |  |

| **During the visits to care centers during your last pregnancy, were you able to discuss your concerns privately?** | | | | | |
| --- | --- | --- | --- | --- | --- |
|  | | Frequency | Percent | Valid Percent | Cumulative Percent |
| Valid | I do not know‎/ I do not remember | 1 | .4 | .4 | .4 |
|  | Never | 23 | 9.2 | 10.1 | 10.5 |
|  | few times | 21 | 8.4 | 9.2 | 19.7 |
|  | most times | 24 | 9.6 | 10.5 | 30.3 |
|  | every times | 159 | 63.3 | 69.7 | 100.0 |
|  | Total | 228 | 90.8 | 100.0 |  |
| Missing | 99 | 1 | .4 |  |  |
|  | 999 | 22 | 8.8 |  |  |
|  | Total | 23 | 9.2 |  |  |
| Total | | 251 | 100.0 |  |  |

| **During the visits to care centers during your last pregnancy, did you feel that the healthcare provider treated you with respect?** | | | | | |
| --- | --- | --- | --- | --- | --- |
|  | | Frequency | Percent | Valid Percent | Cumulative Percent |
| Valid | I do not know‎/ I do not remember | 1 | .4 | .4 | .4 |
|  | Never | 12 | 4.8 | 5.3 | 5.7 |
|  | few times | 5 | 2.0 | 2.2 | 7.9 |
|  | most times | 12 | 4.8 | 5.3 | 13.2 |
|  | every times | 198 | 78.9 | 86.8 | 100.0 |
|  | Total | 228 | 90.8 | 100.0 |  |
| Missing | 99 | 1 | .4 |  |  |
|  | 999 | 22 | 8.8 |  |  |
|  | Total | 23 | 9.2 |  |  |
| Total | | 251 | 100.0 |  |  |

| **During the visits to care centers during your last pregnancy, did you feel that the healthcare provider treated you differently for a personal reason?** | | | | | |
| --- | --- | --- | --- | --- | --- |
|  | | Frequency | Percent | Valid Percent | Cumulative Percent |
| Valid | I do not know‎/ I do not remember | 4 | 1.6 | 1.8 | 1.8 |
|  | Never | 189 | 75.3 | 82.9 | 84.6 |
|  | few times | 2 | .8 | .9 | 85.5 |
|  | most times | 3 | 1.2 | 1.3 | 86.8 |
|  | every times | 30 | 12.0 | 13.2 | 100.0 |
|  | Total | 228 | 90.8 | 100.0 |  |
| Missing | 99 | 1 | .4 |  |  |
|  | 999 | 22 | 8.8 |  |  |
|  | Total | 23 | 9.2 |  |  |
| Total | | 251 | 100.0 |  |  |

| **During the visits to care centers during your last pregnancy, did you feel that the facility (the maternity care center) was clean?** | | | | | |
| --- | --- | --- | --- | --- | --- |
|  | | Frequency | Percent | Valid Percent | Cumulative Percent |
| Valid | I do not know‎/ I do not remember | 3 | 1.2 | 1.3 | 1.3 |
|  | Never | 17 | 6.8 | 7.5 | 8.8 |
|  | few times | 7 | 2.8 | 3.1 | 11.8 |
|  | most times | 23 | 9.2 | 10.1 | 21.9 |
|  | every times | 178 | 70.9 | 78.1 | 100.0 |
|  | Total | 228 | 90.8 | 100.0 |  |
| Missing | 99 | 1 | .4 |  |  |
|  | 999 | 22 | 8.8 |  |  |
|  | Total | 23 | 9.2 |  |  |
| Total | | 251 | 100.0 |  |  |

| **Have you been circumcised (FGM)?** | | | | | |
| --- | --- | --- | --- | --- | --- |
|  | | Frequency | Percent | Valid Percent | Cumulative Percent |
| Valid | no | 4 | 1.6 | 1.6 | 1.6 |
|  | yes | 246 | 98.0 | 98.0 | 99.6 |
|  | I do not know | 1 | .4 | .4 | 100.0 |
|  | Total | 251 | 100.0 | 100.0 |  |

| **WHO_recommendation_for_ANC_visits** | | | | | |
| --- | --- | --- | --- | --- | --- |
|  | | Frequency | Percent | Valid Percent | Cumulative Percent |
| Valid | no | 165 | 65.7 | 65.7 | 65.7 |
|  | yes | 86 | 34.3 | 34.3 | 100.0 |
|  | Total | 251 | 100.0 | 100.0 |  |

**Reliability**

| **Notes** | | |
| --- | --- | --- |
| Output Created | | 02-MAY-2023 14:07:47 |
| Comments | |  |
| Input | Data | C:\Users\hp\Documents\elmnagel medical day\SPSS DATA\ANC CLEANED Eyad.sav |
|  | Active Dataset | DataSet1 |
|  | Filter | <none> |
|  | Weight | <none> |
|  | Split File | <none> |
|  | N of Rows in Working Data File | 251 |
|  | Matrix Input |  |
| Missing Value Handling | Definition of Missing | User-defined missing values are treated as missing. |
|  | Cases Used | Statistics are based on all cases with valid data for all variables in the procedure. |
| Syntax | | RELIABILITY  /VARIABLES=During visits to the care centres during your last pregnancy did they take your weight?  During visits to the care centres during your last pregnancy did they take your blood pressure?  During visits to the care centres during your last pregnancy did they make any urine tests?  During visits to the care centres during your last pregnancy did they make any blood test?  During visits to the care centres during your last pregnancy were you informed of the purpose of the tests being done?  During visits to the care centres during your last pregnancy did you understand the purpose of the drugs being prescribed to you?  During visits to the care centres during your last pregnancy did they inform you about the natural changes to expect during pregnancy (nausea, vomiting)?  During visits to the care centres during your last pregnancy did they inform you about the signs of complications in pregnancy (anaemia, seizures)?  During the visits to the care centres during your last pregnancy, were you informed where you should go in case of complications?  During the visits to the care centres during your last pregnancy, were you given instructions regarding preparation for labour?  During the visits to the care centres during your last pregnancy, were you given instructions about nutrition?  During the visits to the care centres during your last pregnancy, were you given instructions regarding breastfeeding?  During the visits to the care centres during your last pregnancy, did you feel comfortable asking all the questions you wanted?  During the visits to the care centres during your last pregnancy, were you able to discuss your concerns privately?  During the visits to the care centres during your last pregnancy, did you feel that the healthcare provider treated you with respect?  During the visits to the care centres during your last pregnancy, did you feel that the healthcare provider treated you differently for a personal reason? Did you feel that you were treated differently from others?  During the visits to the care centres during your last pregnancy, did you feel that the facility (maternity care centre) was clean?  /SCALE('quality cronpach') ALL  /MODEL=ALPHA  /STATISTICS=DESCRIPTIVE SCALE CORR  /SUMMARY=TOTAL MEANS CORR. |
| Resources | Processor Time | 00:00:00.00 |
|  | Elapsed Time | 00:00:00.00 |

**Scale: quality cronpach**

| **Case Processing Summary** | | | |
| --- | --- | --- | --- |
|  | | N | % |
| Cases | Valid | 222 | 88.4 |
|  | Excluded^a^ | 29 | 11.6 |
|  | Total | 251 | 100.0 |

| a. Listwise deletion based on all variables in the procedure. |
| --- |

| **Reliability Statistics** | | |
| --- | --- | --- |
| Cronbach's Alpha | Cronbach's Alpha Based on Standardized Items | N of Items |
| .646 | .673 | 17 |

| **Item Statistics** | | | |
| --- | --- | --- | --- |
|  | Mean | Std. Deviation | N |
| During visits to the care centres during your last pregnancy did they take your weight? | 1.43 | 1.012 | 222 |
| During visits to the care centres during your last pregnancy did they take your blood pressure? | 3.67 | .794 | 222 |
| During visits to the care centres during your last pregnancy did they make any urine tests? | 3.64 | .805 | 222 |
| During visits to the care centres during your last pregnancy did they make any blood test? | 3.68 | .751 | 222 |
| During visits to the care centres during your last pregnancy were you informed of the purpose of the tests being done? | 2.65 | 1.402 | 222 |
| During visits to the care centres during your last pregnancy did you understand the purpose of the drugs being prescribed to you? | 3.31 | 1.172 | 222 |
| During visits to the care centres during your last pregnancy did they inform you about the natural changes to expect during pregnancy (nausea, vomiting)? | 1.64 | .507 | 222 |
| During visits to the care centres during your last pregnancy did they inform you about the signs of complications in pregnancy (anaemia, seizures)? | 1.41 | .501 | 222 |
| During the visits to the care centres during your last pregnancy, were you informed where you should go in case of complications? | 1.39 | .549 | 222 |
| During the visits to the care centres during your last pregnancy, were you given instructions regarding preparation for labour? | 1.39 | .497 | 222 |
| During the visits to the care centres during your last pregnancy, were you given instructions about nutrition? | 1.61 | .506 | 222 |
| During the visits to the care centres during your last pregnancy, were you given instructions regarding breastfeeding? | 1.30 | .460 | 222 |
| During the visits to the care centres during your last pregnancy, did you feel comfortable asking all the questions you wanted? | 3.53 | .911 | 222 |
| During the visits to the care centres during your last pregnancy, were you able to discuss your concerns privately? | 3.39 | 1.044 | 222 |
| During the visits to the care centres during your last pregnancy, did you feel that the healthcare provider treated you with respect? | 3.74 | .781 | 222 |
| During the visits to the care centres during your last pregnancy, did you feel that the healthcare provider treated you differently for a personal reason? Did you feel that you were treated differently from others? | 1.40 | 1.040 | 222 |
| During the visits to the care centres during your last pregnancy, did you feel that the facility (maternity care centre) was clean? | 3.56 | .967 | 222 |

| **Scale Statistics** | | | |
| --- | --- | --- | --- |
| Mean | Variance | Std. Deviation | N of Items |
| 42.73 | 31.356 | 5.600 | 17 |

RELIABILITY

/VARIABLES=‏‫

How does Antenatal care affect the health of the mother and the baby? Do you usually forget your appointment dates? Can you get the information you need about pregnancy from family or friends? Are maternity healthcare appointments usually too short and rushed? Are the working hours of the maternity care centre convenient for you? Are there long waiting times at the maternity care centre? Do you trust your healthcare provider? Do you think the people who monitor your pregnancy understand your health issues? Do you have someone to take care of your children while you go to the maternity care centre? Do you have other work commitments that take priority over Antenatal care? Does your husband have a negative view of Antenatal care services? Does your mother have a negative view of Antenatal care services? Do your peers have a negative view of Antenatal care services? Does attending the maternity care centre take up a lot of your time? Does attending the maternity care centre cost a lot of money? Is attending the maternity care centre physically exhausting?

/SCALE('drivers and barriers cronpach') ALL

/MODEL=ALPHA

/STATISTICS=DESCRIPTIVE SCALE CORR

/SUMMARY=TOTAL MEANS CORR.

**Reliability**

| **Notes** | | |
| --- | --- | --- |
| Output Created | | 02-MAY-2023 14:04:51 |
| Comments | |  |
| Input | Data | C:\Users\hp\Documents\elmnagel medical day\SPSS DATA\ANC CLEANED Eyad.sav |
|  | Active Dataset | DataSet1 |
|  | Filter | <none> |
|  | Weight | <none> |
|  | Split File | <none> |
|  | N of Rows in Working Data File | 251 |
|  | Matrix Input |  |
| Missing Value Handling | Definition of Missing | User-defined missing values are treated as missing. |
|  | Cases Used | Statistics are based on all cases with valid data for all variables in the procedure. |
| Syntax | | RELIABILITY  /VARIABLES= How does Antenatal care affect the health of the mother and the baby? Do you usually forget your appointment dates? Can you get the information you need about pregnancy from family or friends? Are maternity healthcare appointments usually too short and rushed? Are the working hours of the maternity care centre convenient for you? Are there long waiting times at the maternity care centre? Do you trust your healthcare provider? Do you think the people who monitor your pregnancy understand your health issues? Do you have someone to take care of your children while you go to the maternity care centre? Do you have other work commitments that take priority over Antenatal care? Does your husband have a negative view of Antenatal care services? Does your mother have a negative view of Antenatal care services? Do your peers have a negative view of Antenatal care services? Does attending the maternity care centre take up a lot of your time? Does attending the maternity care centre cost a lot of money? Is attending the maternity care centre physically exhausting?  /SCALE('drivers and barriers cronpach') ALL  /MODEL=ALPHA  /STATISTICS=DESCRIPTIVE SCALE CORR  /SUMMARY=TOTAL MEANS CORR. |
| Resources | Processor Time | 00:00:00.02 |
|  | Elapsed Time | 00:00:00.02 |

**Scale: drivers and barriers cronpach**

| **Case Processing Summary** | | | |
| --- | --- | --- | --- |
|  | | N | % |
| Cases | Valid | 242 | 96.4 |
|  | Excluded^a^ | 9 | 3.6 |
|  | Total | 251 | 100.0 |

| a. Listwise deletion based on all variables in the procedure. |
| --- |

| **Reliability Statistics** | | |
| --- | --- | --- |
| Cronbach's Alpha | Cronbach's Alpha Based on Standardized Items | N of Items |
| .643 | .640 | 16 |

| **Item Statistics** | | | |
| --- | --- | --- | --- |
|  | Mean | Std. Deviation | N |
| How does Antenatal care affect the health of the mother and the baby? | .99 | .288 | 242 |
| Do you usually forget your appointment dates? | 1.35 | .708 | 242 |
| Can you get the information you need about pregnancy from family or friends? | 1.64 | .674 | 242 |
| Are maternity healthcare appointments usually too short and rushed? | 1.79 | .779 | 242 |
| Are the working hours of the maternity care centre convenient for you? | 1.89 | .504 | 242 |
| Are there long waiting times at the maternity care centre? | 1.85 | .708 | 242 |
| Do you trust your healthcare provider? | 1.95 | .388 | 242 |
| Do you think the people who monitor your pregnancy understand your health issues? | 1.95 | .409 | 242 |
| Do you have someone to take care of your children while you go to the maternity care centre? | 1.82 | .611 | 242 |
| Do you have other work commitments that take priority over Antenatal care? | 1.21 | .522 | 242 |
| Does your husband have a negative view of Antenatal care services? | 1.10 | .355 | 242 |
| Does your mother have a negative view of Antenatal care services? | 1.07 | .334 | 242 |
| Do your peers have a negative view of Antenatal care services? | 1.16 | .418 | 242 |
| Does attending the maternity care centre take up a lot of your time? | 1.51 | .671 | 242 |
| Does attending the maternity care centre cost a lot of money? | 1.67 | .756 | 242 |
| Is attending the maternity care centre physically exhausting? | 1.43 | .616 | 242 |

| **Summary Item Statistics** | | | | | | | |
| --- | --- | --- | --- | --- | --- | --- | --- |
|  | Mean | Minimum | Maximum | Range | Maximum / Minimum | Variance |  |
| Item Means | 1.523 | .992 | 1.946 | .955 | 1.963 | .116 |  |
| Inter-Item Correlations | .100 | -.200 | .746 | .946 | -3.722 | .028 |  |

| **Item-Total Statistics** | | | | | |
| --- | --- | --- | --- | --- | --- |
|  | Scale Mean if Item Deleted | Scale Variance if Item Deleted | Corrected Item-Total Correlation | Squared Multiple Correlation |  |
| How does Antenatal care affect the health of the mother and the baby? | 23.37 | 12.857 | .051 | .080 |  |
| Do you usually forget your appointment dates? | 23.02 | 11.817 | .149 | .131 |  |
| Can you get the information you need about pregnancy from family or friends? | 22.72 | 12.062 | .113 | .130 |  |
| Are maternity healthcare appointments usually too short and rushed? | 22.57 | 10.801 | .320 | .186 |  |
| Are the working hours of the maternity care centre convenient for you? | 22.47 | 11.719 | .311 | .373 |  |
| Are there long waiting times at the maternity care centre? | 22.51 | 11.114 | .303 | .294 |  |
| Do you trust your healthcare provider? | 22.42 | 11.879 | .380 | .417 |  |
| Do you think the people who monitor your pregnancy understand your health issues? | 22.42 | 11.423 | .527 | .441 |  |
| Do you have someone to take care of your children while you go to the maternity care centre? | 22.55 | 12.207 | .109 | .107 |  |
| Do you have other work commitments that take priority over Antenatal care? | 23.16 | 11.867 | .252 | .157 |  |
| Does your husband have a negative view of Antenatal care services? | 23.26 | 12.650 | .107 | .639 |  |
| Does your mother have a negative view of Antenatal care services? | 23.30 | 12.699 | .098 | .591 |  |
| Do your peers have a negative view of Antenatal care services? | 23.21 | 12.646 | .076 | .382 |  |
| Does attending the maternity care centre take up a lot of your time? | 22.86 | 10.846 | .396 | .252 |  |
| Does attending the maternity care centre cost a lot of money? | 22.70 | 10.452 | .413 | .239 |  |
| Is attending the maternity care centre physically exhausting? | 22.93 | 10.776 | .468 | .299 |  |

| **Scale Statistics** | | | |
| --- | --- | --- | --- |
| Mean | Variance | Std. Deviation | N of Items |
| 24.36 | 13.046 | 3.612 | 16 |
